# The Usefulness of Known Genes/Qtls for Grain Quality Traits in an *Indica* Population of Diverse Breeding Lines Tested using Association Analysis

**DOI:** 10.1186/s12284-015-0064-3

**Published:** 2015-09-21

**Authors:** Xiangqian Zhao, Lijie Zhou, Kimberley Ponce, Guoyou Ye

**Affiliations:** Plant Breeding, Genetics and Biotechnology Division, International Rice Research Institute (IRRI), Los Baños, Laguna Philippines; Present address: Longping Branch, Graduate School of Central South University, Changsha, 410125 Hunan China

**Keywords:** Association analysis, Starch synthesis genes, Chalkiness, Grain quality, Rice

## Abstract

**Background:**

A number of studies reported major genes/QTLs for rice grain shapes, chalkiness and starch physicochemical properties. For these finely mapped QTLs or cloned genes to make an impact in practical breeding, it is necessary to test their effects in different genetic backgrounds. In this study, two hundred nineteen markers for 20 starch synthesis genes, 41 fine mapped grain shape and related traits QTLs/genes, and 54 chalkiness QTLs/genes plus 15 additional markers and a large *indica* population of 375 advanced lines were used to identify marker-trait associations under 6 environments that can be used directly in breeding for grain quality traits.

**Results:**

The significant associations detected by the QK model were used to declare the usefulness of the targeted genes/QTLs. A total of 65 markers were detected associations with grain quality trait at least in one environment. More phenotypic variations could be explained by haplotype than single marker, as exemplified by the starch biosynthesising genes. *GBSSI* was the major gene for AC and explained up to 55 % of the phenotypic variation, which also affected GC and accounted up to 11.31 % of the phenotypic variation. *SSIIa* was the major gene for chalkiness and explained up to 17 and 21 % of variation of DEC and PGWC, respectively. In addition, RMw513 and RM18068 were associated with DEC in 6 environments as well. Four markers (RGS1, RM15206, RMw513 and Indel1) tightly linked to *GS3*, *gw5*, and *qGL7-2* were the most important ones for grain shapes. Allelic combinations between *SSIIa* and RMw513 revealed more variations in DEC.

**Conclusions:**

The validated markers for genes/QTLs with major effects could be directly used in breeding for grain quality *via* marker-assisted selection. Creating desirable allelic combinations by gene pyramiding might be an effective approach for the development of high quality breeding lines in rice.

**Electronic supplementary material:**

The online version of this article (doi:10.1186/s12284-015-0064-3) contains supplementary material, which is available to authorized users.

## Background

Rice grain quality, influencing consumer demand and international trade, is a combination of physical and chemical characteristics. The growing demand for high yielding rice coupled with superior grain quality has become more and more urgent in rice breeding (Fitzgerald et al. 2009; Sreenivasulu et al. 2015). Generally speaking, grain quality has four major aspects, appearance, cooking, milling and nutrition quality. Preferences for some of the quality characteristics vary across countries and regions (Calingacion et al. 2014), but consumers prefer rice with uniform shape and translucent endosperm, therefore appearance quality (head rice, grain shape and chalkiness) directly affects consumer acceptance (Zhao and Fitzgerald 2013). Amylose content (AC) is the most important chemical characteristic determining eating quality and affecting some physical traits. AC was also reported to associate with chalkiness in large size of breeding lines or varieties in some studies (Zhou et al. 2015a, b). Starch granules in the chalky, opaque areas are loosely packed, and chalky kernels of the same genotype has less AC compared with the translucent rice kernels (Lisle et al. 2000; Patindol and Wang, 2003). Amylopectin might be involved as well (Ong and Blanshard, 1995). Amylose and amylopectin are the two major components of rice grain, which are biosynthesized by four major classes of starch synthesis enzymes, i.e., ADP-Glucose pyrophosphorylase, starch synthase, starch branching enzymes and starch de-branching enzymes (Hirose and Terao, 2004; Ohdan et al. 2005). Grain width (GW) had positive and high correlation with chalkiness (Adu-Kwarteng et al. 2003; Raju and Srinivas, 1991; Zhou et al. 2015a). GW also had the negative effects on AC but positive effects on gel consistency (GC), while grain length (GL) and length width ratio (LWR) were positively correlated with AC but negatively with GC (Xu et al. 2004).

A number of studies reported major genes for starch physicochemical properties in rice. It is well known that *GBSSI* is the major gene responsible for AC and GC (Cai et al. 1998; Chen et al. 2008; Tran et al. 2011), and *SSIIa* for gelatinization temperature and amylopectin chain length distribution (Bao et al. 2006; Umemoto et al. 2002). Beside these two genes, the roles, functions and expression patterns of other genes in rice starch biosynthesis have also been revealed by mutant or antisense inhibition analysis (Ohdan et al. 2005; Vandeputte and Delcour, 2004). Linkage mapping using biparental populations derived from crosses between genotypes with contrast phenotypic traits has played a critical role in dissecting the genetic architecture of grain shape and chalkiness in rice. About 40 QTLs related to GL, GW, grain weight and other yield related traits have been fine mapped (Summarized in Additional file 1: Table S4). So far a few QTLs for GL and GW have been cloned (Fan et al. 2006; Song et al. 2007; Weng et al. 2008, Li et al. 2011). Combinations of two GL and two GW QTLs produced diverse grain shapes, which provide the ability to adjust grain size to satisfy different consumer preferences (Bai et al. 2010). Many QTLs for chalkiness or related components were also identified (http://www.gramene.org). A stable QTL for chalkiness, mapped across four testing locations in two seasons using a population of chromosome segment substitution lines, has been fine-mapped within 140 kb (Guo et al. 2011; Wan et al. 2005). A recent study has identified a gene, *chalk5*, which significantly reduces chalkiness in rice grain (Li et al. 2014). Association mapping has also been successfully employed to identify marker-trait associations in rice (Huang et al. 2010; Huang et al. 2015; Zhao et al. 2010). More recently, some SNPs/InDels of the starch biosynthesizing genes have been reported as highly associated with starch physicochemical properties using *waxy* rice (Xu et al. 2013) and *japonica* breeding lines (Kharabian-Masouleh et al. 2012) and collections (Tian et al. 2009; Yang et al. 2014). Interestingly, some of the starch synthsis genes were found to affect not only physicochemical traits but chalkiness formation as well (Kharabian-Masouleh et al. 2012). Similar study has not yet been reported in *indica* populations.

For markers linked to these finely mapped QTLs or cloned genes to make an impact in practical breeding, it is necessary to test their effects in different genetic backgrounds. In this study, we conduct targeted association analysis in a diverse *indica* rice population of advanced breeding lines or released varieties from many irrigated rice breeding programs in different countries, representing the diversity within the elite breeding gene pools for irrigated ecosystem, to test the usefulness of markers on starch synthesis genes, finely mapped QTLs/genes for grain shape and chalkiness in breeding. In addition, the average N fertilizer application per hectare varies greatly across countries, ranging from 3–4 kg in Lao PDR to about 180 kg in China (http://www.fao.org/docrep/006/y4751e/y4751e0k.htm). Many studies reported that N fertilizer application affects rice grain quality such as milling and nutritional quality because of the adverse effect on occurrence of imperfect grains (Leesawatwong et al. 2005; Ning et al. 2009; Perez et al. 1996; Qiao et al. 2011). Our previous study reported that with increasing N rates, head rice yield (HRY) were improved in the wet season (WS), and chalkiness was decreased in both seasons. The average HRY was 10 % higher in the dry season (DS) than in the WS (Zhou et al. 2015a). Therefore, the stable results from different seasons and nitrogen treatments might provide valuable information to rice grain quality improvement using marker-assisted selection (MAS).

## Results

### Variation of Grain Quality Traits

A total of 392 rice breeding lines were field tested in the 2012 DS and WS of the IRRI headquarters (Los Baños, Philippines) with 3 nitrogen rates (0, 90 and 180 kg N in the DS; 0, 45 and 90 kg N in the WS). The nitrogen and season combinations were designated as DS_N0_, DS_N90_, DS_N180_, WS_N0_, WS_N45_ and WS_N90_. The population structure of 375 lines was estimated using STRCUTURE on 53 well amplified SSR markers evenly distributed on 12 chromosomes (Additional file 1: Table S2). The best value of number of subpopulations was determined by lnP(d) (log posterior probability of data) as described by Evanno et al. (2005). Based on the membership probability, 78, 113, 71 and 113 lines were grouped into Pop1, Pop2, Pop3 and Pop4, respectively. The proportion of IRRI breeding lines in Pop1 was 93.59 % (73 of 78), while it was only 30.09 % in Pop4. Significant differences among subpopulations were observed for all traits in all environments except HRY in DS_N180_ and WS_N90_ and degree of endosperm chalkiness (DEC) in DS_N180_ (Table 1). Pop4 had lower AC, DEC, GC, GL, LWR and percentage of grain with chalkiness (PGWC), and higher GW than other subpopulations, while the Pop1 had the highest GL and LWR, and the lowest GW in all the 6 environments. Pop1 was higher in DEC and PGWC, and lower in HRY in some environments. Pop2 had the highest AC in 5 environments (Table 1).

**Table 1:** Average trait values for eight grain quality traits of four subpopulations of an *indica* rice population in six environments

Trait	Subpopulation	DS_N0_	DS_N90_	DS_N180_	WS_N0_	WS_N45_	WS_N90_
AC	Pop1	22.84ab	22.84ab	22.61a	22.84ab	22.10ab	22.88a
	Pop2	23.48a	23.55a	23.01a	23.17ab	22.87a	22.98a
	Pop3	23.43a	23.52a	22.93a	23.46a	22.55ab	22.81a
	Pop4	21.99b	22.04b	21.50b	21.98b	21.39b	21.54b
DEC	Pop1	26.85a	17.67a	11.03a	30.72a	25.19a	20.01a
	Pop2	23.28a	17.85a	11.00a	26.31b	21.43ab	18.17ab
	Pop3	23.83a	17.34a	11.76a	26.90ab	21.91ab	21.61a
	Pop4	17.72b	13.42b	9.39a	19.91c	19.42b	15.89b
GC	Pop1	83.99ab	85.80a	86.94a	88.10a	80.49a	81.73a
	Pop2	79.19b	81.29a	82.05a	82.86b	78.01ab	74.86b
	Pop3	85.90a	85.29a	87.27a	88.03a	82.28a	81.03a
	Pop4	73.04c	75.27b	74.36b	76.81c	74.30b	74.64b
GL	Pop1	6.71a	6.71a	6.79a	6.75a	6.79a	6.81a
	Pop2	6.56b	6.57b	6.66bc	6.62bc	6.67bc	6.68bc
	Pop3	6.65a	6.67a	6.75ab	6.69ab	6.72a	6.74ab
	Pop4	6.52b	6.54b	6.58c	6.57c	6.63c	6.63c
GW	Pop1	2.04c	2.03c	2.04b	2.02b	2.04b	2.05b
	Pop2	2.10b	2.09ab	2.08b	2.06b	2.07ab	2.08ab
	Pop3	2.07b	2.06bc	2.07b	2.05b	2.08ab	2.09ab
	Pop4	2.15a	2.13a	2.14a	2.09a	2.11a	2.12a
HRY	Pop1	51.48b	52.36b	55.02a	38.47b	42.29b	44.32a
	Pop2	53.25b	54.49ab	54.66a	43.27a	44.35ab	43.3a
	Pop3	56.60a	55.50a	57.05a	41.89ab	47.05a	45.32a
	Pop4	57.88a	56.66a	56.80a	42.07a	46.21a	45.53a
LWR	Pop1	3.30a	3.32a	3.34a	3.36a	3.35a	3.34a
	Pop2	3.14bc	3.15bc	3.22bc	3.23bc	3.24b	3.22bc
	Pop3	3.22b	3.24ab	3.27ab	3.28b	3.24b	3.24b
	Pop4	3.07c	3.10c	3.10c	3.17c	3.17b	3.15c
PGWC	Pop1	89.31a	70.15a	51.17a	89.80a	84.76a	74.06ab
	Pop2	76.31b	65.81a	46.45ab	78.50b	72.48bc	67.87b
	Pop3	81.21ab	71.17a	51.42a	86.07ab	77.69ab	78.71a
	Pop4	61.89c	54.06b	40.44b	68.44c	66.19c	58.70c

Different letters in the same column indicate the difference was significant at *P* < 0.05

*AC* amylose content (%), *DEC* degree of endosperm chalkiness (%), *GC* gel consistency (mm), *GL* grain length (mm), *GW* grain width (mm), *HRY* head rice yield (%), *LWR* ratio of grain length to width and *PGWC* percentage of grain with chalkiness (%)

### Number of Markers Associated to Grain Quality Traits

A total of 79, 52, 68, 65, 64 and 42 significant marker-trait associations were identified for the eight traits using the QK (a mixed linear model adjusting for both population structure (Q) and genetic relatedness between genotypes (K)) model under DS_N0_, DS_N90_, DS_N180_, WS_N0_, WS_N45_ and WS_N90_ respectively (Table 2). More significant marker-trait associations were detected in N0 than in N90 for both of the DS and WS. N90 also had the lowest number of significant marker-trait associations in both seasons. For instance, about two times more significant associations were identified for DEC and GW in WS_N0_ than in WS_N90_. Such dramatic reduction was also observed for GC in DS. However, the numbers of significant associations for AC and PGWC varied less across the three nitrogen levels in both seasons. A total of 69 significant associations were detected for DEC, while only 18 for HRY (Table 2). The number of associated markers for each trait ranged from 10 (AC) to 29 (DEC), in total 65 markers were associated with trait at least in one environment. No marker was significantly associated with HRY in all the six environments. For the other seven traits, at least two marker-trait associations were commonly detected across environments (Additional file 1: Table S7).

**Table 2:** The number of markers associated with eight grain quality traits detected using QK model

Trait	DS_N0_	DS_N90_	DS_N180_	WS_N0_	WS_N45_	WS_N90_	Subtotal
AC	4	4	7	5	5	5	30/10
DEC	9	9	9	16	20	6	69/29
GC	23	9	5	5	6	4	52/28
GL	9	7	15	11	9	4	55/16
GW	8	7	9	8	6	2	40/12
HRY	3	1	1	1	0	12	18/16
LWR	15	8	14	12	9	5	63/20
PGWC	8	7	8	7	9	4	43/14
Subtotal	79	52	68	65	64	42	370/65

Trait abbreviations are as in Table 1

Markers with a significant marker-trait association are reported at *q* ^*FDR*^ < 0.05

Data before and after “/” represent number of total associations and number of markers associated in all 6 environments respectively

### Markers Associated with AC and GC

A total of 10 markers were significantly associated with AC in at least one of the six environments using the QK model (Table 2; Additional file 1: Table S7). Six of the 10 markers were on five starch biosynthesizing genes, *GBSSI*, *SSIIa*, *ISA2*, *SSI* and *SSIIIb* (Additional file 2: Figure S1). Three markers (RM111, RM204 and RM3414) were not on starch biosynthesizing genes but located between *GBSSI* and *SSIIa* on Chr06. In addition, one marker (RM21945) was on Chr07. Among these 10 markers, four markers were identified in three or more environments (Table 3; Additional file 1: Table S7). GBSSI-1-IF accounted for 34.36 ~ 44.55 % of the phenotypic variance with *q*^FDR^ value ranging from 5.20 × 10^−29^ to 3.05 × 10^−23^ across environments. GBSSI-4-IF could explain more than 18.00 % of the phenotypic variance. RM111 explained for 3.94 ~ 5.62 % of the phenotypic variance. SSIIa-IF was explained less than 4 % of the phenotypic variance.

**Table 3:** Marker loci associated with grain quality traits commonly detected using QK model

Trait	Marker	DS_N0_	DS_N90_	DS_N180_	WS_N0_	WS_N45_	WS_N90_
AC	GBSSI-4-IF	2.20 × 10^−15^/0.2218	7.95 × 10^−15^/0.2186	6.08 × 10^−17^/0.2497	7.22 × 10^−18^/0.2552	1.11 × 10^−12^/0.1809	2.50 × 10^−14^/0.2014
	GBSSI-1-IF	1.13 × 10^−26^/0.4087	5.11 × 10^−28^/0.4455	1.98 × 10^−26^/0.4114	5.20 × 10^−29^/0.4434	3.35 × 10^−24^/0.3643	3.05 × 10^−23^/0.3436
	RM111	1.66 × 10^−2^/0.0448	6.58 × 10^−3^/0.0513	1.02 × 10^−2^/0.0493	2.83 × 10^−3^/0.0531	3.47 × 10^−3^/0.0562	2.53 × 10^−2^/0.0394
	SSIIa-IF		3.88 × 10^−2^/0.0328	3.83 × 10^−2^/0.033	1.61 × 10^−2^/0.0343	3.50 × 10^−2^/0.0328	3.26 × 10^−2^/0.0307
GC	GBSSI-4-IF	4.11 × 10^−7^/0.0962	4.09 × 10^−7^/0.1094	2.85 × 10^−2^/0.0353	3.93 × 10^−5^/0.0704	5.70 × 10^−5^/0.0762	2.34 × 10^−3^/0.0515
	SSIIa-IF	3.01 × 10^−4^/0.0523	2.26 × 10^−3^/0.0516	2.35 × 10^−3^/0.0528	1.93 × 10^−2^/0.0346	1.50 × 10^−2^/0.0343	2.26 × 10^−3^/0.0539
	SSIIaSNP2	3.01 × 10^−4^/0.0459	2.26 × 10^−3^/0.0439	1.69 × 10^−3^/0.0475	1.93 × 10^−2^/0.0261	7.53 × 10^−3^/0.0347	2.26 × 10^−3^/0.0453
	SSIIa-F	3.01 × 10^−4^/0.0603	1.75 × 10^−2^/0.0447	1.66 × 10^−2^/0.0474	3.28 × 10^−2^/0.0344		1.32 × 10^−2^/0.0471
	RM204	9.64 × 10^−4^/0.0574	2.95 × 10^−2^/0.045			1.27 × 10^−2^/0.0503	
GL	RGS1	1.70 × 10^−21^/0.3241	4.44 × 10^−13^/0.2000	1.27 × 10^−17^/0.2617	3.44 × 10^−18^/0.2695	4.71 × 10^−12^/0.1752	1.30 × 10^−7^/0.1127
	RM5436.2	1.54 × 10^−4^/0.0967	8.15 × 10^−5^/0.105	9.17 × 10^−7^/0.1282	1.58 × 10^−5^/0.1111	3.13 × 10^−2^/0.0539	2.61 × 10^−2^/0.0609
	RM15206	2.97 × 10^−5^/0.0793	2.91 × 10^−3^/0.0518	2.47 × 10^−6^/0.0842	3.89 × 10^−7^/0.1042	1.24 × 10^−3^/0.0549	2.61 × 10^−2^/0.0348
	GBSSII-F	2.59 × 10^−3^/0.0408	4.15 × 10^−3^/0.0393	9.17 × 10^−7^/0.0853	1.67 × 10^−4^/0.0539	1.38 × 10^−2^/0.0306	
	GBSSII-IF	5.71 × 10^−3^/0.0416	1.05 × 10^−2^/0.0412	1.68 × 10^−6^/0.0884	5.23 × 10^−4^/0.0555	3.13 × 10^−2^/0.0306	
	RM5499	4.48 × 10^−3^/0.0593	6.23 × 10^−6^/0.1098	2.01 × 10^−6^/0.1036	9.48 × 10^−5^/0.0841	2.10 × 10^−2^/0.047	
	RM16	5.71 × 10^−3^/0.0550	3.64 × 10^−2^/0.0459	1.26 × 10^−2^/0.0449	8.32 × 10^−3^/0.0503		
	RM18751			1.26 × 10^−2^/0.0327	1.42 × 10^−2^/0.0341	1.65 × 10^−2^/0.0368	2.61 × 10^−2^/0.0341
	SSI-2-IF	7.48 × 10^−3^/0.0311		2.74 × 10^−3^/0.0342	5.15 × 10^−3^/0.0323	6.47 × 10^−3^/0.0355	
	RMw513	2.89 × 10^−2^/0.0488		2.74 × 10^−3^/0.0618	1.83 × 10^−3^/0.067		
GW	RGS1	4.28 × 10^−8^/0.1211	2.09 × 10^−4^/0.0679	8.96 × 10^−7^/0.1046	3.53 × 10^−7^/0.1087	5.67 × 10^−5^/0.0733	4.95 × 10^−3^/0.0498
	RMw513	1.58 × 10^−4^/0.0898	4.60 × 10^−4^/0.0839	1.92 × 10^−6^/0.1216	1.57 × 10^−6^/0.1213	5.67 × 10^−5^/0.0985	8.31 × 10^−4^/0.0857
	Indel1	1.81 × 10^−4^/0.0651	1.32 × 10^−4^/0.0739	1.58 × 10^−3^/0.0551	2.98 × 10^−3^/0.0498	2.99 × 10^−2^/0.033	
	RM21945	6.60 × 10^−3^/0.051	2.99 × 10^−3^/0.0572	6.13 × 10^−3^/0.0518	7.37 × 10^−3^/0.0506		
	RM21950	1.39 × 10^−4^/0.0705	1.32 × 10^−4^/0.073	5.44 × 10^−3^/0.0465	2.98 × 10^−3^/0.0511		
	SSI-2-IF	1.17 × 10^−2^/0.0304		1.29 × 10^−2^/0.0303	1.27 × 10^−2^/0.0299	2.89 × 10^−2^/0.0263	
	RM21964	7.21 × 10^−3^/0.0494	1.64 × 10^−2^/0.045	1.29 × 10^−2^/0.0389			
	RM18360	1.17 × 10^−2^/0.0382		1.43 × 10^−2^/0.0373	8.91 × 10^−3^/0.0407		
LWR	RGS1	2.44 × 10^−16^/0.236	2.27 × 10^−9^/0.1433	9.44 × 10^−13^/0.1892	3.84 × 10^−14^/0.206	1.13 × 10^−9^/0.1402	1.26 × 10^−5^/0.0874
	RMw513	6.58 × 10^−4^/0.0759	5.07 × 10^−3^/0.0673	2.45 × 10^−5^/0.1053	5.21 × 10^−6^/0.1132	9.74 × 10^−4^/0.0781	4.65 × 10^−3^/0.0705
	Indel1	1.27 × 10^−5^/0.0811	1.60 × 10^−4^/0.0722	6.99 × 10^−5^/0.0728	4.09 × 10^−4^/0.060	4.20 × 10^−3^/0.0441	4.13 × 10^−2^/0.0334
	RM15206	6.62 × 10^−4^/0.0537	1.12 × 10^−2^/0.0408	2.38 × 10^−4^/0.0642	9.68 × 10^−6^/0.0827	2.82 × 10^−3^/0.0485	2.19 × 10^−2^/0.0395
	SSI-2-IF	2.26 × 10^−3^/0.0365		2.27 × 10^−3^/0.0386	2.46 × 10^−3^/0.0376	4.04 × 10^−3^/0.0367	
	RM21945	1.69 × 10^−3^/0.0548	5.89 × 10^−3^/0.0525	1.41 × 10^−3^/0.0605	2.46 × 10^−3^/0.0534		
	RM21950	1.07 × 10^−4^/0.0667	8.32 × 10^−4^/0.0603	2.26 × 10^−3^/0.0483	1.04 × 10^−3^/0.0535		
	RM5436.2	6.12 × 10^−3^/0.0629	1.36 × 10^−3^/0.0834	1.91 × 10^−3^/0.0765	2.46 × 10^−3^/0.0728		
	RM5499		1.52 × 10^−2^/0.0513	2.58 × 10^−2^/0.0434	1.48 × 10^−2^/0.0482		
	GBSSII-F	4.76 × 10^−2^/0.0175		4.3 × 10^−2^/0.0347	2.35 × 10^−2^/0.0240		
	RM16	1.34 × 10^−2^/0.0463		3.87 × 10^−2^/0.0404			4.13 × 10^−2^/0.0454
DEC	SSIIa-F	6.08 × 10^−7^/0.1061	1.39 × 10^−4^/0.0791	5.38 × 10^−5^/0.0896	5.92 × 10^−7^/0.1066	8.73 × 10^−6^/0.0867	1.55 × 10^−5^/0.0888
	SSIIa-IF	3.24 × 10^−8^/0.1223	1.39 × 10^−4^/0.0737	2.88 × 10^−4^/0.0626	1.84 × 10^−10^/0.1535	1.65 × 10^−6^/0.0901	1.40 × 10^−8^/0.1297
	SSIIaSNP2	4.91 × 10^−7^/0.0905	2.17 × 10^−4^/0.0552	1.88 × 10^−4^/0.0588	1.19 × 10^−8^/0.1121	1.65 × 10^−6^/0.080	8.64 × 10^−7^/0.0894
	RMw513	1.98 × 10^−2^/0.0501	2.82 × 10^−4^/0.0847	3.81 × 10^−4^/0.0806	1.52 × 10^−2^/0.0529	2.44 × 10^−2^/0.0412	4.16 × 10^−2^/0.0489
	RM18068	4.64 × 10^−4^/0.070	3.27 × 10^−4^/0.0755	1.16 × 10^−2^/0.0503	1.15 × 10^−4^/0.0814	4.79 × 10^−2^/0.0282	3.43 × 10^−2^/0.0455
	GBSSI-1-IF	3.67 × 10^−6^/0.0849	5.47 × 10^−3^/0.0402		1.02 × 10^−3^/0.0528	5.16 × 10^−3^/0.0379	8.08 × 10^−4^/0.0561
	GBSSI-3-IF	4.74 × 10^−4^/0.0545	2.77 × 10^−2^/0.0292		1.52 × 10^−2^/0.0346	9.46 × 10^−3^/0.033	
	RI02451	8.25 × 10^−4^/0.0507	3.40 × 10^−3^/0.0444		1.01 × 10^−2^/0.0386	4.61 × 10^−4^/0.0525	
PGWC	SSIIa-F	8.22 × 10^−9^/0.1277	4.11 × 10^−6^/0.0993	6.33 × 10^−4^/0.069	5.04 × 10^−9^/0.1323	3.83 × 10^−6^/0.0957	1.35 × 10^−7^/0.1162
	SSIIa-IF	1.88 × 10^−12^/0.1761	1.49 × 10^−7^/0.1141	6.63 × 10^−5^/0.0779	2.99 × 10^−13^/0.1896	6.11 × 10^−8^/0.1171	1.17 × 10^−10^/0.1575
	SSIIaSNP2	2.94 × 10^−10^/0.1288	1.49 × 10^−7^/0.1003	6.63 × 10^−5^/0.0657	9.58 × 10^−11^/0.1347	4.80 × 10^−7^/0.0897	7.79 × 10^−9^/0.1152
	GBSSI-1-IF	1.01 × 10^−6^/0.089	3.13 × 10^−2^/0.0337		4.48 × 10^−5^/0.0689	8.57 × 10^−3^/0.0409	2.22 × 10^−4^/0.0625
	GBSSI-3-IF	1.81 × 10^−4^/0.0583	4.60 × 10^−2^/0.0308		2.23 × 10^−2^/0.0316	4.28 × 10^−2^/0.0284	
	RM18068	8.29 × 10^−3^/0.0486	2.72 × 10^−4^/0.0781	3.52 × 10^−3^/0.0622	3.60 × 10^−3^/0.0553		
	RGS1	9.21 × 10^−3^/0.0351			1.97 × 10^−3^/0.0462	3.72 × 10^−2^/0.0304	
	RI02451	8.03 × 10^−3^/0.0371	4.93 × 10^−3^/0.045	4.01 × 10^−2^/0.0302			

Markers associated with single trait more than 3 times using QK were presented on this table

The number before and after “/” represent *q* value and *R* ^*2*^ respectively. Markers with a significant marker-trait association are reported at *q* ^*FDR*^ < 0.05. *R* ^*2*^ indicates the percentage of the total variation explained by each locus

Trait abbreviations are as in Table 1

A total of 28 markers were associated with GC (Table 2; Additional file 1: Table S7). Among them, twenty two markers were located on nine starch biosynthesizing genes or very close to these genes (less than 2 Mb in physical map) (Additional file 2: Figure S1). Only five markers were associated with GC in three or more environments (Table 3; Additional file 1: Table S7). GBSSI-4-IF had the strongest association and accounted for 3.53 ~ 10.94 % of the phenotypic variance in all the six environments (Table 3). Three markers of the *SSIIa* gene (SSIIaSNP2, SSIIa-IF and SSIIa-F) explained 2.61 ~ 6.03 % of the variance in five or six environments. RM204, a SSR marker between *GBSSI* and *SSIIa*, was associated with GC in three environments and explained about 5 % of the variance.

### Markers Associated with Grain Shapes

A total of 16 markers were significantly associated with GL in at least one of the six environments (Table 2; Additional file 1: Table S7). Among them, 10 markers were associated with GL at least in three environments (Table 3). RGS1, an InDel marker on *GS3* gene, had the strongest association with the *q*^FDR^ value ranging from 1.70 × 10^−21^ to 1.30 × 10^−7^ and accounted for 11.27 ~ 32.41 % of the phenotypic variance across the six environments. RM15206, another marker closed to *GS3* also associated with GL in all the six environments. RM5436.2 and RM5499, two flanking markers for *Ghd7*, were highly associated with GL in six and five environments, respectively. Two markers on *GBSSII* (GBSSII-F and GBSSII-IF) were identified association with GL in five environments but with small effects. SSI-2-IF, RM16 and RM18751 were associated with GL in four environments. RMw513, a marker for *gw5*, *GS5* and *qGW5*, was associated with GL in three environments. The other six markers only associated with GL in one or two environments with relatively small effects (Additional file 1: Table S7).

Twelve markers associated with GW were detected (Table 2; Additional file 1: Table S7). Among them, 8 markers were associated with GW at least in three environments (Table 3). RGS1 and RMw513 accounted for more than 5 % of the phenotypic variance across the six environments. Indel1, RM21945, RM21950, SSI-2-IF, RM21964 and RM18360 with small effects were detected in three to five environments (Table 3).

Twenty markers were associated with LWR in at least one of the six environments (Table 2; Additional file 1: Table S7). Among them, 11 markers were identified in three or more environments. RGS1, RMw513, Indel1 and RM15206 associated with LWR in all the six environments. RGS1 had the strongest association and accounted for 8.74 ~ 23.60 % of the phenotypic variance with *q*^FDR^ values of 2.44 × 10^−16^ ~ 1.26 × 10^−5^. RMw513 also had strong effect and explained more than 6.75 % of the phenotypic variance. RM21945, RM21950 and RM5436.2 were associated with LWR in four environments and accounted for more than 5 % of the phenotypic variance. Other four markers, SSI-2-IF, RM5499, GBSSII-F and RM16 were associated with LWR in three or four environments with small effects. Additional nine markers associated with LWR in one or two environments were listed on Additional file 1: Table S7.

### Markers Associated with Chalkiness and HRY

Twenty nine markers were detected to be associated with DEC (Table 2; Additional file 1: Table S7). Among them, twenty one markers were very close to (less than 2 Mb) 26 known QTLs/genes regions for chalkiness (Additional file 2: Figure S1). Markers for the other 28 known QTLs/genes for chalkiness did not show significant associations with DEC. Eight markers were significant for DEC in three or more environments (Table 3). Three markers of *SSIIa* (SSIIaSNP2, SSIIa-IF and SSIIa-F), explained 5.52 ~ 15.35 % of the phenotypic variance across the six environments (Table 3). In the *SSIIa* region seven QTLs for chalkiness had been reported. Two markers on *GBSSI* (GBSSI-1-IF and GBSSI-3-IF), which is also close to the seven chalkiness QTLs, were highly associated with DEC as well. RMw513 and RM18068, linked to *chalk5*, were also associated with DEC and explained more than 5 % of the phenotypic variance in most environments. RI02451 located to the region of three QTLs for chalkiness was associated with DEC in four environments. Other 21 markers only associated with DEC in one or two environments with relatively small effects (Additional file 1: Table S7).

Fourteen markers were highly associated with PGWC (Table 2; Additional file 1: Table S7). Among them, eight markers were significant for PGWC in three or more environments (Table 3). Three markers on *SSIIa*, SSIIaSNP2, SSIIa-IF and SSIIa-F, could explain 6.57 ~ 18.96 % of the phenotypic variance in the six environments. GBSSI-1-IF, GBSSI-3-IF, RM18068, RGS1 and RI02451 were associated with PGWC in three to five environments with relatively small effects.

Sixteen markers were significantly associated with HRY (Table 2; Additional file 1: Table S7). No significant association was identified for HRY in three or more environments (Additional file 1: Table S7).

### Association Mapping using Haplotypes of the Starch Biosynthesizing Genes

Haplotype-based association analysis was conducted for the 15 starch biosynthesizing genes. Eight of the 15 genes were found to be associated with at least one trait in one environment. Four genes had strong associations with at least one trait in three or more environments (Table 4). *GBSSI* had very large effect on AC in all the six environments and explained up to 55 % of the phenotypic variation. It also had sizeable effect on GC in all environments and accounted for 4.25 to 11.31 % of the phenotypic variation. The effect of *GBSSI* on chalkiness was large as well, accounting for 4.85 ~ 9.53 and 5.15 ~ 10.34 % of the phenotypic variation on DEC and PGWC, respectively. *SSIIa* had a large effect on chalkiness in all the six environments and accounted for 10.41 ~ 17.05 % (9.33 ~ 21.10 %) of the phenotypic variation of DEC (PGWC). *SSIIa* also affected AC and explained 7.45 ~ 12.74 % of the phenotypic variation in the six environments. *GBSSII* had small effect on GL in five environments and explained up to 8.84 % of the phenotypic variation. *SSIVb* had small effect on DEC in three environments.

**Table 4:** Genes associated with eight grain quality traits commonly detected using haplotype-based association analysis with the QK model

Gene	Trait	DS_N0_	DS_N90_	DS_N180_	WS_N0_	WS_N45_	WS_N90_
*GBSSI*	AC	2.21 × 10^−28^	1.44 × 10^−29^	1.54 × 10^−28^	6.85 × 10^−32^	1.09 × 10^−25^	1.41 × 10^−25^
		0.4921	0.5309	0.5037	0.5517	0.4396	0.4323
	DEC	2.81 × 10^−5^	0.0229		1.78 × 10^−3^	8.76 × 10^−3^	0.0142
		0.0953	0.0485		0.0689	0.0521	0.0578
	GC	7.17 × 10^−7^	9.26 × 10^−6^	0.0100	2.55 × 10^−4^	6.02 × 10^−4^	0.0132
		0.1131	0.1117	0.0425	0.0772	0.0805	0.0577
	PGWC	4.27 × 10^−6^			5.75 × 10^−4^	0.0261	2.11 × 10^−3^
		0.1034			0.0744	0.0515	0.0690
*GBSSII*	GL	5.71 × 10^−3^	0.0105	1.68 × 10^−6^	5.23 × 10^−4^	0.0313	
		0.0416	0.0412	0.0884	0.0555	0.0306	
*SSIIa*	AC	9.26 × 10^−3^	6.36 × 10^−3^	9.92 × 10^−4^	1.47 × 10^−3^	0.0275	1.30 × 10^−5^
		0.0842	0.0880	0.1022	0.0938	0.0745	0.1274
	DEC	6.08 × 10^−7^	3.34 × 10^−4^	2.89 × 10^−4^	4.45 × 10^−8^	1.88 × 10^−4^	1.31 × 10^−6^
		0.1521	0.1052	0.1081	0.1705	0.1041	0.1497
	PGWC	3.46 × 10^−10^	1.63 × 10^−5^	3.22 × 10^−3^	9.58 × 10^−11^	1.41 × 10^−5^	1.18 × 10^−8^
		0.1978	0.1311	0.0933	0.2110	0.1269	0.1809
*SSIVb*	DEC		1.13 × 10^−2^	5.30 × 10^−3^		2.83 × 10^−2^	
			0.0422	0.0497		0.0292	

Trait abbreviations are as in Table 1

The data in upper and below row in each trait represent *q* ^*FDR*^ and *R* ^*2*^ respectively

### Allelic Effects

**Fig. 1:**
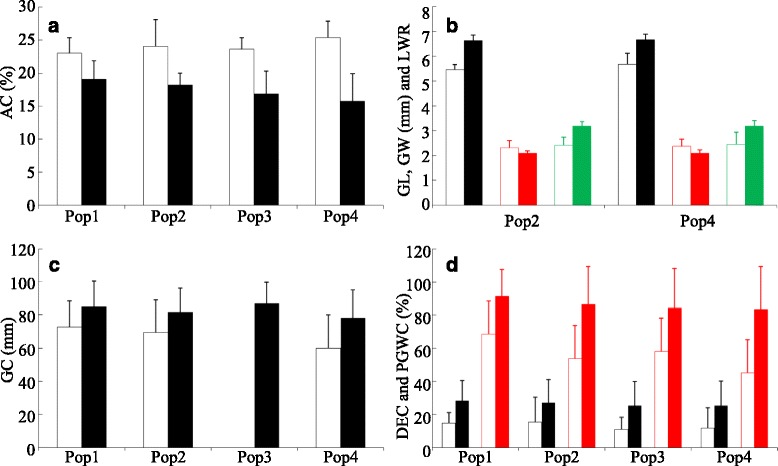
Comparisons of the mean values of grain quality traits between alleles. Pop1, Pop2, Pop3 and Pop4 represent four subpopulations identified by structure analysis, respectively. **a** Allele effects of GBSSI-1-IF on AC (%). **b** Allele effects of RGS1 on grain shape. *Black*, *red* and *green* represent GL (mm), GW (mm) and LWR, respectively. **c** Allele effects of GBSSI-4-IF on GC (mm). **d** Allele effect of SSIIa-IF on DEC (%) (*black*) and PGWC (%) (*red*)

Figure 1 gave the allelic effects of the major markers identified using the QK model in DS_N0._ Allelic effects in other 5 environments were given in Additional file 1: Table S8. Average AC value of genotypes carrying GBSSI-1-IF allele 1 was higher than that of genotypes carrying GBSSI-1-IF allele 2 in all subpopulations with the largest difference being about 10 % observed in Pop4 (Fig. 1a). Allele 1 of RGS1 caused shorter and wider grain with larger LWR in Pop2 and Pop4 (Fig. 1b). The allelic effects of RGS1 alleles were not estimated for the Pop1 and Pop3, since only one allele was present in Pop1 and one of the two alleles was carried by only one genotype in Pop3. Allele 1 of the GBSSI-4-IF decreased GC in all the four subpopulations (Fig. 1c) with the difference ranging from 12.36 to 18.36 mm, although the result for Pop3 was not given due to the very small number of genotypes carrying one of the alleles. The allele 1 of SSIIa-IF decreased DEC and PGWC in all the four subpopulations (Fig. 1d). The difference was more than 11 and 25 % for DEC and PGWC, respectively. Similar pattern for each trait was observed in the other five environments (Additional file 1: Table S8).

**Fig. 2:**
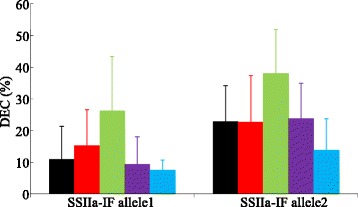
Effects of allele combinations between SSIIa-IF and RMw513 on DEC (%) in Pop4. Different colours represent 5 different alleles of RMw513, respectively

The pyramided effects of selected markers for different genes (listed in Table 3) were also investigated. However, some of the allelic combinations of the two genes (markers) for a single trait were not available in most of the subpopulations. Figure 2 was the effects of RMw513 and SSIIa-IF on DEC in Pop4 (Fig. 2). After removal of rare alleles, five alleles of RMw513 were used. For all the five RMw513 genotypes, lines carrying the allele 1 of SSIIa-IF had the lowest DEC. For the two SSIIa-IF genotypes, lines carrying the allele 3 of RMw513 had the highest DEC while those carrying the allele 5 had the lowest DEC (Fig. 2). SSIIa-IF and RMw513 was explained 16.41 and 20.80 % of phenotypic variation in Pop4, respectively, while combinations was accounted for 34.86 % of variation.

## Discussion

Among the 20 starch biosynthesizing genes, *GBSSI* had the largest effect on AC (Tables 3 and 4). Indeed, the SNPs on the first intron/exon junction site (GBSSI-1-IF) had the largest effects (Fig. 1a; Table 3; Additional file 1: Table S8). This finding was consistent with previous studies (Chen et al. 2008; Tian et al. 2009). *SSI*, *SSIIa*, *SSIIIb* and *ISA2* were also associated with AC in the present study (Additional file 1: Table S7; Additional file 2: Figure S1). *SSIIa*-AC association was detected in five environments, indicating that its effect on AC was relatively stable across environments. *SSI*, *SSIIIa* and *SSIVa* have been reported to affect AC in different populations (Kharabian-Masouleh et al. 2012; Yang et al. 2014), although there was no direct evidence showing these three genes are involved in amylose synthesis. It has been reported that *SSI*, *SSIIa* and *SSIII* contributed to the synthesis of very short chains, the elongation of A and B1 chains and the formation of long B1 and B2 chains of amylopectin, respectively (Nakamura, 2002; Umemoto et al. 2002; van de Wal et al. 1998), suggesting amylopectin chains are the primers for amylose formation. GBSSI-4-IF had also strong association with GC. This was consistent with previous studies showing that ‘C/T’ SNP on exon 10 was important in determining whether cooked rice with high AC is soft or firm textured (Tian et al. 2009; Tran et al. 2011). *SSIIa* was associated with GC across the six environments in present study, which is consistent with previous study (Yang et al. 2014).

Strong associations between *SSIIa* and chalkiness were found by the single marker based analysis and the haplotype based analysis (Tables 3 and 4). More phenotypic variation could be explained by differences in haplotypes. Many studies have revealed the effects of *SSIIa*, especially the GC/TT SNPs (SSIIa-IF) of exon 8, on gelatinization temperature, cooking time and cooking quality (Bao et al. 2006; Cuevas et al. 2010; Umemoto and Aoki, 2005). All three loci of the *SSIIa* gene, 9 bp deletion on 5'-UTR (SSIIa-F), GC/TT SNPs (SSIIa-IF) and G/A SNP (SSIIaSNP2), were associated with DEC and PGWC in all the six environments (Table 4). The phenotypic variation explained by *SSIIa* haplotype was about twice of that by *GBSSI* in our *indica* population. In addition, association between *SSIIa* and chalkiness was also reported in previous study using a panel of *japonica* advanced lines (Kharabian-Masouleh et al. 2012). Furthermore, chalkiness of RNAi-repressed *SSIIa* lines was increased significantly, from 11.4 ± 1.2 % (Wild type) to 28.4 ± 5.1 %, since the transgenetic kernels always contained white bellies or white backs and seed weight was decreased as well (Zhang et al. 2011). Therefore, *SSIIa* might be one of the important starch biosynthesizing genes for developing low chalky rice. Highly significant associations between *GBSSI* and DEC and PGWC were identified respectively in five and four environments by the haplotype analysis (Table 4). QTLs for chalkiness components were previously mapped to the *GBSSI* region using different mapping populations derived from *indica*/*indica* or *indica*/*japonica* crosses (Peng et al. 2014; Tan et al. 2000). AC and chalkiness was negatively correlated in the population used in this study, and a strong negative correlation between AC and chalkiness was also found in a population of 238 newly released *indica* varieties in China (Zhou et al. 2015a, b). Therefore, it appeared that *GBSSI* might be important for improving appearance quality in *indica* rice. However, the association between *GBSSI* and chalkiness was not identified in a population of 233 Australian *japonica* rice breeding lines (Kharabian-Masouleh et al. 2012).

*AGPS1*, *AGPS2*, *APGL2*, *BEI*, *BEIIa*, *ISA1*, *SSIIb*, *SSIIc*, *SSIIIa* and *SSIVa*, did not affect the eight traits in the present study (Additional file 2: Figure S1). *SSIIb* is mostly expressed in leaf and sheath, and therefore does not have effects on grain quality traits (Hirose and Terao, 2004). Studies using mutants or antisense inhibition demonstrated that *SSIIIa* and *ISA1* was involved in determining rice quality and structure of endosperm amylopectin, respectively (Fujita et al. 2007; Kawagoe et al. 2005; Nishi et al. 2001). However no significant association was found in the present study. It might be that the total deficiency of the enzyme activity in mutants or antisense inhibition plants caused much more dramatic changes in starch granule structure and resulted in significant effect on grain quality. The differences between natural alleles of the *SSIIIa* and *ISA1* present in our population might be too small to be detected. Ohdan et al. (2005) reported that *SSIVa* is at low transcript level at the early stage of grain filling and further decreases to a basal, barely detectable level, throughout the rest of grain development. Therefore, *SSIVa* may not have significant contribution to the phenotypic variation of quality traits related to mature grains (Ohdan et al. 2005).

Among the 80 markers tightly linked to the 41 fine mapped QTLs or cloned genes related to grain shape or yield, only 10 markers for 12 genes/QTLs were found to associate with grain shape traits in at least one environment (Additional file 1: Tables S4 and S7; Additional file 2: Figure S1). The effect of *GS3* on grain length has been extensively studied (Fan et al. 2006; Wang et al. 2011). Among the 6 markers tested for *GS3*, RGS1 was associated with GL, GW and LWR in all the six environments. RM15206 was associated with GL, GW and LWR in six, two and six environments, respectively (Additional file 1: Table S7). Therefore, RGS1 could be used to screen for grain shape. For Indel1 and RM21945, the two flanking markers of *qGL7-2*, 11 and 9 associations were detected for the three grain shape traits, implying that *qGL7-2* is very important to control grain shape in rice as well (Shao et al. 2010). The *gw5* gene is an important QTL controlling GW and LWR (Li et al. 2011; Wan et al. 2008; Weng et al. 2008). Among the eight markers for *gw5*, only RMw513 showed significant associations with GW and LWR in all the six environments. RMw513 was also highly associated with DEC, which might partially explain the significant positive correlation between GW and DEC in some genetic essence (Zhou et al. 2015a). RMw513 could be used in MAS for developing slender grain and low chalkiness. *GIF1* is responsible for grain weight reduction in rice (Wang et al. 2008). Two markers tightly linked to *GIF1*, Y48 and RM16942, were in association with grain shapes in some of the testing environments. RM20201, one of the three markers tightly linked to *gw6*, was found in association with GL in one environment. In addition, two flanking markers of *Gdh7*, RM5346.2 and RM5499, were highly associated with grain shape. The association between RM5346.2 and GL was significant in all the six environments with the *R*^*2*^ ranging from 0.0539 to 0.1282, suggesting it could be used for GL improvement (Table 3). No association was detected between the 25 markers for the 6 QTL clusters related to grain shapes on Chr01, Chr02, Chr03, Chr07, Chr08 and Chr09 (Additional file 1: Tables S4 and S7; Additional file 2: Figure S1). Indeed, 18 markers were not polymorphic or did not have enough number of lines carrying the rare allele. High density markers are required to test the effects of these QTLs or genes in the present population.

Seventy six makers for the 54 QTLs/genes for chalkiness reported in 17 published papers were used in the present study (Additional file 1: Table S5). Only 30 associations were found in all the six environments. RM18068 and RI02451 linked to the QTL clusters on Chr05 and Chr01, contributed six (four) and four (three) significant associations with DEC (PGWC) with small *R*^*2*^ value, respectively (Table 3; Additional file 1: Table S7; Additional file 2: Figure S1). Chalkiness is a complex trait and the reported QTLs are distributed on almost all twelve chromosomes (Additional file 1: Table S5). Furthermore, many of these QTLs were identified using primary mapping populations derived from *indica*/*japonica* crosses. Some of the QTLs were mapped using sparse RFLP markers (Ebitani et al. 2005; Terao et al. 2004; Zhou et al. 2009). We randomly selected two or more SSR markers in the QTL region to track the target QTL mapped using RFLP markers. However, 41 markers were not useable in association analysis because of low polymorphism in our population. In addition, chalkiness is very susceptible to environmental conditions such as temperature, fertilizer and humidity, which might be an important reason why only a few associations were identified using published markers (Fitzgerald et al. 2009; Yamakawa et al. 2007; Zhao and Fitzgerald, 2013; Zhou et al. 2015a). Markers on *GBSSI* and *SSIIa*, tightly linked to seven known QTLs for chalkiness, were detected to associate with DEC and PGWC (Tables 3 and 4; Additional file 1: Table S3). RMw513, a marker for *gw5*, was in strong association with DEC in all the six environments (Table 3; Additional file 1: Tables S4 and S5). RGS1, a major marker for grain size, was associated with PGWC in 3 environments. Above results indicated that chalkiness is not only affected by starch biosynthesising genes but also genes related to grain shapes.

*GBSSI* and *SSIIa* were strongly associated with AC, GC, DEC and PGWC in the present study (Tables 3 and 4; Additional file 2: Figure S1). This result might partially explain the negative correlation between AC and chalkiness although there is no direct evidence showing that AC contributes to the occurrence of endosperm chalkiness (Zhou et al. 2015a, b). RMw513 for *gw5* was strongly associated with grain shape traits and DEC. RM18751, a marker for chalkiness QTL, was highly associated with GL (Table 3; Additional file 1: Tables S5 and S7). These detected associations provided a genetic explanation of the reported correlation between grain shape and chalkiness (Adu-Kwarteng et al. 2003; Raju and Srinivas, 1991; Zhou et al. 2015a). The pleiotropic effects of these genes might be explored for the purpose of improving eating and physical quality simultaneously.

Except *GBSSI* for AC, *SSIIa* for DEC and PGWC, RGS1 for GL and LWR, other marker-trait associations only explained less than 15 % of the phenotypic variation of a trait, indicating that accumulating desirable alleles of multiple genes is necessary for achieving sizable improvement. When multiple genes are stacked together the interactions between genes will play an important role in determining trait performance of the pyramided lines (Ye and Smith, 2010). Although not studied systematically, interactions between some of the genes were present in the present study. For instance, the average DEC of Pop4 was 17.72 %, but DEC of two subsets with SSIIa-IF allele 1 and 2 in Pop4 was 12.95 and 26.67 %, respectively (Table 1; Fig. 2). In the two genotype groups of the SSIIa-IF, lines with RMw513 allele 5 had average DEC value more than 40 % lower than lines with the allele 3 of RMw513 (Fig. 2). It was obvious that combinations between SSIIa-IF and RMw513 alleles explained much more variation than a single marker, SSIIa-IF or RMw513. Another example was the interaction between PUP-4-F and SSIIa-IF on PGWC. PUP-4-F was not associated with PGWC, however the difference between PUP-4-F genotypes was 8.0 and 15.77 % for the two SSIIa-IF genotypes in Pop2 (Additional file 2: Figure S2). Therefore, further studies on interactions (epistasis) between genes are needed to provide information on designing efficient and effective pyramiding strategies for exploiting the already well characterized genes/QTLs in improving grain quality in rice.

## Conclusion

*GBSSI* and *SSIIa* were two major genes affecting AC and GC. *GBSSI* and *SSIIa* also affectted chalkiness formation in rice. *SSI*, *SSIIIb* and *ISA2* were associated with AC. *AGPS1*, *AGPS2*, *APGL2*, *BEI*, *BEIIa*, *ISA1*, *SSIIb*, *SSIIc*, *SSIIIa* and *SSIVa* did not affect any of the measured traits in the present study. Only 10 markers for 12 genes/QTLs related to grain shape or yield were found to be associated with grain shape traits. Chalkiness was affected by starch biosynthesising genes and genes related to grain shapes. The validated markers for genes/QTLs with major effects could be directly used in breeding for grain quality *via* marker-assisted selection.

## Methods

### Plant Materials and Phenotyping for Grain Quality Traits

Three hundred and nine two advanced breeding lines or released varieties were collected from many irrigated rice breeding programs in different countries to represent the diversity within the elite breeding gene pools for irrigated ecosystem (see Additional file 1: Table S1., Liang et al. 2015). Majority of the lines were from IRRI (223). The number of lines from PhilRice, CIAT, China and Vietnam were more than ten. The rest of the lines were from programs in Bangladesh, Colombia, Indonesia, Nepal, Africa Rice Center, Egypt, Pakinstan, India, Repubilic of Korea, Sri Lanka, Suriname, Turkey and so on. Field experiments were performed at the experimental farm of IRRI, Los Baños, Laguna, Philippines (14°11’N, 121°15’ E) during the 2012 dry (DS) and wet (WS) seasons with 3 nitrogen rates. The nitrogen and season combinations were designated as DS_N0_, DS_N90_, DS_N180_, WS_N0_, WS_N45_ and WS_N90_. Seeds were sown in seedling nursery and 21-day-old seedlings were transplanted with single seedling per hill. Experiments were laid out in row-column design with 2 replications. Each plot consisted of 8 × 8 hills with a spacing distance of 0.2 × 0.2 m. N in the form of urea was applied 3 times in split; basal, 14 and 42 days after transplanting with 1:1:1 ratio during whole growing season. 40 kg P ha^−1^ and 40 kg K ha^−1^ were also applied basally. Day to heading (DTH) of this population was ranged from 85.76 to 91.47 and 89.48 to 90.48 days in DS and WS respectively. DTH of more than 93 % lines were ranged from 80 to 100 days. Due to photoperiod sensitivity, insect or rat damage, some of the lines couldn’t give any production in some environments. Three hundred and eighty four lines were measured for 8 grain quality traits. Nine lines were later confirmed to be *japonica* and removed before data analysis. Finally, 375 lines were used in this study. The number of lines used for analysis for different traits varied slightly. HRY was defined as the ratio of weight of head rice after milling (Grains with length greater than or equal to ¾ of its total length) to weight of original paddy. PGWC was determined manually using more than 100 grains of polished head rice. DEC, GL and GW of polished grains were measured using a Cervitec Grain Inspector 1625 (Foss, Denmark). LWR was calculated based on the recorded grain length and width data. AC was measured by the standard iodine colorimetry method described in ISO 6647-2-2011.

### Markers and Genotyping

Five sets of markers were used in this study. The first set was 53 SSR markers distributed evenly on 12 chromosomes and used to infer structure of the population (Additional file 1: Table S2). The second set was 63 markers located in 20 starch biosynthesizing genes including AGPase (*AGPL1*, *AGPL2*, *AGPS1* and *AGPS2*), granule-bound starch synthase (*GBSSI* and *GBSSII*), starch synthase (*SSI*, *SSIIa*, *SSIIb*, *SSIIc*, *SSIIIa*, *SSIIIb*, *SSIVa* and *SSIVb*), starch branching enzyme (*BEI*, *BEIIa* and *BEIIb*) and starch debranching enzyme (*ISA1*, *ISA2* and *PUL*) (Additional file 1: Table S3). The third set included 80 markers tightly linked to 41 fine mapped QTLs/genes for grain shape, weight and panicle size (Additional file 1: Table S4). The fourth set had 76 markers closely linked to 54 published QTLs/genes related to chalkiness (Additional file 1: Table S5). The fifth set 15 markers were selected to fill in the large gaps (>5 Mb) between the above mentioned target markers (Additional file 1: Table S6).

PCR amplification was conducted in a 10 μL reaction mixture containing 50 ng template DNA, 0.5 μM of each primer, 200 μM of each dNTP, 1.5 μM MgCl_2_, 0.1 % Triton X-100 and 1 U Taq polymerase and 1.0 μL of 10× PCR buffer under the following program: 5 min at 94 °C, followed by 30 cycles of 30 s at 94 °C, 30 s at 55 °C, and 45 s at 72 °C with a final extension of 5 min at 72 °C. PCR products were separated on 6 % non-denaturing polyacrylamide gels and observed by SYBR^®^ Safe staining method.

### Statistical Analysis and Association Mapping

The population structure (Q) was detected using 53 well amplified SSR markers using STRUCTURE 2.3.4 (Falush et al. 2003; Pritchard et al. 2000). To infer the number of groups, a fully Bayesian process described by Pritchard et al. (2000) was run with different number of clusters (from 2 to 12) using admixture model. The optimum number of subpopulations was selected by lnP(d) (log posterior probability of data) after twenty independent runs of a burn-in of 5,000 interactions followed by 100,000 Markov Chain Monte Carlo (MCMC) repeats for each value of number of subgroups (Evanno et al. 2005). Finally, four was the best value of number of subpopulations. Subgroup of each line was determined by the membership probability (Pritchard et al. 2000). The membership probability of 212 lines was higher than 0.6 for one of the four subpopulations. Only 16 lines had relatively lower (0.3 ~ 0.4) posterior probabilities for all the subpopulations. The same set SSR markers was also used for calculating the relative Kinship matrix (K) using TASSEL (Bradbury et al. 2007).

All trials were separately analyzed by fitting an appropriate spatial model with rows and columns using PBTools (bbi.irri.org). The best linear unbiased estimations (BLUE) from the best-fit model were used as raw data for association analysis.

Haplotypes of 20 starch biosynthesizing genes were determined based on all tested polymorphic loci (markers) for each gene. For example, the SNPs of GBSSI-1-IF, GBSSI-3-IF and GBSSI-4-IF are G/T, A/C and C/T, respectively. Theoretically, there are 8 haplotypes of *GBSSI* based on these three loci. Rare marker alleles or haplotypes, occurring at a frequency less than 5 %, were excluded from association analysis. Finally, 147 markers plus haplotypes of 15 starch biosynthesizing genes were used. The QK model, a mixed linear model (MLM) adjusting for both population structure and genetic relatedness between genotypes, implemented by TASSEL was used for association analysis. Positive false discovery rate (*q*^FDR^; *q* value) was calculated with the R package QVALUE for multiple comparison correction using the smoother method proposed by (Storey and Tibshirani, 2003). The associations between markers and the target traits were declared as significant based on a cut-off criteria of *q*^FDR^ <0.05.

## Additional files

Additional file 1:
**Table S1.** Phenotypic data and subpopulation of tested lines in this study. **Table S2.** Fifty three SSR markers for population structure and kinship analysis. **Table S3.** Markers on starch systhesis genes. **Table S4.** Mapped QTLs or cloned genes for grain shape and yield components, marker information and references. **Table S5.** Mapped QTLs or cloned genes for chalkiness or related components, marker information and references. **Table S6.** Random selected markers for big gaps on chromosomes. **Table S7.** Number of marker-trait associations detected across 6 environments. **Table S8.** Effects of alleles of major marker associated with each trait across 6 environments. (DOCX 186 kb) Additional file 2:
**Figure S1.** Distribution of markers used in this study and identified significant marker-trait associations on chromosomes. **Figure S2.** Effects of allele combinations between SSIIa-IF and PUL-4-F on PGWC. (DOCX 122 kb)

## Abbreviations

AC: Amylose content
GC: Gel consistency
GL: Grain length
GW: Grain width
LWR: The ratio of grain length and width
DEC: Degree of endosperm chalkiness
PGWC: Percentage of grain with chalkiness
HRY: Head rice yield
DS: Dry season
WS: Wet season
