# Supplementary material for: The Usefulness of Known Genes/Qtls for Grain Quality Traits in an Indica Population of Diverse Breeding Lines Tested using Association Analysis
Source: Rice (N Y). 2015 Sep 21;8:29. doi: 10.1186/s12284-015-0064-3 (PMC4577492; doi:10.1186/s12284-015-0064-3)
Supplement: Additional file 1: — Table S1. Phenotypic data and subpopulation of tested lines in this study. Table S2. Fifty three SSR markers for population structure and kinship analysis. Table S3. Markers on starch systhesis genes. Table S4. Mapped QTLs or cloned genes for grain shape and yield components, marker information and references. Table S5. Mapped QTLs or cloned genes for chalkiness or related components, marker information and references. Table S6. Random selected markers for big gaps on chromosomes. Table S7. Number of marker-trait associations detected across 6 environments. Table S8. Effects of alleles of major marker associated with each trait across 6 environments. (DOCX 186 kb) [file 12284_2015_64_MOESM1_ESM.docx]

Supplementary table 1 Phenotypic data and subpopulation of tested lines in this study

| Fixed name | Pop | GL | GW | LWR | AC | GC | DEC | PGWC | HRY |
| --- | --- | --- | --- | --- | --- | --- | --- | --- | --- |
| IR 02A149 | 1 | 6.76 | 2.02 | 3.34 | 23.55 | 81.08 | 19.75/20.43 | 73.67/75.33 | 48.04/39.8 |
| IR 03A290 | 1 | 6.75 | 1.94 | 3.49 | 24.9 | 79.75 | 12.94/5.99 | 53.63/27.78 | 51.98/44.19 |
| IR 03N137 | 1 | 6.88 | 1.97 | 3.49 | 22.62 | 91.58 | 14.34/17.62 | 72.35/78.78 | 45.52/33.95 |
| IR 04A115 | 1 | 6.81 | 2.06 | 3.3 | 22.3 | 86.6 | 17.15/36.72 | 90.75/100 | 59.65/41.01 |
| IR 04A212 | 1 | 6.91 | 1.89 | 3.66 | 21.23 | 81.17 | 13.5/27.93 | 69.4/92.18 | 43.38/32.01 |
| IR 04A216 | 1 | 6.62 | 1.98 | 3.34 | 22.4 | 72.25 | 10.51/26.27 | 49.6/77.44 | 65.71/51.29 |
| IR 04A421^a^ | 1 | 6.84 | 2.08 | 3.28 | 24.92 | 82.83 | 24.96/31.05 | 84.93/86.9 | 53.95/36.42 |
| IR 05A272^a^ | 1 | 7.01 | 1.95 | 3.6 | 25 | 75.92 | 24.47/28.64 | 72.42/93.27 | 30.15/37.13 |
| IR 05A272 ^a^ | 1 | 7.01 | 1.91 | 3.67 | 26.25 | 76.92 | 20.08/28.8 | 82.73/98.54 | 42.54/21.58 |
| IR 05N229 | 1 | 6.74 | 2.1 | 3.21 | 25 | 63.75 | 17.94/18.32 | 82.48/58.8 | 56.32/46.1 |
| IR 05N412 | 1 | 6.74 | 2.14 | 3.15 | 22.32 | 92.08 | 15.54/35.91 | 62.43/100 | 60.14/47.26 |
| IR 05N419 | 1 | 6.66 | 2.1 | 3.18 | 25.18 | 92.58 | 26.27/39.03 | 72.58/99.7 | 54.53/48.16 |
| IR 06N146 | 1 | 6.59 | 1.98 | 3.34 | 21.47 | 78.42 | 14.11/6.38 | 61.23/33.45 | 53.9/51.12 |
| IR 06N155 ^a^ | 1 | 6.70 | 2.20 | 3.04 | 16.28 | 79.08 | 15.23/13.25 | 77.15/64.82 | 59.15/41.79 |
| IR 06N155 ^a^ | 1 | 6.59 | 2.23 | 2.96 | 17.3 | 75.42 | 10.67/23.04 | 50.7/84.29 | 60.34/46.84 |
| IR 06N209 | 1 | 6.74 | 2.03 | 3.32 | 21.7 | 81.67 | 12.1/36.8 | 56.7/100 | 63.09/40.1 |
| IR 07A137 | 1 | 6.68 | 2.07 | 3.23 | 23.07 | 71.67 | 16.87/17.76 | 71.2/67.4 | 60.9/46.65 |
| IR 07A179 | 1 | 7.08 | 1.91 | 3.72 | 18.55 | 86.08 | 5.74/8.78 | 37.09/40.32 | 58.79/42.2 |
| IR 07A234 | 1 | 6.57 | 2.13 | 3.09 | 23.95 | 67.5 | 8.46/12.28 | 42.3/59.65 | 56.63/43.11 |
| IR 07A260 | 1 | 6.81 | 2.14 | 3.18 | 20.97 | 79.92 | 36.52/46.53 | 100/100 | 44.74/46.11 |
| IR 07L167 | 1 | 6.98 | 2.14 | 3.26 | 21.67 | 89.83 | 18.82/25.67 | 89.87/96.12 | 54.35/42.26 |
| IR 07N136 | 1 | 6.59 | 2.12 | 3.1 | 22.58 | 96.8 | 13.99/24.64 | 81.95/100 | 61.68/40.24 |
| IR 08A128 | 1 | 6.43 | 2.03 | 3.17 | 25.05 | 86.25 | 9.35/15.33 | 40.8/68.67 | 57.54/44.1 |
| IR 08A131 | 1 | 6.55 | 2.12 | 3.09 | 24.47 | 96.5 | 7.21/10.72 | 32.72/48.1 | 54.96/34.89 |
| IR 08A138 | 1 | 6.72 | 2.16 | 3.12 | 21.6 | 85.58 | 26.28/37.15 | 98.52/100 | 54.74/39.1 |
| IR 08A175 | 1 | 6.65 | 2.05 | 3.24 | 22.48 | 78.83 | 16.76/26.45 | 69.12/72.95 | 59.84/45.49 |
| IR 08A176 | 1 | 6.59 | 2.16 | 3.05 | 23.67 | 92.17 | 25.6/32.56 | 77.63/100 | 56.77/48.69 |
| IR 08A191 | 1 | 6.75 | 2.07 | 3.27 | 23.17 | 88.75 | 23.47/27.8 | 84.68/82.25 | 52.7/47.15 |
| IR 08A192 | 1 | 6.77 | 2.13 | 3.18 | 24.47 | 83.67 | 7.51/13.15 | 28.8/48.66 | 67.43/54.02 |
| IR 08M119 | 1 | 6.52 | 2.12 | 3.08 | 23.82 | 85.08 | 25.57/44.75 | 89.62/100 | 51.89/38.68 |
| IR 08N113 | 1 | 6.8 | 1.99 | 3.41 | 20.88 | 78.5 | 17.7/39.62 | 90.15/98.97 | 42.63/42.3 |
| IR 08N134 | 1 | 6.65 | 2.11 | 3.15 | 23.32 | 88.8 | 9.23/38.1 | 51.08/99.6 | 60.9/50.3 |
| IR 08N138 | 1 | 6.86 | 2.1 | 3.27 | 22.52 | 94.67 | 7.46/14.95 | 33.55/78.78 | 46.21/45.88 |
| IR 08N184 | 1 | 6.85 | 2.13 | 3.22 | 20.92 | 85.75 | 37.67/37.16 | 100/100 | 37.2/23.07 |
| IR 08N215 | 1 | 6.74 | 1.96 | 3.46 | 23.12 | 82.08 | 14.93/19.4 | 47.05/82.28 | 47.45/40.33 |
| IR 09A116 | 1 | 6.81 | 1.97 | 3.47 | 22.88 | 88.42 | 27.88/32.05 | 100/99.74 | 58.65/45.3 |
| IR 09A120 | 1 | 6.83 | 1.95 | 3.51 | 22.5 | 65.75 | 4.23/9.29 | 36.5/47.55 | 51.2/48.96 |
| IR 09A136 | 1 | 6.56 | 2.14 | 3.06 | 23.15 | 88.25 | 35.02/33.73 | 97.65/99.03 | 50.07/43.72 |
| IR 09A152 | 1 | 6.77 | 2.06 | 3.3 | 23.68 | 92.17 | 29.16/29.89 | 81.28/95.48 | 49.44/39.57 |
| IR 09A192 | 1 | 6.69 | 1.98 | 3.37 | 22.82 | 86.83 | 15.32/25.41 | 71.68/90.95 | 51.96/46.32 |
| IR 09A231 | 1 | 6.8 | 2.15 | 3.17 | 24.17 | 95.83 | 23.03/15.39 | 67.62/79.8 | 59.02/58.04 |
| IR 09A235 | 1 | 6.78 | 2.13 | 3.18 | 20.35 | 71.25 | 38.86/29.5 | 100/74.08 | 47.58/44.68 |
| IR 09N142 | 1 | 6.93 | 2.06 | 3.36 | 23.77 | 92.08 | 29.9/28.62 | 99.68/96.53 | 44.62/32.4 |
| IR 09N146 | 1 | 6.75 | 2.04 | 3.31 | 24.65 | 84.83 | 23.56/30.27 | 93.2/94.8 | 55.59/39.74 |
| IR 09N190 | 1 | 6.68 | 2.08 | 3.21 | 23.55 | 92.75 | 9.43/24.16 | 43.98/86.5 | 58.84/43.37 |
| IR 09N496 | 1 | 6.87 | 2.04 | 3.37 | 22.08 | 82.25 | 22.75/30.17 | 96.13/99.77 | 38.18/36.66 |
| IR 09N509 | 1 | 6.79 | 1.88 | 3.62 | 24.43 | 83 | 10.42/18.35 | 52.72/72.63 | 56.23/36.59 |
| IR 09N514 | 1 | 6.78 | 1.79 | 3.79 | 22.22 | 81.33 | 16.21/26.59 | 61.52/96.32 | 46.6/23.56 |
| IR 09N527 | 1 | 6.85 | 1.9 | 3.62 | 22.52 | 74.83 | 21.99/25.88 | 76.43/79.17 | 53.48/44.84 |
| IR 09N528 | 1 | 6.77 | 1.89 | 3.58 | 22.43 | 76.92 | 21.06/23.58 | 73.43/92.33 | 60.33/37.63 |
| IR 09N533 | 1 | 6.56 | 2.1 | 3.12 | 25.92 | 85 | 31.62/35.73 | 96.83/94.62 | 57.81/46.81 |
| IR 10A108 | 1 | 6.71 | 2.06 | 3.25 | 19.47 | 86.67 | 10.3/18.48 | 41.83/54.53 | 58.16/36.11 |
| IR 10A125 | 1 | 6.77 | 2.03 | 3.34 | 21.98 | 86.92 | 6.17/14.61 | 28.88/73.13 | 65.5/43.66 |
| IR 10A133 | 1 | 6.75 | 2 | 3.4 | 24.05 | 86.33 | 17.29/21.4 | 75.95/85.03 | 52.36/37.09 |
| IR 10A142 | 1 | 6.9 | 1.95 | 3.54 | 22.27 | 92.75 | 12.9/17.96 | 55.35/66.45 | 27.26/21.97 |
| IR 10A144 | 1 | 6.83 | 2.09 | 3.27 | 22.43 | 86.17 | 14.11/21.13 | 64.47/78.5 | 34.46/39.48 |
| IR 10F203 | 1 | 6.65 | 1.99 | 3.35 | 26.58 | 99.58 | 4.42/4.38 | 23.67/21.18 | 61.47/53.56 |
| IR 10F328 | 1 | 6.82 | 1.92 | 3.55 | 22.13 | 80.42 | 19.17/21.99 | 87.13/98.15 | 46.39/38.05 |
| IR 10F371 | 1 | 6.62 | 1.93 | 3.44 | 22.4 | 85.75 | 17.92/16.58 | 73.97/77 | 60.24/55.04 |
| IR 10F379 | 1 | 6.78 | 1.82 | 3.72 | 22.42 | 92.08 | 6.66/7.14 | 33.77/27.7 | 56.47/49.84 |
| IR 10N108 | 1 | 6.85 | 2.1 | 3.26 | 21.92 | 89.08 | 21.84/34.15 | 82.57/94.03 | 48.71/39.8 |
| IR 10N118 | 1 | 6.67 | 2.09 | 3.2 | 22.47 | 89.17 | 27.57/33.88 | 90.03/100 | 54.98/44.7 |
| IR 10N184 | 1 | 6.84 | 2.08 | 3.3 | 21.67 | 94.17 | 30.58/45.2 | 99.33/100 | 54.02/45.62 |
| IR 10N186 | 1 | 6.69 | 2.09 | 3.21 | 21.55 | 89.75 | 37.72/50.33 | 100/100 | 60.42/38.11 |
| IR 10T113 | 1 | 6.74 | 1.97 | 3.42 | 22.98 | 90.5 | 27.34/29.5 | 86.98/98.35 | 35.2/29.15 |
| IR 71146-97-1-2-1-3^a^ | 1 | 6.76 | 2.08 | 3.25 | 18.03 | 81.17 | 19.68/28.44 | 88.62/93.23 | 42.38/41.05 |
| IR 71146-97-1-2-1-3^a^ | 1 | 6.81 | 2.09 | 3.26 | 18.58 | 66.92 | 20.7/36.15 | 91.17/100 | 53.96/41.94 |
| IR 72892-77-2-2-2 | 1 | 7.07 | 1.97 | 3.6 | 23.18 | 92 | 39.95/33.9 | 97.12/100 | 40.67/38.43 |
| IR 77186-148-3-4-3 | 1 | 6.83 | 2.06 | 3.33 | 21.95 | 97.08 | 16.19/24.04 | 89.12/97.85 | 46.32/42.44 |
| IR 78545-49-2-2-2 | 1 | 6.74 | 2.01 | 3.35 | 21.12 | 77.33 | 13.24/14.59 | 55.05/79.05 | 62.52/48.06 |
| NSIC Rc212 | 1 | 6.78 | 1.86 | 3.64 | 22.53 | 90.58 | 19.68/22.33 | 72.15/95.85 | 53.96/42.27 |
| PEH-KUH-TSAO-TU | 1 | 6.69 | 2.05 | 3.27 | 22.2 | 94.42 | 23.21/38.87 | 95.7/100 | 57.8/41.45 |
| PR37951-3B-37-1 | 1 | 6.85 | 2.04 | 3.36 | 25.38 | 69.42 | 10.89/18.03 | 50.95/75.83 | 55.86/33.28 |
| PR37952-B-4-1-3 | 1 | 7.05 | 1.77 | 3.98 | 24.17 | 86.17 | 3.27/13.7 | 16.75/69.48 | 53.11/27.35 |
| PR40083-1B-3-2 | 1 | 6.48 | 2.11 | 3.07 | 23.98 | 82.17 | 13.06/20.41 | 41.06/77.02 | 59.81/45.78 |
| HHZ5-SAL10-DT1-DT1 | 1 | 6.98 | 2.15 | 3.25 | 26.55 | 64.67 | 8.45/22.9 | 46.05/95.04 | 54.49/37.78 |
| PSBRC82 | 1 | 6.73 | 2.04 | 3.3 | 21.58 | 90.25 | 18.82/25.76 | 91.28/92.88 | 61.9/51.25 |
| PSBRC82-SUB1 | 1 | 6.74 | 1.98 | 3.41 | 21.72 | 94.58 | 9.94/30.48 | 51.5/99.57 | 57.99/47.05 |
| B11143D-MR-1-PN-3-MR-3-SI-2-3-PN-1 | 2 | 6.46 | 2.18 | 2.96 | 22.77 | 53.08 | 45.01/38.23 | 96.97/100 | 59.33/41.23 |
| C 3419-10-1-2 (PSB RC98) | 2 | 6.71 | 2.11 | 3.2 | 25.73 | 89.17 | 6.73/9.39 | 29.65/47.05 | 60.94/42.4 |
| C7546WH-2-2-1-1-4-2-1-2-1-1-2 | 2 | 6.76 | 2.04 | 3.34 | 23.05 | 76.42 | 6.71/8.76 | 25.75/37.6 | 57.97/40 |
| Ciherang | 2 | 6.73 | 2.08 | 3.24 | 22.62 | 74.58 | 11.5/17.62 | 56.73/67.1 | 56.92/36.34 |
| CT 15672-12-1-5-2-4-M | 2 | 6.87 | 2.1 | 3.28 | 26.6 | 83.5 | 25.58/30.51 | 84.73/98.12 | 28.61/26.49 |
| CT 18160-3-2-2-3-1-M | 2 | 6.87 | 2.14 | 3.21 | 26.8 | 57.17 | 6.27/12.4 | 25.05/55.7 | 52.34/45.17 |
| GZ 7712-BC-H-6-20 | 2 | 5.21 | 2.73 | 1.91 | 14.77 | 77.83 | 27.08/10.73 | 62.1/33.22 | 53.54/61.28 |
| HHZ 1-Y4-Y1 | 2 | 6.52 | 1.99 | 3.28 | 16.52 | 78.25 | 0.7/4.74 | 6.67/20.58 | 61.8/51.6 |
| HHZ-12-Y4-Y3-1 | 2 | 6.43 | 2.16 | 2.97 | 26.97 | 69.75 | 15.69/3.44 | 40.48/19.63 | 54.47/48.14 |
| HUANGHUAZHAN | 2 | 6.49 | 2.07 | 3.13 | 22.92 | 87.58 | 33.86/40.96 | 94.48/100 | 36.24/37.81 |
| IR 01A160 | 2 | 6.53 | 1.98 | 3.3 | 23.58 | 78.08 | 26.17/39.16 | 78.29/100 | 58.83/37.85 |
| IR 02A127 | 2 | 6.57 | 2.06 | 3.18 | 22.35 | 77 | 35.8/31.93 | 98.33/96.7 | 41.23/49.47 |
| IR 03A159 | 2 | 6.7 | 1.99 | 3.37 | 26.9 | 90.67 | 9.67/20.47 | 54.4/79.67 | 50.8/33.84 |
| IR 03A477 | 2 | 6.71 | 2 | 3.36 | 26.25 | 92.17 | 8.78/13.16 | 45.5/64.12 | 61.31/26.92 |
| IR 03A500 | 2 | 6.63 | 2.09 | 3.17 | 22.45 | 88.5 | 24.17/38.15 | 87.97/100 | 56.96/43.05 |
| IR 03A550 | 2 | 6.82 | 2.19 | 3.11 | 18.1 | 84.33 | 9.5/7.58 | 56.57/37.52 | 57.94/55.72 |
| IR 04A409 | 2 | 6.76 | 2.1 | 3.22 | 25.68 | 77.67 | 13.31/13.41 | 65.17/69 | 56.22/53.86 |
| IR 04A428 | 2 | 7 | 2.08 | 3.36 | 24.28 | 100 | 35.59/41.11 | 99.43/100 | 50.31/43.79 |
| IR 05A260 | 2 | 6.8 | 2.08 | 3.29 | 22.27 | 90.58 | 24.06/28.03 | 83.72/94.72 | 44.1/32.11 |
| IR 05A278 | 2 | 6.98 | 2.06 | 3.39 | 23.87 | 77.17 | 5.59/18.24 | 25.78/69.95 | 59.54/44.98 |
| IR 05N170 | 2 | 6.86 | 2.07 | 3.32 | 26.08 | 78.75 | 21.16/33.07 | 64.27/96.2 | 48.91/43.28 |
| IR 05N229 | 2 | 6.71 | 2.09 | 3.21 | 23.17 | 77.08 | 26.54/25.39 | 73.6/95.03 | 55.56/45.73 |
| IR 05N386 | 2 | 6.86 | 2.14 | 3.2 | 27.98 | 80.5 | 13.82/31.84 | 56.32/99.3 | 44.69/32.46 |
| IR 06A144 | 2 | 6.54 | 2.08 | 3.14 | 23.22 | 84.83 | 41.71/37.71 | 100/100 | 27.54/44.16 |
| IR 06A145^a^ | 2 | 6.63 | 2.04 | 3.25 | 22.76 | 80.2 | 50.77/34.43 | 100/100 | 38.38/40.66 |
| IR 06A145^a^ | 2 | 6.52 | 2.12 | 3.08 | 18.7 | 75.3 | 26.98/35.86 | 100/100 | 50.23/36.05 |
| IR 06A152 | 2 | 6.5 | 1.89 | 3.43 | 21.13 | 126 | 6.31/10.5 | 35.12/55.6 | 51.17/35.28 |
| IR 06A181 | 2 | 6.58 | 2.02 | 3.26 | 23.23 | 85.38 | 34.3/33.92 | 100/97.59 | 55.87/42.8 |
| IR 06M139 | 2 | 6.88 | 2.08 | 3.3 | 14.97 | 78.58 | 8.71/19.8 | 40.77/76.43 | 51.86/50.69 |
| IR 06M141 | 2 | 6.82 | 2.11 | 3.23 | 18.25 | 82.25 | 1.38/6.03 | 13.62/30.08 | 62.26/49.47 |
| IR 06M142 | 2 | 6.98 | 2.13 | 3.29 | 23.07 | 76.58 | 34.4/42.67 | 96.78/99.62 | 49.7/35.91 |
| IR 06M143 | 2 | 6.94 | 2.09 | 3.33 | 18.97 | 68.25 | 3.49/3.09 | 16.33/13.12 | 51.04/41.67 |
| IR 06M150 | 2 | 6.81 | 2.07 | 3.29 | 19.8 | 73.67 | 2.87/12.33 | 13.45/37.78 | 60.42/45.72 |
| IR 06N119^a^ | 2 | 6.63 | 1.94 | 3.42 | 23.32 | 77.42 | 16.81/27.51 | 68.27/95.43 | 47.91/40.4 |
| IR 06N119^a^ | 2 | 6.65 | 2.04 | 3.26 | 23.37 | 88.08 | 14.52/16.26 | 61.06/80.97 | 59.29/46.57 |
| IR 06N233 | 2 | 6.88 | 2.01 | 3.42 | 26.96 | 60.2 | 1.01/25.28 | 9.88/83.45 | 44.65/47.33 |
| IR 07A107 | 2 | 6.76 | 2.05 | 3.3 | 23 | 91.17 | 21.89/18.19 | 82.28/73.97 | 54.14/44.07 |
| IR 07A166 | 2 | 6.97 | 2.05 | 3.41 | 26.42 | 76.67 | 21.86/23.67 | 94.72/92.15 | 40.86/40.07 |
| IR 07A253 | 2 | 6.57 | 2.07 | 3.17 | 24.2 | 97.25 | 17.63/30.73 | 75.38/90.7 | 55.22/45.12 |
| IR 07N123 | 2 | 6.86 | 1.99 | 3.45 | 21.4 | 86.92 | 21.71/19.11 | 71.6/82.43 | 46.31/39.84 |
| IR 07T102 | 2 | 6.42 | 1.9 | 3.39 | 24.53 | 69.67 | 5.83/8.26 | 25.8/46.72 | 61.75/54.24 |
| IR 07T104 | 2 | 6.84 | 2.07 | 3.31 | 18.8 | 63.75 | 7.91/4.93 | 32.57/25.83 | 59.53/56.01 |
| IR 08N158 | 2 | 6.92 | 1.85 | 3.74 | 25.23 | 72 | 14.15/19.95 | 73.77/81.62 | 38.57/37.55 |
| IR 09A104 | 2 | 6.53 | 2.01 | 3.25 | 22.23 | 77.5 | 8.8/21.29 | 42.27/80.07 | 60.68/54.52 |
| IR 09A128 | 2 | 6.62 | 2.06 | 3.22 | 23.48 | 95 | 25.15/22.76 | 95.16/90.98 | 58.65/45.65 |
| IR 09A172 | 2 | 6.71 | 2.03 | 3.3 | 23.93 | 91.25 | 33.2/31.63 | 97.92/100 | 48.54/44.95 |
| IR 09A181 | 2 | 6.29 | 2.18 | 2.88 | 23.76 | 91.7 | 19.77/28.81 | 87.52/97.55 | 62.64/45.4 |
| IR 09A220 | 2 | 6.85 | 1.97 | 3.49 | 23.38 | 83.25 | 18.79/12.96 | 87.07/61.62 | 39.2/44.37 |
| IR 09A229 | 2 | 6.79 | 2.19 | 3.1 | 22.8 | 82.9 | 25.4/41.65 | 100/100 | 55.35/43.44 |
| IR 09M106 | 2 | 6.7 | 2.13 | 3.16 | 22.43 | 89.42 | 39.71/37.15 | 100/96.8 | 57.75/44.23 |
| IR 09N272 | 2 | 6.93 | 2.14 | 3.24 | 25 | 66.25 | 23.26/27.23 | 95.23/81.27 | 54.92/49.25 |
| IR 09N495 | 2 | 6.78 | 2.09 | 3.24 | 22.08 | 89.2 | 12.03/23.39 | 59.18/82.63 | 58.65/47.7 |
| IR 09N499 | 2 | 6.62 | 2.05 | 3.24 | 24.8 | 96 | 10.77/21.38 | 60.43/90.62 | 60.82/49.47 |
| IR 09N500 | 2 | 6.6 | 2 | 3.3 | 26.28 | 98.33 | 9.37/17.09 | 47.73/70.27 | 55.75/54.44 |
| IR 09N503 | 2 | 6.64 | 1.75 | 3.79 | 27.75 | 100 | 2.69/2.61 | 15.2/12.33 | 57.03/43.53 |
| IR 09N508 | 2 | 6.6 | 2.07 | 3.21 | 19.22 | 78.6 | 9.21/20.12 | 53.1/81.6 | 56.48/44.19 |
| IR 09N522 | 2 | 6.7 | 2.08 | 3.22 | 21.48 | 79.58 | 8.36/31.99 | 45.1/99.64 | 58.91/47.52 |
| IR 09N530 | 2 | 6.65 | 2.11 | 3.16 | 20.67 | 73.17 | 33.56/40.18 | 98.18/100 | 58.87/44.98 |
| IR 09N540 | 2 | 6.63 | 2.13 | 3.12 | 22.22 | 93.83 | 23.21/26.91 | 91.1/99.37 | 53.74/46.67 |
| IR 10A128 | 2 | 6.54 | 1.92 | 3.4 | 21.9 | 83.75 | 7.81/24.25 | 41.15/56.1 | 54/36.08 |
| IR 10A152 | 2 | 6.44 | 2.19 | 2.95 | 22.88 | 88.5 | 40.93/52.48 | 100/100 | 43.21/35.95 |
| IR 10A155 | 2 | 6.69 | 2.15 | 3.12 | 25.63 | 72.67 | 14.31/19.24 | 68.72/82.28 | 56.13/41.48 |
| IR 10M120 | 2 | 6.57 | 2.02 | 3.25 | 22.13 | 83.33 | 14.79/29.48 | 68.28/85.27 | 58.93/42.84 |
| IR 10M122 | 2 | 6.37 | 2.01 | 3.17 | 19.92 | 74.1 | 9.82/15.19 | 52/70.25 | 60.92/47.28 |
| IR 10M123 | 2 | 6.4 | 2.02 | 3.18 | 20.27 | 82.92 | 15.72/23.72 | 75.53/90.52 | 57.96/41.74 |
| IR 10N134 | 2 | 6.7 | 2.17 | 3.09 | 22.2 | 82.92 | 26.49/20.15 | 98.78/82.7 | 61.52/47.21 |
| IR 10N225 | 2 | 6.77 | 2.04 | 3.32 | 21.75 | 76.25 | 10.95/10.39 | 53.5/52.4 | 53.04/39.47 |
| IR 10N230 | 2 | 6.91 | 2.15 | 3.22 | 22.37 | 93.42 | 34.87/42.91 | 100/100 | 54.01/45.37 |
| IR 10N251 | 2 | 6.62 | 1.99 | 3.32 | 20.23 | 91.58 | 8.98/20.53 | 48.42/66.73 | 57.83/42.62 |
| IR 36 | 2 | 6.55 | 1.99 | 3.28 | 25.83 | 75.5 | 14.06/27.24 | 56.73/96.85 | 54.18/21.51 |
| IR 38 | 2 | 6.73 | 2.05 | 3.29 | 23.1 | 74.92 | 18.14/24.68 | 76.83/87.2 | 47.27/44.65 |
| IR 44 | 2 | 6.65 | 2.14 | 3.11 | 27.17 | 47.83 | 8.15/10.54 | 43.87/48.98 | 62.01/49.46 |
| IR 50 | 2 | 6.28 | 1.93 | 3.26 | 27.62 | 83.58 | 6.51/5.57 | 37.27/26.13 | 60.34/42.94 |
| IR 56 | 2 | 6.66 | 2.03 | 3.28 | 26.46 | 51.1 | 5.18/6.73 | 39.7/31.28 | 59.33/44.67 |
| IR 58 | 2 | 6.09 | 2.16 | 2.82 | 25.47 | 34.5 | 13.05/12.16 | 67.87/50.13 | 60.77/53.76 |
| IR 60 | 2 | 6.37 | 1.96 | 3.26 | 24.88 | 70.08 | 4/4.76 | 19.6/18.93 | 56.17/46.96 |
| IR 65483-111-5-9-2-11 | 2 | 6.42 | 2.11 | 3.04 | 27.38 | 39.17 | 10.09/10.14 | 60.82/60.78 | 49.83/44.97 |
| IR 65483-118-25-31-7-1 | 2 | 6.64 | 1.94 | 3.42 | 25.63 | 61.08 | 2.02/14.19 | 10.5/44.97 | 58.08/32.3 |
| IR 66 | 2 | 6.45 | 1.96 | 3.3 | 26.55 | 98.92 | 4.4/10.37 | 24.38/48.93 | 61.01/47.95 |
| IR 68 | 2 | 7.09 | 2.07 | 3.42 | 27.4 | 59.42 | 17.63/28.78 | 71.43/77.57 | 45.2/42.27 |
| IR 71033-121-15 | 2 | 6.99 | 2.12 | 3.29 | 22.47 | 94.42 | 33.79/40.81 | 100/100 | 40.3/27.96 |
| IR 71033-4-1-127-B | 2 | 6.56 | 1.98 | 3.32 | 21.3 | 75.58 | 13.65/9.67 | 62.72/40.3 | 61.82/48.39 |
| IR 72 | 2 | 6.52 | 2.12 | 3.08 | 26.68 | 93.25 | 38.29/33.08 | 99.1/99.66 | 43.96/26.03 |
| IR 73004-3-1-2-1 | 2 | 6.42 | 2.17 | 2.95 | 21.83 | 89.08 | 35.43/37.81 | 100/98.25 | 59.37/41.35 |
| IR 74095-AC 45 | 2 | 5.82 | 2.14 | 2.73 | 20.53 | 70.17 | 14.01/35.09 | 67.07/85.12 | 57.57/46.9 |
| IR 74099-AC 7 | 2 | 6.1 | 2.3 | 2.66 | 25.27 | 90.17 | 19.99/21.36 | 86.73/77.75 | 52.47/46.32 |
| IR 78222-20-7-148-2-B | 2 | 6.51 | 2.2 | 2.96 | 21.02 | 86.42 | 27.74/36.28 | 88.8/96.6 | 35.45/32.71 |
| IR 78555-68-3-3-3 | 2 | 6.69 | 2.1 | 3.18 | 26.75 | 75.83 | 30.14/30.01 | 99.59/100 | 54.97/39.72 |
| IR 79195-42-1-3-1 | 2 | 7 | 2.04 | 3.43 | 25.53 | 81.5 | 12.01/13.28 | 58.77/53.93 | 55.45/44.52 |
| IR 79233-1-2-1-2 | 2 | 6.32 | 2.3 | 2.8 | 24.07 | 74.33 | 24.22/41.01 | 75.18/100 | 57.4/47.97 |
| IR 80340-12-B-B-1-2-B-B | 2 | 6.65 | 2.14 | 3.12 | 26.17 | 91.83 | 31.83/35.94 | 85.47/100 | 51.37/46.98 |
| IR 81352-65-2-1-3-3 | 2 | 6.77 | 2.14 | 3.17 | 24.25 | 88.58 | 17.3/15.51 | 78.87/80.18 | 50.55/46.93 |
| IRBB56 | 2 | 6.84 | 2.06 | 3.31 | 13.72 | 71 | 3.78/4.34 | 16.38/22.63 | 61.71/44.44 |
| IRRI 139 | 2 | 5.42 | 1.87 | 2.9 | 20.3 | 60.17 | 16.81/21.97 | 69.42/77.61 | 61.34/41.14 |
| MALA | 2 | 6.36 | 2.05 | 3.11 | 27.53 | 76.75 | 4.96/8.66 | 24.96/39.22 | 60.4/42.38 |
| NR 11 | 2 | 6.48 | 2.18 | 2.98 | 26.88 | 93.58 | 17.5/19.41 | 84.65/91.35 | 63.95/49.63 |
| NSIC Rc 152 | 2 | 6.82 | 1.97 | 3.47 | 27.67 | 56.58 | 1.13/2.36 | 6.87/10.88 | 64.56/59.72 |
| NSIC Rc 214 | 2 | 6.76 | 2.09 | 3.24 | 22.93 | 70.42 | 13.07/7.72 | 58.32/35.23 | 60.91/52.81 |
| OM 6378 | 2 | 6.89 | 2.01 | 3.42 | 22.05 | 88.58 | 13.34/17.74 | 64/68.95 | 53.06/42.06 |
| OM 6610 | 2 | 6.64 | 2.16 | 3.08 | 15.67 | 83.5 | 18.04/14.81 | 86.9/70.98 | 58.72/43.9 |
| PR34641-2B-15-1-1-1 | 2 | 6.25 | 2.2 | 2.84 | 4.07 | 91.75 | NA | NA | 65.32/51.27 |
| PR34859-B-4-1-1-2-1 | 2 | 6.83 | 2.09 | 3.27 | 16.25 | 77 | 1.87/1.51 | 10.17/10.63 | 58.9/43.27 |
| PR35786-B-3-1-3-1-1-3 | 2 | 6.71 | 2.02 | 3.32 | 25.13 | 76 | 7.67/16.34 | 36.02/84.9 | 58.12/51.82 |
| PR35786-B-3-3-2-1-1 | 2 | 6.83 | 2.08 | 3.28 | 25.27 | 80.17 | 18.23/13.29 | 54.23/61.52 | 52.64/52.94 |
| PR37152-2-2-4-1-1-1 | 2 | 6.84 | 2.18 | 3.15 | 24.78 | 82.8 | 4.85/7.93 | 21.65/34.55 | 56.36/46.66 |
| PR37246-2-3-2-1-1-2-2 | 2 | 6.62 | 2.23 | 3.01 | 25.05 | 63.67 | 43.11/28.39 | 96.98/87.08 | 55.65/26.7 |
| PR37264-1-4-1-1-3 | 2 | 6.54 | 2.06 | 3.18 | 26.63 | 99.67 | 14.16/28.23 | 59.52/100 | 51.85/41.24 |
| PR37704-2B-6-1-2-1-1 | 2 | 6.71 | 2.08 | 3.23 | 26.4 | 94.25 | 5.27/32.28 | 22.25/98.93 | 58.57/22.68 |
| PR37921-B-3-4-2-1-2 | 2 | 6.9 | 2.1 | 3.29 | 21.63 | 83.92 | 9.82/14.95 | 32.65/73 | 51.9/47.53 |
| PR37952-B-1-1-2 | 2 | 6.85 | 2.1 | 3.26 | 23.28 | 63.25 | 3.16/6.7 | 17.05/24.57 | 49.14/48.39 |
| PSBRC30 | 2 | 6.61 | 2.14 | 3.09 | 21.18 | 80.67 | 26.62/35.48 | 98.68/96.6 | 56.75/36.09 |
| PSBRC96 | 2 | 6.85 | 2.03 | 3.38 | 26.22 | 55.83 | 22.55/24.92 | 90.97/78.82 | 47.76/47.82 |
| RADHA 4 | 2 | 5.76 | 2.45 | 2.36 | 27.07 | 74.67 | 30.56/36.67 | 100/100 | 63.29/54.45 |
| B12743-MR-18-2-3 | 3 | 6.79 | 2.16 | 3.15 | 21.07 | 75.5 | 43.78/38.57 | 100/100 | 30.68/24.52 |
| BP 10618F-BB8-13-BB8 | 3 | 6.61 | 2.11 | 3.14 | 22.73 | 86.58 | 19.85/35.75 | 72.82/97.92 | 60.79/43.87 |
| BP 10620F-BB8-15-BB4 | 3 | 6.73 | 2.1 | 3.2 | 15.13 | 82.42 | 4.51/5.28 | 25.97/26.5 | 59.43/55.97 |
| BP 1356-1G-KN-4 | 3 | 6.87 | 2.39 | 2.87 | 20.37 | 82.67 | 49.47/37.46 | 100/100 | 41.91/25.81 |
| BP1976B-2-3-7-TB-1-1 | 3 | 6.29 | 2.2 | 2.86 | 22.13 | 91.33 | 10.49/22.99 | 57.93/92.35 | 59.06/48.79 |
| HHZ 12-Y 4-DT 1-Y 1 | 3 | 6.36 | 1.99 | 3.2 | 26.27 | 40.5 | 2.26/1.66 | 10.22/9.48 | 57.32/51.99 |
| IR 01A122 | 3 | 6.5 | 2.08 | 3.12 | 23.08 | 97.5 | 21.77/24.08 | 95.25/89.37 | 57.57/47.55 |
| IR 02A201 | 3 | 6.46 | 1.99 | 3.25 | 23.28 | 85.58 | 7.69/6.12 | 47.08/34.8 | 66.38/58.68 |
| IR 02A496 | 3 | 6.93 | 2.05 | 3.39 | 24.03 | 95.17 | 18.14/36.67 | 65.6/95.57 | 62.07/36.39 |
| IR 03A262 | 3 | 6.87 | 2.11 | 3.26 | 22.62 | 84.67 | 35.44/36.74 | 89.78/100 | 48.04/46.74 |
| IR 04A216 | 3 | 6.79 | 2.05 | 3.32 | 20.92 | 81.58 | 5.26/9.72 | 26.43/49.43 | 54.35/44.53 |
| IR 04A285 | 3 | 6.86 | 1.81 | 3.8 | 22.98 | 77.67 | 9.94/10.82 | 52.28/54.1 | 54.17/35.9 |
| IR 04A395 | 3 | 6.76 | 2.05 | 3.3 | 23.55 | 90.5 | 19.94/20.19 | 69.77/87.35 | 57.12/36.52 |
| IR 04N106 | 3 | 6.89 | 2.08 | 3.32 | 25.22 | 83.5 | 16.26/18.07 | 80.93/79.65 | 49.51/32.61 |
| IR 06M145 | 3 | 6.7 | 2.21 | 3.04 | 24.17 | 79.42 | 30.42/36.42 | 95.67/100 | 44.85/39.62 |
| IR 06N147 | 3 | 6.41 | 2.1 | 3.06 | 26.17 | 60.92 | 24.06/24.69 | 93.93/100 | 62.02/52.06 |
| IR 06N234 | 3 | 6.81 | 2.01 | 3.4 | 22.55 | 82.92 | 13.31/23 | 69.52/78.43 | 58.95/37.86 |
| IR 07A183 | 3 | 6.66 | 2.12 | 3.15 | 22.35 | 96.58 | 17.85/18.97 | 81.05/77.78 | 60.37/39.55 |
| IR 07A250 | 3 | 6.92 | 2.17 | 3.19 | 22.53 | 95.58 | 35.02/43.42 | 99.1/100 | 39.92/36.57 |
| IR 07A253 | 3 | 6.66 | 2.02 | 3.3 | 23.13 | 91.17 | 14.15/31.8 | 68/100 | 64.76/38.33 |
| IR 08A104 | 3 | 6.77 | 2.05 | 3.31 | 20.13 | 83.25 | 27.32/33.77 | 92.8/100 | 54.7/50.99 |
| IR 08A141 | 3 | 6.63 | 2.12 | 3.14 | 24.3 | 92.92 | 20.21/28.53 | 77.42/96.26 | 47.05/54.6 |
| IR 08M113 | 3 | 6.61 | 2.09 | 3.22 | 25.87 | 82.17 | 18.66/43.01 | 65.28/100 | 60.15/43.54 |
| IR 08N136 | 3 | 6.6 | 2.08 | 3.18 | 22.88 | 75.42 | 12.03/41.68 | 59.92/100 | 53.26/47.05 |
| IR 08N195 | 3 | 6.89 | 2.08 | 3.32 | 22.6 | 94.92 | 21.34/38.11 | 93.23/99.57 | 63.43/49.16 |
| IR 09A130 | 3 | 6.65 | 1.96 | 3.39 | 22.2 | 77.83 | 6.77/10.67 | 33.1/45.55 | 63.85/50.9 |
| IR 09A131 | 3 | 6.85 | 2.04 | 3.36 | 23.13 | 85.92 | 18.31/27.7 | 70.38/96.15 | 58.59/40.54 |
| IR 09N247 | 3 | 6.74 | 2.05 | 3.28 | 22.55 | 80.75 | 9.57/7.37 | 45.63/29.25 | 54.3/45.64 |
| IR 10A110 | 3 | 6.96 | 1.95 | 3.57 | 23.1 | 76.08 | 11.15/21.11 | 60.33/73.82 | 33.61/33.22 |
| IR 10A127 | 3 | 6.79 | 2.06 | 3.3 | 23.53 | 93.67 | 11.24/25.91 | 50.82/78.88 | 54.51/39.24 |
| IR 10A134 | 3 | 6.77 | 2 | 3.4 | 22.68 | 83.17 | 11.83/17.61 | 62.55/77.76 | 47.75/50.75 |
| IR 10A135 | 3 | 6.85 | 2.08 | 3.3 | 23.28 | 95 | 36.46/45.66 | 99.92/100 | 42.82/19.08 |
| IR 10F202 | 3 | 6.87 | 2.06 | 3.35 | 25.14 | 76.7 | 18.98/25.53 | 58.65/80.1 | 57.65/45.7 |
| IR 10F339 | 3 | 6.73 | 1.97 | 3.42 | 22.15 | 81.67 | 9.78/12.85 | 47.85/61.88 | 56.88/38.66 |
| IR 10F388 | 3 | 6.62 | 2.1 | 3.16 | 24.27 | 85 | 46.64/27.95 | 100/76.72 | 47.56/43.5 |
| IR 10N211 | 3 | 6.67 | 2.03 | 3.28 | 23.32 | 90.42 | 26.3/29.86 | 74.1/100 | 54.02/47.65 |
| IR 10N226 | 3 | 6.42 | 2.07 | 3.1 | 22.66 | 92.5 | 13.04/46.19 | 55.95/100 | 63.66/47.09 |
| IR 10N237 | 3 | 6.6 | 1.99 | 3.32 | 23.4 | 88.67 | 14.13/18.16 | 58.6/81.72 | 55.1/36.3 |
| IR 10N272 | 3 | 6.79 | 2.15 | 3.16 | 21.98 | 93.75 | 34.24/35.1 | 100/100 | 61.34/50.24 |
| IR 28 | 3 | 6.51 | 2.12 | 3.1 | 23.17 | 67.08 | 16.54/27.33 | 88.62/96.18 | 60.12/47.17 |
| IR 64 | 3 | 6.81 | 2.07 | 3.3 | 22.65 | 95.83 | 15.82/22.89 | 72.67/90.7 | 63.87/49.97 |
| IR 78554-145-1-3-2 | 3 | 6.65 | 2.1 | 3.17 | 22.15 | 81.75 | 21.84/20.03 | 96.55/89.73 | 51.77/38.48 |
| IR 79195-42-1-3-1 | 3 | 6.82 | 2.07 | 3.29 | 26.82 | 99.58 | 6.55/16.75 | 34.97/80.33 | 63.72/49.96 |
| IR 82870-57 | 3 | 6.7 | 2.13 | 3.16 | 23.45 | 77 | 32.66/40.91 | 100/100 | 59.95/34.07 |
| IRGA 370-38-1-1F-C4-2 | 3 | 6.74 | 2 | 3.37 | 26.83 | 52 | 2.95/6.71 | 15.65/29.92 | 52.9/52.27 |
| MATATAG2 | 3 | 6.63 | 2.06 | 3.23 | 23.08 | 88.67 | 23.93/16.48 | 96.33/85.53 | 60.28/43.94 |
| MTS1-1071 | 3 | 6.68 | 2.01 | 3.33 | 23.2 | 83.58 | 24.42/21.15 | 95.43/83.68 | 58.61/49.59 |
| MTS-1681 | 3 | 6.71 | 2.15 | 3.13 | 24.33 | 86.92 | 12.27/25.21 | 68.1/98.98 | 59.32/52.05 |
| NERICA-L-35 | 3 | 6.83 | 2.04 | 3.36 | 26.67 | 78.83 | 43.53/28.18 | 100/73.23 | 52.41/43.36 |
| NSIC Rc122 | 3 | 6.71 | 2.07 | 3.25 | 23.23 | 88.5 | 18.29/25.98 | 87.03/96.72 | 55.54/41.13 |
| OM 5628 | 3 | 6.7 | 2.08 | 3.22 | 22.37 | 95.25 | 18.33/33.47 | 79.1/100 | 49.8/42.41 |
| OM 5629 | 3 | 6.65 | 2.23 | 2.98 | 25.75 | 92.08 | 18.87/25.94 | 80.15/98.98 | 50/46.87 |
| OM 6073 | 3 | 6.87 | 2.08 | 3.3 | 18.42 | 76.25 | 10.35/20 | 61.37/91.92 | 46.95/36.81 |
| OMCS 2000 | 3 | 6.68 | 2.02 | 3.31 | 23.65 | 90.92 | 7.12/11.88 | 39.13/65.33 | 60.72/51.48 |
| OMCS 2009 | 3 | 6.81 | 2.08 | 3.27 | 22.73 | 94 | 25.86/40.13 | 83.72/100 | 57.09/37.71 |
| PK 7909-3-1-2-2 | 3 | 6.82 | 2.04 | 3.35 | 22.43 | 89.42 | 24.86/22.64 | 100/99.05 | 63.25/49.31 |
| PR30245-10-414 | 3 | 6.66 | 2.13 | 3.12 | 25.12 | 83.5 | 9.77/13.38 | 54.58/77.05 | 65.12/56.08 |
| PR35251-2B-5-5-3-1-1 | 3 | 6.64 | 2.08 | 3.19 | 21.08 | 77.92 | 8.03/20.93 | 40.35/78.55 | 60.5/46.4 |
| PR35766-B-24-3 | 3 | 6.59 | 2.1 | 3.14 | 25.02 | 99.17 | 13.36/18.59 | 73.43/85.88 | 58.03/48.65 |
| PR35769-B-37-3-1-2-1 | 3 | 6.49 | 2.05 | 3.16 | 25.67 | 81.67 | 5.03/15.99 | 23.7/69.55 | 64.89/58.26 |
| PR35789-B-37-3 | 3 | 6.49 | 2.04 | 3.19 | 23.17 | 92.75 | 11.44/6.91 | 49.03/31.83 | 62.42/57.49 |
| PR35887-1-21-2-1 | 3 | 6.67 | 2.08 | 3.2 | 22.22 | 85 | 18.46/26.1 | 83.45/94.8 | 64.81/52.36 |
| PR37139-3-1-3-1-2-1 | 3 | 6.69 | 2.04 | 3.28 | 23.95 | 92.92 | 12.3/18.55 | 61.87/84.4 | 60.36/45.07 |
| PR37160-11-5-1-1-1-1 | 3 | 6.83 | 2.11 | 3.23 | 22.15 | 70.75 | 6.57/12.17 | 40.25/54.57 | 66.61/43.78 |
| PR37252-2-1-1-1-2-2 | 3 | 6.66 | 2.04 | 3.26 | 23.47 | 96.83 | 6.76/8.76 | 36.03/43.28 | 58.37/49.57 |
| PR37934-3B-8-2-1 | 3 | 6.67 | 2.05 | 3.26 | 23.68 | 92.5 | 6.46/14.23 | 31.62/71.27 | 61.58/53.08 |
| PR37942-3B-5-3-2 | 3 | 6.64 | 2.11 | 3.15 | 22.78 | 88.17 | 13.93/16.29 | 76.05/86.27 | 61.96/49.43 |
| PR37990-3B-15-2 | 3 | 6.57 | 2.03 | 3.24 | 22.1 | 86.08 | 13.94/15.9 | 65.85/85.52 | 57.37/51.6 |
| PR38012-3B-3-1 | 3 | 6.78 | 1.99 | 3.4 | 21.67 | 87.33 | 9.94/15.81 | 56.2/69.76 | 59.66/38.74 |
| PR40078-B-12-2 | 3 | 6.89 | 2.07 | 3.34 | 22.72 | 87.92 | 20.45/25.78 | 90.1/100 | 56.64/52.5 |
| YTL126 | 3 | 6.64 | 1.92 | 3.45 | 22.32 | 80.33 | 5.45/12.47 | 31.37/54.82 | 62.05/40.21 |
| 6527 | 4 | 6.85 | 2.17 | 3.15 | 12.25 | 81.42 | 16.84/31.81 | 72.6/100 | 57.09/46.06 |
| A69-1 | 4 | 6.46 | 2.38 | 2.71 | 26.27 | 90.83 | 49.53/31.26 | 100/100 | 54.26/33.98 |
| ADRON 125 | 4 | 7.05 | 2.13 | 3.32 | 23.75 | 69.42 | 7/3.95 | 35.15/17.32 | 56.19/51.36 |
| AS996 | 4 | 6.79 | 2.12 | 3.2 | 20.63 | 85 | 30.09/29.63 | 100/73.57 | 60.47/39.09 |
| B11598C-TB-2-1-B-7 | 4 | 5.81 | 2.49 | 2.33 | 22.3 | 57.8 | 20.51/21.58 | 97.23/91.47 | 46.21/45.93 |
| BALILLA | 4 | 5.79 | 2.55 | 2.31 | 24.15 | 79.75 | 34.55/32.94 | 100/99.53 | 59.78/40.13 |
| BR 29 | 4 | 6.96 | 2.22 | 3.14 | 27.17 | 92.67 | 26.14/27.9 | 99.23/100 | 49.25/40.77 |
| BR 7232-6-2-3 | 4 | 6.59 | 2.24 | 2.94 | 26.45 | 75.5 | 26.34/39.71 | 100/100 | 53.41/50.66 |
| BR 7414-22-1 | 4 | 5.56 | 2.47 | 2.25 | 28.17 | 78.25 | 26.49/39.61 | 96.32/100 | 66.66/52.54 |
| BRRI DHAN 28 | 4 | 6.42 | 1.93 | 3.33 | 28.73 | 70.08 | 4.45/8.51 | 34.05/50.97 | 66.29/43.65 |
| CR 547-1-2-3 | 4 | 6.66 | 2.02 | 3.29 | 15.53 | 77.17 | 2.39/7.53 | 16.43/25.42 | 66/49.31 |
| CT 15671-15-4-5-1-1-M | 4 | 6.73 | 2.16 | 3.12 | 26.67 | 51.42 | 8.83/12.9 | 43.88/54.52 | 56.27/39.61 |
| CT 15673-8-1-4-1-6-M | 4 | 7.03 | 1.97 | 3.58 | 13.93 | 86.58 | 2.19/3.84 | 11.9/15.05 | 59.56/40.48 |
| CT 15675-7-1-7-1-2-M | 4 | 6.92 | 2.01 | 3.44 | 27.43 | 73.42 | 13.9/34.87 | 54.38/98.15 | 58.16/49.45 |
| CT 15679-17-1-1-2-3-M | 4 | 7 | 2.07 | 3.39 | 16.28 | 88.33 | 3.43/4.87 | 17.15/16.33 | 57.41/41.5 |
| CT 15691-4-3-3-1-1-M | 4 | 6.95 | 1.97 | 3.53 | 15.63 | 77.67 | 1.6/2.4 | 8.85/11.55 | 61.72/35.55 |
| CT 15716-6-1-2-2-2-M | 4 | 6.97 | 2.1 | 3.32 | 17.02 | 84.75 | 2.68/8.73 | 16.65/43.23 | 54.33/37.47 |
| CT 15765-13-3-6-2-1-M | 4 | 6.73 | 2.08 | 3.24 | 27.28 | 52.33 | 23.39/32.51 | 76.28/100 | 57.83/39.79 |
| CT 16658-5-2-2SR-2-3-6MP | 4 | 6.69 | 2.14 | 3.14 | 27.22 | 46 | 13.68/9.75 | 69.3/38.63 | 63.4/54.55 |
| CT 17323-1-1-2-2-2-2-M | 4 | 7.02 | 2.12 | 3.3 | 28.02 | 79.08 | 18.87/24.84 | 82.22/97.92 | 37.07/18.34 |
| CT 17334-13-3-1-2-1-M | 4 | 6.78 | 2.17 | 3.12 | 24.27 | 68.67 | 6.94/14.12 | 35.72/67.83 | 61.85/46.04 |
| CT 17379-32-5-1-1-5-M | 4 | 6.75 | 2.13 | 3.16 | 24.72 | 52.75 | 7.84/16.24 | 39.7/54.95 | 56.8/39.6 |
| CT 18148-6-9-3-3-2-MMP | 4 | 6.72 | 2.05 | 3.27 | 15.28 | 84.58 | 3.72/8.24 | 12.62/38.75 | 60.96/40.46 |
| CT 18148-6-9-5-1-2-MMP | 4 | 6.53 | 1.97 | 3.31 | 15.55 | 77.83 | 5.19/7.16 | 23.6/33.68 | 59.47/37.6 |
| CT 18154-5-1-4-2-2-M | 4 | 6.75 | 2.04 | 3.31 | 27.62 | 78.92 | 5.62/20.3 | 23.4/86.72 | 61.83/35.01 |
| CT 18657-2-1-2-1-2 | 4 | 6.87 | 2.02 | 3.4 | 26.38 | 81.58 | 6.25/3.72 | 43.17/17.03 | 55.59/54.71 |
| CT 18685-10-1-1-3-3 | 4 | 6.78 | 2.31 | 2.94 | 26.92 | 67.17 | 20.45/21 | 90.12/96.22 | 49.39/40.26 |
| CT 19561-3-20-2-3-2-2-M | 4 | 6.55 | 2.07 | 3.17 | 19.5 | 73.67 | 5.03/16.83 | 20.38/45.12 | 57.58/39.46 |
| FFZ 1 | 4 | 6.62 | 2.04 | 3.25 | 26.65 | 63.42 | 6.22/7.92 | 29.5/42.28 | 62.54/51.47 |
| GHAIYA | 4 | 5.4 | 2.66 | 2.03 | 26 | 37.58 | 46.72/51.8 | 100/100 | 54.8/48.22 |
| GUANG JIANG 1 | 4 | 5.73 | 2.46 | 2.35 | 25.42 | 70.25 | 28.75/31.51 | 99.32/100 | 60.35/47.99 |
| HARDINATH 1 | 4 | 6.36 | 2.23 | 2.85 | 26.78 | 65.42 | 15.1/25.06 | 93.38/100 | 61.5/45.9 |
| HHZ 12-DT-10-SAL-1-DT1^a^ | 4 | 6.66 | 2.06 | 3.24 | 19.82 | 65.92 | 1.51/10.6 | 10.5/40.7 | 58/53.6 |
| HHZ 12-DT-10-SAL-1-DT1^a^ | 4 | 6.55 | 2.03 | 3.24 | 19.72 | 57.83 | 1.53/1.63 | 9.38/9.68 | 62.78/58.43 |
| HHZ 5-DT-8-DT1-Y1 | 4 | 6.76 | 1.97 | 3.42 | 25.38 | 67.25 | 6.75/10.44 | 30.58/48.37 | 58.07/44.24 |
| HHZ 5-SAL 9-Y 3-Y 1 | 4 | 6.78 | 2.16 | 3.15 | 24.9 | 73.42 | 23.58/25.79 | 84.92/97.27 | 50.68/42.41 |
| HHZ 8-SAL 6-SAL 3-Y2 | 4 | 6.55 | 1.93 | 3.39 | 14.88 | 85 | 1.92/3.73 | 10.23/19.62 | 59.29/43.62 |
| HHZ 8-SAL9-DT2-Y1 | 4 | 6.66 | 1.99 | 3.36 | 15.22 | 72.25 | 0.46/1.75 | 5.25/11.72 | 64.12/53.36 |
| HUA 565 | 4 | 6.48 | 1.76 | 3.69 | 14.62 | 86.5 | 0.44/1.02 | 5.87/7.96 | 59.35/46.89 |
| IR 03A568 | 4 | 6.61 | 1.91 | 3.46 | 27.73 | 77.42 | 5.32/4.26 | 26.02/19.37 | 53.12/51.01 |
| IR 05N168 | 4 | 6.84 | 2.11 | 3.25 | 23.28 | 83.92 | 24.88/30.73 | 95.22/100 | 60.49/40.32 |
| IR 05N173 | 4 | 6.87 | 2.17 | 3.17 | 27.18 | 82.92 | 8.77/23.8 | 41.1/98.93 | 39.88/33.11 |
| IR 05N496 | 4 | 6.87 | 2.11 | 3.25 | 22.05 | 79.75 | 32.76/39.41 | 97.18/100 | 39.67/24.39 |
| IR 06A150 | 4 | 6.45 | 2.06 | 3.13 | 22.98 | 77.5 | 15.26/22.63 | 55.83/94.08 | 37.54/44.92 |
| IR 06A152 | 4 | 6.55 | 1.9 | 3.46 | 21.9 | 65 | 2.85/13.64 | 14.62/69.35 | 47.06/28.22 |
| IR 06N154 | 4 | 6.73 | 2.12 | 3.18 | 17.87 | 79.92 | 4.99/4.72 | 19.68/20.23 | 63.94/51.32 |
| IR 06N211 | 4 | 6.89 | 1.9 | 3.63 | 18.45 | 84.58 | 1.16/1.66 | 7.58/11.08 | 60.29/40.68 |
| IR 06N234 | 4 | 6.8 | 2.02 | 3.38 | 23.42 | 74.5 | 8.96/16.04 | 41.07/61.22 | 48.6/43.85 |
| IR 07A179 | 4 | 6.99 | 1.87 | 3.74 | 20.78 | 88.5 | 3.63/10.49 | 24.62/52.48 | 51.82/36.41 |
| IR 08M110 | 4 | 6.83 | 2.17 | 3.16 | 25.63 | 79.17 | 31.84/38 | 97.95/100 | 54.83/45.63 |
| IR 09A138 | 4 | 6.65 | 2 | 3.33 | 22.77 | 87.58 | 17.39/14.83 | 61.76/60.67 | 61.08/44.45 |
| IR 09A228 | 4 | 6.68 | 2.01 | 3.32 | 22.9 | 91.5 | 19.38/17.83 | 64.72/77.9 | 60.11/48.52 |
| IR 09N126 | 4 | 6.76 | 2.13 | 3.18 | 23.4 | 94.83 | 29.49/34.26 | 81.78/100 | 45.9/31.41 |
| IR 10A107 | 4 | 6.64 | 2.14 | 3.11 | 23.25 | 84.17 | 15.96/16.58 | 69/82.92 | 59.81/57.22 |
| IR 10A131 | 4 | 6.64 | 2.06 | 3.23 | 24.9 | 69.92 | 1.29/1.88 | 9.68/9.87 | 62.65/55.33 |
| IR 10F403 | 4 | 6.54 | 1.99 | 3.29 | 22.75 | 82 | 1.87/26.33 | 9.6/77 | 64.96/39.83 |
| IR 10N291 | 4 | 6.49 | 2.27 | 2.87 | 24.53 | 89.25 | 32.25/45.65 | 80.3/100 | 55.05/33.9 |
| IR 10N303 | 4 | 6.89 | 2.06 | 3.34 | 26.64 | 56 | 14.2/23.51 | 77.58/94.42 | 46.44/28.33 |
| IR 10N304 | 4 | 6.92 | 2.07 | 3.34 | 27.67 | 78.33 | 3.52/16.82 | 17.25/70.13 | 62.26/45.85 |
| IR 24 | 4 | 6.73 | 2.17 | 3.1 | 15.62 | 81.42 | 5.8/9.68 | 25.47/44.27 | 58.59/48.85 |
| IR 55423-01 | 4 | 5.97 | 2.28 | 2.63 | 20.97 | 66.58 | 32.57/38.6 | 100/100 | 66.07/53.8 |
| IR 77384-12-35-3-12-1-B | 4 | 6.9 | 1.97 | 3.5 | 22.7 | 51.42 | 8.26/13.6 | 37.43/55.83 | 50.17/26.77 |
| IR 77674-3B-8-2-2-8-2-AJY10 | 4 | 6.91 | 1.96 | 3.53 | 24.2 | 70.42 | 17.91/14.1 | 72.72/70.62 | 43.23/19.64 |
| IR 78875-190-B-1-3 | 4 | 6.43 | 2.04 | 3.16 | 21.12 | 67.67 | 10.78/40.37 | 48.83/100 | 59.49/46.77 |
| IR 80340-23-B-12-6-B | 4 | 6.67 | 2.16 | 3.09 | 19.73 | 84.92 | 10.15/7.12 | 55.28/36.63 | 59.06/44.1 |
| IR 87520-44-3-1-2 | 4 | 6.78 | 2.12 | 3.21 | 22.57 | 84.58 | 31.39/22.58 | 99.55/92.3 | 35.8/44.39 |
| IRBB57 | 4 | 6.66 | 2.1 | 3.18 | 14.75 | 86.08 | 9.62/19.59 | 48.28/88.65 | 51.73/42.72 |
| IRBB66 | 4 | 6.63 | 1.98 | 3.36 | 12.72 | 82.25 | 6.13/13.66 | 28.07/57.33 | 43.15/39.55 |
| IRGA 318-11-6-9-2B | 4 | 6.87 | 1.86 | 3.7 | 27.22 | 49 | 2.41/2.09 | 11.48/11.8 | 58.07/46.9 |
| IRGA 318-11-9-2A | 4 | 6.99 | 1.9 | 3.69 | 27.97 | 40.5 | 1.5/0.54 | 7.25/5.18 | 62.9/48.29 |
| IRGA 659-1-2-2-2 | 4 | 6.58 | 2.21 | 2.98 | 16.02 | 83.83 | 1.19/0.69 | 8.97/7.75 | 58.61/60.89 |
| IRRI 129 | 4 | 5.56 | 2.28 | 2.45 | 27.28 | 72.5 | 21.5/25.46 | 82.17/99.87 | 64.01/61.18 |
| KHUDWANI ACC 193 | 4 | 6.73 | 1.98 | 3.41 | 21.2 | 80 | 4.56/4.58 | 27.42/17.9 | 62.09/53.2 |
| MILYANG 46 | 4 | 5.72 | 2.35 | 2.44 | 16.47 | 83.92 | 13.74/9.34 | 73.57/45.92 | 65.79/56.88 |
| MINGHUI 63 | 4 | 6.52 | 2.21 | 2.94 | 18.63 | 73.83 | 13.35/21.99 | 71.47/81.18 | 57.82/48.45 |
| MTU1115 | 4 | 5.63 | 1.99 | 2.83 | 21.73 | 75.58 | 23.57/21.99 | 73.07/74.03 | 56.46/47.07 |
| NERICA-L-17 | 4 | 6.9 | 2.04 | 3.38 | 28.57 | 82 | 10.49/25.38 | 57.68/82.9 | 39.19/36.36 |
| NERICA-L-19 | 4 | 6.98 | 2.02 | 3.46 | 28.17 | 81.67 | 13.39/23.72 | 71.28/95.87 | 54.63/30.22 |
| NSIC Rc 158 | 4 | 6.92 | 2.1 | 3.3 | 22.9 | 78.33 | 16.75/32.78 | 78.22/95.37 | 56.53/46.69 |
| NSIC Rc 222 | 4 | 6.81 | 2.12 | 3.21 | 23.68 | 86.83 | 15.99/25.29 | 76.85/74.87 | 54.05/34.80 |
| OM 4900 | 4 | 6.42 | 2.26 | 2.89 | 14.48 | 83.92 | 12.05/35.19 | 66.92/100 | 60.02/57.18 |
| OM 6600 | 4 | 6.7 | 2.12 | 3.17 | 14.68 | 80.33 | 24.23/24.97 | 99.19/87.2 | 59.16/45.48 |
| PAU-201 | 4 | 6.53 | 2.12 | 3.09 | 14.58 | 84.75 | 3.21/10.98 | 17.9/42.58 | 63.25/39.66 |
| PEDEAPROZ 50 | 4 | 6.7 | 2.19 | 3.07 | 27.17 | 78.58 | 11.34/8.8 | 52.27/42.23 | 58.86/53.16 |
| PK 7392-10-1-1-1-1 | 4 | 6.57 | 2.02 | 3.33 | 25.15 | 50.92 | 2.2/8.03 | 14.37/43.93 | 46.35/52.52 |
| PR-114 | 4 | 6.8 | 1.96 | 3.47 | 26.43 | 51.67 | 5.95/9.63 | 18.73/46.63 | 52.67/41.11 |
| PR-116 | 4 | 6.68 | 2.16 | 3.1 | 26.23 | 92.92 | 7.01/13.98 | 29.8/68.72 | 53.35/45.81 |
| PR33282-B-8-1-1-1-1-1 | 4 | 6.15 | 2.19 | 2.81 | 2 | 96.42 | NA | NA | 66.15/40.38 |
| PR33319-9-1-1-5-3-5-4-1 | 4 | 6.74 | 2.21 | 3.05 | 23.12 | 56.42 | 20.03/30.89 | 67.44/90.98 | 56.12/47.65 |
| PR35805-B-9-2-3-2-3 | 4 | 6.49 | 2.15 | 3.02 | 6.2 | 90.33 | NA | NA | 67.41/45.36 |
| PR36723-B-1-3-3-3-2 | 4 | 6.78 | 2.2 | 3.09 | 22.5 | 80 | 10.34/15.38 | 55.37/77.83 | 62.01/48.6 |
| PR37126-PB-2-3-4-10-8 | 4 | 6.62 | 2.25 | 2.94 | 16.43 | 84 | 3.37/1.15 | 17.73/7.18 | 64.75/62.87 |
| PR37171-1-1-1-2-1-1-1 | 4 | 6.11 | 2.08 | 2.94 | 15.42 | 86.58 | 4.53/18.4 | 25.95/54.85 | 64.48/58.03 |
| PR37246-2-3-2-1-1-2-1 | 4 | 6.7 | 2.02 | 3.32 | 23.75 | 66.25 | 3/17.34 | 15.85/41.67 | 63.5/48.42 |
| PR37946-B-28-3-2 | 4 | 6.75 | 2.26 | 2.99 | 26.9 | 79.5 | 34.8/39.49 | 99.97/100 | 53.86/31.06 |
| PR37951-3B-37-1-2 | 4 | 6.84 | 2.06 | 3.33 | 23.18 | 64.92 | 5.23/10.37 | 20.43/51.15 | 61.1/57.81 |
| PSBRC84 | 4 | 6.46 | 2.08 | 3.1 | 24.75 | 95.33 | 9.52/9.01 | 48.28/45.12 | 60.65/51.9 |
| PSBRC94 | 4 | 6.56 | 2.08 | 3.15 | 25.95 | 88.08 | 6.14/21.45 | 38.7/79.25 | 51.4/44.55 |
| PUNGSANBYEO | 4 | 6.14 | 2.37 | 2.6 | 15.88 | 79.08 | 20.29/24.71 | 95.4/96.17 | 66.9/43.35 |
| SACG-04 | 4 | 5.68 | 2.52 | 2.28 | 25.8 | 38.25 | 43.79/39.82 | 100/100 | 60.63/52.03 |
| SAGC-02 | 4 | 6.73 | 2.54 | 2.66 | 27.25 | 52.08 | 38.44/40.43 | 100/100 | 56.7/43.27 |
| SAGC-08 | 4 | 5.45 | 2.52 | 2.17 | 12.95 | 71.92 | 21.99/23.69 | 99.68/100 | 62.91/38.03 |
| SONALEE | 4 | 7.05 | 1.83 | 3.87 | 16.73 | 80.25 | 2.23/10.09 | 12.08/48.58 | 47.43/39.21 |
| TE QING | 4 | 5.83 | 2.44 | 2.45 | 24.1 | 44.92 | 29.48/37.84 | 72.37/100 | 64.92/61.7 |
| TME80518 | 4 | 6.46 | 1.9 | 3.4 | 16.85 | 78.17 | 2.12/2.82 | 12.85/10.83 | 61.68/51.95 |
| WAB96-1-1 | 4 | 6.84 | 1.97 | 3.47 | 23.67 | 78.58 | 13.89/10.91 | 75.53/64.1 | 57.53/44.76 |
| WANXIAN 763 | 4 | 6.62 | 2.34 | 2.84 | 14.28 | 73.67 | 17.14/28.82 | 80.05/99.7 | 58.2/39.12 |
| WEED TOLERANT RICE 1 | 4 | 5.56 | 2.41 | 2.31 | 16.6 | 79.75 | 22.59/18.77 | 95.6/85.72 | 62.82/47.32 |
| Yangdao 6 | 4 | 6.73 | 2.26 | 2.98 | 16.25 | 78.17 | 9.2/15.86 | 41.45/71.3 | 49.51/48.38 |
| ZGY1 | 4 | 6.4 | 2.12 | 3.02 | 26.67 | 60.67 | 23.94/25.21 | 85.93/84.75 | 60.81/41.83 |
| ZH1 | 4 | 6.36 | 2.07 | 3.08 | 26.3 | 64.08 | 11.86/8.52 | 54.73/37.8 | 59.63/49.72 |
| ZHONGZU 14 | 4 | 6.46 | 1.94 | 3.32 | 16.47 | 72.25 | 0.96/2.08 | 6.3/8.8 | 66.95/54.02 |
| ZX115 | 4 | 6.73 | 2.12 | 3.18 | 19.95 | 79.17 | 9.82/14.48 | 46.1/76.38 | 49.48/32.2 |

For AC, GC, GL, GW and LWR, only average value of 6 environments was presented.

The data before and after “/” represent average value of DS and WS respectively.

^a^: Lines with duplicates, but from different source and with small difference in genotype

Supplementary table 2 Fifty three SSR markers for population structure and kinship analysis

| Marker | Forward Primer (5'-3') | Reverse Primer (5'-3') | Chr | Position(Mb) |
| --- | --- | --- | --- | --- |
| RM495 | AATCCAAGGTGCAGAGATGG | CAACGATGACGAACACAACC | 1 | 0.21 |
| RM581 | ACATGCGTGATCAACAATCG | AATTGGATGTGGATGCACG | 1 | 9.1 |
| RM306 | GTACGTAAACGCGGAAGGTGACG | CGACGTACGAGATGCCGATCC | 1 | 12.26 |
| RM113 | CACCATTGCCCATCAGCACAAC | TCGCCCTCTGCTGCTTGATGGC | 1 | 19.16 |
| RM443 | GCGAAGCCCAATCTGAAGAAGC | CCAGTCCCAGAATGTCGTTTCG | 1 | 28.67 |
| RM529 | TTCACCACAACGATAGAGACTTCTGG | GGGAAGAAGATGACAGAGCAAGC | 1 | 41 |
| RM109 | GCCGCCGGAGAGGGAGAGAGAG | CCCCGACGGGATCTCCATCGTC | 2 | 0.18 |
| RM322 | CAAGCGAAAATCCCAGCAG | GATGAAACTGGCATTGCCTG | 2 | 7.43 |
| RM106 | CGTCTTCATCATCGTCGCCCCG | GGCCCATCCCGTCGTGGATCTC | 2 | 25.14 |
| RM530 | TTCTTTATTCCCTCGCACTGACC | CAATGATGCCACAAACCGTAACC | 2 | 30.53 |
| RM132 | ATCTTGTTGTTTCGGCGGCGGC | CATGGCGAGAATGCCCACGTCC | 3 | 0.99 |
| RM554 | GTTCGTCCGTCTCTCGTCTC | CCCAAAAATCTGTGCCTCTC | 3 | 12.14 |
| RM156 | GCCGCACCCTCACTCCCTCCTC | TCTTGCCGGAGCGCTTGAGGTG | 3 | 17.67 |
| RM416 | GGGAGTTAGGGTTTTGGAGC | TCCAGTTTCACACTGCTTCG | 3 | 31.19 |
| RM85 | CCAAAGATGAAACCTGGATTG | GCACAAGGTGAGCAGTCC | 3 | 36.29 |
| RM551 | CTTACTCCATTGGGCTGGAACC | TGTAGGGTGGTAAGAGATCCACTCC | 4 | 0.17 |
| RM261 | CTACTTCTCCCCTTGTGTCG | TGTACCATCGCCAAATCTCC | 4 | 6.56 |
| RM437 | ATCCCTCCTCTGCTCAATGTTGG | TCAGGGAGGGTCCTAGCTACTGG | 4 | 8.32 |
| RM185 | GGCTCTCCATCTCCATTGATCC | GAGTTGTTGGGAGGGAGAAAGG | 4 | 18.56 |
| RM119 | CATCCCCCTGCTGCTGCTGCTG | CGCCGGATGTGTGGGACTAGCG | 4 | 21.23 |
| RM127 | CGAAGCTTTCGGTGGGATAGC | ACCTTGAGCGAGTCCTTGAACG | 4 | 34.5 |
| RM153 | CCTCGAGCATCATCATCAGTAGG | TCCTCTTCTTGCTTGCTTCTTCC | 5 | 0.17 |
| RM330A | CAATGAAGTGGATCTCGGAG | CATCAATCAGCGAAGGTCC | 5 | 6.57 |
| RM173 | CCTACCTCGCGATCCCCCCCTC | CCATGAGGAGGAGGCGGCGATC | 5 | 21.62 |
| RM188 | TCCGCCTCTCCTCTCGCTTCCC | GCAACGCACAACCGAACCGAGC | 5 | 22.65 |
| RM136 | GAGAGCTCAGCTGCTGCCTCTAGC | GAGGAGCGCCACGGTGTACGCC | 6 | 8.75 |
| RM3 | ACACTGTAGCGGCCACTG | CCTCCACTGCTCCACATCTT | 6 | 19.5 |
| RM541 | TATAACCGACCTCAGTGCCC | CCTTACTCCCATGCCATGAG | 6 | 19.51 |
| RM275 | GCATTGATGTGCCAATCG | CATTGCAACATCTTCAACATCC | 6 | 24.32 |
| RM103 | ATCAGCAGCATTCAGCATTTGG | CCGGACGATGTGTATATCTCTTGG | 6 | 30.89 |
| RM125 | ATCAGCAGCCATGGCAGCGACC | AGGGGATCATGTGCCGAAGGCC | 7 | 5.48 |
| RM11 | TCTCCTCTTCCCCCGATC | ATAGCGGGCGAGGCTTAG | 7 | 19.26 |
| RM336 | GTATCTTACAGAGAAACGGCATCG | GGTTTGTTTCAGGTTCGTCTATCC | 7 | 21.87 |
| RM234 | TTCAGCCAAGAACAGAACAGTGG | CTTCTCTTCATCCTCCTCCTTGG | 7 | 25.47 |
| RM172 | TGCAGCTGCGCCACAGCCATAG | CAACCACGACACCGCCGTGTTG | 7 | 29.56 |
| RM152 | AAGGAGAAGTTCTTCGCCCAGTGC | GCCCATTAGTGACTGCTCCTAGTCG | 8 | 0.68 |
| RM544 | GCTGCACCCTCTCTCAATAAATGC | GTGGACAGCTCGAAACGAAGC | 8 | 5.1 |
| RM223 | GAGTGAGCTTGGGCTGAAAC | GAAGGCAAGTCTTGGCACTG | 8 | 20.52 |
| RM256 | GACAGGGAGTGATTGAAGGC | GTTGATTTCGCCAAGGGC | 8 | 24.14 |
| RM409 | CCGTCTCTTGCTAGGGATTC | GGGGTGTTTTGCTTTCTCTG | 9 | 14.37 |
| RM434 | TCTCTAGTTGCCTCATCCCTCTAACC | GGCTCAACCTCTATATTTGCTGATCG | 9 | 15.66 |
| RM245 | ATGCCGCCAGTGAATAGC | CTGAGAATCCAATTATCTGGGG | 9 | 22.27 |
| RM222 | CTTAAATGGGCCACATGCG | CAAAGCTTCCGGCCAAAAG | 10 | 2.6 |
| RM311 | TGGTAGTATAGGTACTAAACAT | TCCTATACACATACAAACATAC | 10 | 9.49 |
| RM184 | ATCCCATTCGCCAAAACCGGCC | TGACACTTGGAGAGCGGTGTGG | 10 | 16.1 |
| RM286 | GGCTTCATCTTTGGCGAC | CCGGATTCACGAGATAAACTC | 11 | 0.38 |
| RM441 | AAGGGAGTAGCCTCTCCATCTCC | GTGCTGACTCCTCTCCCTGTCC | 11 | 6.08 |
| RM287 | GGCTACACCTACACGCGAGAACC | AGATGCATGGAATGCCTGTTTGG | 11 | 16.73 |
| RM457 | GCACAAGTTGATACTCTCCTCTGACG | CCACCATTATCTGCTCCATCACC | 11 | 19.03 |
| RM144 | CATGTTGTGCTTGTCCTACTGC | AGCTAGAGGAGATCAGATGGTAGTGC | 11 | 28.25 |
| RM247 | AAGGCGAACTGTCCTAGTGAAGC | CAGGATGTTCTTGCCAAGTTGC | 12 | 3.19 |
| RM277 | CGGTCAAATCATCACCTGAC | CAAGGCTTGCAAGGGAAG | 12 | 18.29 |
| RM17 | TGCCCTGTTATTTTCTTCTCTC | GGTGATCCTTTCCCATTTCA | 12 | 26.95 |

Supplementary table 3 Markers on starch systhesis genes

| Primers | Sequence | Type | Chr | Position (Mb) | Locus | References |
| --- | --- | --- | --- | --- | --- | --- |
| AGPL1-F | CGTTCAGGTTCAGGCAATCA | STS | 5 | 28.79 | Promoter region 5bp/8bp Del | Tian et al. 2010 |
|  | GGAAGGGTGGTGATGTGGAG |  |  |  |  |  |
| AGPL1-IF | GCAGGGTATGAGGCG | ARMS | 5 | 28.79 | Intron 11 SNP (A/G) | Tian et al. 2010 |
|  | GATATAGAAAGTTCATTCCAGGT |  |  |  |  |  |
|  | GTATTTGGTCTGCTTCAAAA |  |  |  |  |  |
|  | AAGCCTTCAGGTCAGTATCT |  |  |  |  |  |
| AGPL2-1-IF | GTTAACCAGCCAGAAGACTG | ARMS | 1 | 25.69 | Intron 1 SNP(T/G) | Tian et al. 2010 |
|  | AACTACACCACATCAATTACCA |  |  |  |  |  |
|  | TGTCGTTTTCTTTTTATTGAA |  |  |  |  |  |
|  | TATATACGCACTTCCTGTGC |  |  |  |  |  |
| AGPL2-2-IF | CGTGTACCTAACGATGCGT | ARMS | 1 | 25.69 | 3'-UTR SNP(G/T) | Tian et al. 2010 |
|  | AAGTTTATTTGTCAGTATTGAACTC |  |  |  |  |  |
|  | TGTAAGGAAATGAAGAGCG |  |  |  |  |  |
|  | CGTAATACTAGGAGGGGTCA |  |  |  |  |  |
| AGPL2-F | CAATCGCTGCCATCGGTTG | STS | 1 | 25.69 | 3'-UTR 5bp Del | Tian et al. 2010 |
|  | TTCCACATCGTTAGGTACACG |  |  |  |  |  |
| AGPS1-1-F | TCTATTCTCAGCCCTCCAACC | STS | 9 | 7.25 | Promotor 29bp/39bp Del | Tian et al. 2010 |
|  | GTGTGTTTAGAGGTGCTTTTCG |  |  |  |  |  |
| AGPS1-2-F | TACGCTATGCTCTTGAAAC | STS | 9 | 7.25 | Intron 4, 31bp Del | Tian et al. 2010 |
|  | TATCTTCCCAGTAACCATCA |  |  |  |  |  |
| AGPS2-F | GACGAAAAGTGAAAGTTGC | STS | 8 | 15.54 | Intron 6 AT Del | Li et al. 2008 |
|  | GGTTCTTGATGCTGATGTG |  |  |  |  |  |
| AGPS2-IF | ATGTAAACTGTTAGAATCGAATAC | ARMS | 8 | 15.54 | Intron 2 SNP(C/T) | Li et al. 2008 |
|  | CATCCTTATCATTAAGTCTGCTA |  |  |  |  |  |
|  | CAAAAGCACTCATTGGAAC |  |  |  |  |  |
|  | GTCAAGAGCCTATGGGAAC |  |  |  |  |  |
| BEI-1-F | TGCTACATAACACGCATACAAAGT  AGACAAAAGCGAAAGGTAATGAG | STS | 6 | 30.91 | Promoter 335bp Del | Tian et al.2010; Liu et al. 2004; He et al. 2006 |
| BEI-1-IF | TGTTAGCTTCATGTTCTTATTTC | ARMS | 6 | 30.91 | Intron 6 SNP(T/C) | Tian et al. 2010 |
|  | CAATTCAGTAACTGTCGCA |  |  |  |  |  |
|  | CCTGTAGGTTATCAAATTGC |  |  |  |  |  |
|  | CAGTGTACATTTCCTTGTCC |  |  |  |  |  |
| BEI-2-F | GTGGGGAAAACAAGTAAGTCTG | STS | 6 | 30.91 | Intron 2, 15bp Insert | Tian et al. 2010 |
|  | AGTTCCATCAGAAGAATCAGGG |  |  |  |  |  |
| BEI-2-IF | GGCTATCAGGCTTACTATCGT | TSP | 6 | 30.91 | Exon 14 SNP(T/C) | Han et al. 2004; Liu et al. 2004; He et al. 2006 |
|  | TTTCAACAACCGCCCTAACTCA |  |  |  |  |  |
|  | CCTTTCTTGCCACAATCGTCC |  |  |  |  |  |
| BEI-3-F | GGAAATGGGAGTCGCC | STS | 6 | 30.91 | Intron 1, 12bp Insert | Tian et al. 2010 |
|  | CGAAGAAACCACGCTCA |  |  |  |  |  |
| BEI-4-F | ATTTCTTTGGCCACAGGCGA  CCCAGATTCGGAACAAGAAC | SSR | 6 | 30.91 | Intron 2 (CT)n | Bao et al. 2002b; Bao et al. 2006 |
| BEIIa-1-F | CACCAATTATATTAGCGTGCTCC | STS | 4 | 20.05 | 3'-UTR 22bp Del | Tian et al. 2010 |
|  | CGTGGCTCTTGGCTCTCTTG |  |  |  |  |  |
| BEIIa-2-F | CCATCACCTCAAATACATCACTC | STS | 4 | 20.05 | 3'-UTR 13bp Del | Tian et al. 2010 |
|  | AGACTGGAATGCCCCTTAGG |  |  |  |  |  |
| BeIIb-1-F | TCGGTCCTAATATTTTGCGCTG | STS | 2 | 19.36 | 5'-UTR 13bp Indels | Yan et al. 2005; Yan et al. 2007 |
|  | CCTTAACTTGACACCGAATCCG |  |  |  |  |  |
| BeIIb-2-F | AAGGTTAGCATTGGTTGGTGAG | STS | 2 | 19.36 | 3'-UTR 28bp Indels | Tian et al. 2010 |
|  | TCTCCTTGAACAGCGACAGC |  |  |  |  |  |
| BeIIb-3-F | GTGGGGTTCTCAACTTAGC | STS | 2 | 19.36 | Intron 6, 56bp/32bp Insert | Tian et al. 2010 |
|  | CATCAGCATTGTTAGGCAG |  |  |  |  |  |
| BEIIb-IF | GAAGGAGCAAGAAAAACAAC | ARMS | 2 | 19.36 | 3'-UTR SNP(C/G) | Han et al. 2004; Liu et al. 2004; He et al. 2006; Bao et al. 2006; Lee et al. 2009 |
|  | TTCACAGATTGCTGGCTAC |  |  |  |  |  |
|  | ACCTGCGTTGTCTATGCTC |  |  |  |  |  |
|  | CACCTTGAAATCACTGGAA |  |  |  |  |  |
| GBSSI-1-IF | CCGAAGAACATCTGCAAG***G*** | TSP | 6 | 1.77 | Intron 1 SNP(G/T) | Wang et al. 2005; Hirano et al. 1998; Isshiki et al. 1998 |
|  | TTTGGCTCTGAGGCACTGACG |  |  |  |  |  |
|  | TCCAGCCCAACACCTTACAGAA |  |  |  |  |  |
| GBSSI-2-IF | CGACCGTGTGTTCATAGG | ARMS | 6 | 1.77 | Exon 4 SNP(A/G) | Mikami et al. 2008 |
|  | CCAGGAATGACGGATTGT |  |  |  |  |  |
|  | GTTTTTGTGGTGCAATTCAT |  |  |  |  |  |
|  | GATCTTCTCACCGGTCTTTC |  |  |  |  |  |
| GBSSI-3-IF | CCCATACTTCAAAGGAACCTC | ARMS | 6 | 1.77 | Exon 6 SNP(A/C) | Larkin et al. 2003 |
|  | CTTGAGATCAATTGTAACTCACTAT |  |  |  |  |  |
|  | TTCAGGTTTGGGGAAAGA |  |  |  |  |  |
|  | CCATTGGGCTGGTAGTTG |  |  |  |  |  |
| GBSSI-4-IF | CTGGAGGAACAGAAGGTCC | ARMS | 6 | 1.77 | Exon 10(C/T) | Larkin et al. 2003 |
|  | GGCCATGACGTCCGA |  |  |  |  |  |
|  | TTCTGACAAGGCAAGAATGA |  |  |  |  |  |
|  | AAAGCCTCACCCCTTCTAAT |  |  |  |  |  |
| GBSSI-F | CACAGCAACAGCTAGACAACCAC | STS | 6 | 1.77 | Exon 2, 23bp Indels | Wanchana et al. 2003; Tian et al. 2010 |
|  | CACGACGACGGAGGGGAAC |  |  |  |  |  |
| GBSSII-F | TTGCTGCGAATTATCTGCG | STS | 7 | 12.92 | Intron 12, 33bp Del | Tian et al. 2010 |
|  | ACCTCCTCCCACTTCTTTGC |  |  |  |  |  |
| GBSSII-IF | TGTGGTATACTTCTTATTAGACTTC | ARMS | 7 | 12.92 | 5’UTR SNP(C/T) | Tian et al. 2010 |
|  | CTGATACCTTGAGATGGTGTATA |  |  |  |  |  |
|  | CGCCAGACAAACTGTAATC |  |  |  |  |  |
|  | GGTGTCTTTCAGTGCTCCT |  |  |  |  |  |
| ISA1-F | ATAGATGCTAATGTGATGTGGC | STS | 8 | 25.77 | Promoter 10bp Del | He et al. 2006; Tian et al. 2010 |
|  | TGGTATAGGCACAACCGTAGA |  |  |  |  |  |
| ISA1-IF | AGAGTTTTTGAGAGTGGCAA | ARMS | 8 | 25.77 | Promoter SNP(G/A) | Tian et al. 2010 |
|  | TTTGAAAAAACAGGACGC |  |  |  |  |  |
|  | TTTTAATCTCCCTGTCTTGC |  |  |  |  |  |
|  | ATTTTGTGAGCAATGTTGAA |  |  |  |  |  |
| ISA1-2-F | TGTGGGAATACCTTCAACTG | STS | 8 | 25.77 | Intron 8 10bp Insert | Tian et al. 2010 |
|  | ATAAAACCCTTACAGGCTTG |  |  |  |  |  |
| ISA2-1-IF | AAGAACCGGGTCAAACGT | ARMS | 5 | 19.07 | Iso2_GA_Ref960 | Kharabian-Masouleh et al. 2011 |
|  | CCCTTCTTGAGGCGAGAG |  |  |  |  |  |
|  | CAGGCCCTCTGGTTGAGA |  |  |  |  |  |
|  | ATGCCCCTTTCCTTGTGA |  |  |  |  |  |
| ISA2-2-IF | CGTACGGGTCCAACAGTGAGA | ARMS | 5 | 19.07 | Iso2_GA_Ref1712 | Kharabian-Masouleh et al. 2011 |
|  | CGGTGGCATCCGTCG |  |  |  |  |  |
|  | CCTTCCCCAGTTGTAGCTCG |  |  |  |  |  |
|  | AATGCCAATGCCGTTTCTCT |  |  |  |  |  |
| ISA2-3-IR | ACCTCCTTCGACCCGA | TSP | 5 | 19.07 | Iso2_GA_Ref2067 | Kharabian-Masouleh et al. 2011 |
|  | CCGGTGGATGTACGGATCG |  |  |  |  |  |
|  | CGGCGGAAGAGTTGTAGCG |  |  |  |  |  |
| PUL-1-F | AGAGAAGGAGAAAGAAGTGGAGAC | STS | 4 | 4.39 | promoter region 13bp Del | Tian et al. 2010;Yan et al. 2010 |
|  | GTCCAAACTGAATCACTCAATCG |  |  |  |  |  |
| PUL-2-F | GACAACCGTCCGCTTTAGTTTC | STS | 4 | 4.39 | promoter region 6bp/9bp Del | Tian et al. 2010 |
|  | GCATTTGAGAGGGTTTGGATTC |  |  |  |  |  |
| PUL-3-F | CTGTATGGACTGAGTAGTCGATGG | STS | 4 | 4.39 | Intron 9 53bp Del | Tian et al. 2010 |
|  | TGAGCCTCATCTGCCAGAGT |  |  |  |  |  |
| PUL-4-F | TACACCATCCTCACTACCA | STS | 4 | 4.39 | Intron 15 18bp Insert | Tian et al. 2010 |
|  | GCAACATCTAAAACACCAA |  |  |  |  |  |
| PUL-5-F | ATTGGCATTTGTAAGTTTC | STS | 4 | 4.39 | Intron 24 240bp Del | Tian et al. 2010 |
|  | CAATCTTGGTTTTATCCTG |  |  |  |  |  |
| PUL-6-F | ATTTAACTGTATGGACTGAG | STS | 4 | 4.39 | Intron 24 240bp Del | Tian et al. 2010 |
|  | GATACCAACCAAACAAGA |  |  |  |  |  |
| SSI-1-F | GATCCGTTTTTGCTGTGCCC | SSR | 6 | 3.08 | 5'-UTR SSR | Bao et al. 2002; Bao et al. 2006 |
|  | CCTCCTCTCCGCCGATCCTG |  |  |  |  |  |
| SSI-1-IF | TCCTAATTAGTGCATACGCTTT | ARMS | 6 | 3.08 | Promoter SNP (C/T) | Tian et al. 2010 |
|  | CGCACCACTTAAATTCTCG |  |  |  |  |  |
|  | GGTAGGGTAGGTCAATCTGG |  |  |  |  |  |
|  | CGTGAAAAAGAAGTCTCGAA |  |  |  |  |  |
| SSI-2-F | CTTCTATCCATTCCTTAATCCCA | STS | 6 | 3.08 | Intron 14 7bp Del | Tian et al. 2010 |
|  | ATGCTATTGATGTTAAGAGGGC |  |  |  |  |  |
| SSI-2-IF | GAGATACATGAACGGGGACT | ARMS | 6 | 3.08 | Exon2 SNP(C/T) | Tian et al. 2010 |
|  | CGTTTGCAAAATTTTTGTTAAG |  |  |  |  |  |
|  | TGCTCTTCGTGGTCATCG |  |  |  |  |  |
|  | CAGAATCCCTATACTCGTGAAA |  |  |  |  |  |
| SSIIa-F | CCAATACCGTAAACTAGCGACTATG | STS | 6 | 6.75 | 5'-UTR 9bp Del | Tian et al. 2010 |
|  | TACAGGTAGAATGGCAGTGGTG |  |  |  |  |  |
| SSIIa-IF | CCAGAGCTGGAGGGG***TT*** | TSP | 6 | 6.75 | Exon 8 GC/TT | Tian et al. 2010; Cuevas et al. 2010 |
|  | TCATACGGGAGAACGACTGGAA |  |  |  |  |  |
|  | AACGCACAGCTCGGTCAGC |  |  |  |  |  |
| SSIIaSNP2FF | AGAACGACTGGAAGATGAACG | ASP | 6 | 6.75 | Exon 8 SNP(G/A) | Cuevas et al. 2010 |
|  | GATGTCCACACCTTTCTGCC |  |  |  |  |  |
|  | CTTGCACCGCGGCTTGCC |  |  |  |  |  |
| SSIIaSNP3FF | GGGTGGGTGGGGTTCTCG | ASP | 6 | 6.75 | Exon 8 SNP(G/A) | Cuevas et al. 2010 |
|  | CACCATTGGTACTTGGCCTTGAC |  |  |  |  |  |
|  | GCGGGCTGAGGGACAGCA |  |  |  |  |  |
| SSIIb-1-F | AGATTTGAACTCAGGACTTGGTG | STS | 2 | 31.23 | Promoter 17bp Insert | Tian et al. 2010 |
|  | TCTATGGGCTCTATCCTTACTAGG |  |  |  |  |  |
| SSIIb-2-F | CGCTCGTTGCCTAGCTAGC | STS | 2 | 31.23 | 5'-UTR 3bp Insert | Tian et al. 2010 |
|  | GGCGAGGAAGCGATTGCC |  |  |  |  |  |
| SSIIb-3-F | ACAGTATGTTTGCCTCAGCG | STS | 2 | 31.23 | 5'-UTR 8bp Del | Tian et al. 2010 |
|  | GTAAATCCACCCAGCCAGTC |  |  |  |  |  |
| SSIIc-1-F | CACCCCACCGTTCTACTATGC | STS | 10 | 15.34 | Promoter 86bp&81bp Insert | Tian et al. 2010 |
|  | TCCATAGTTTCATTGAGATTGCTC |  |  |  |  |  |
| SSIIc-2-F | AGAGATCAAATCGTGGAAC | STS | 10 | 15.34 | 5'-UTR 6bp Del | Tian et al. 2010 |
|  | TGGAGTGAAGTAGTGGAAT |  |  |  |  |  |
| SSIIc-3-F | ATCTTTAGACGATTAGCG | STS | 10 | 15.34 | 3'-UTR 6bp Del | Tian et al. 2010 |
|  | AAGTCACAAGTAGAAGGG |  |  |  |  |  |
| SSIIc-IF | CCTATAAAGCTCTCTTATACAAACTAC | ARMS | 10 | 15.34 | Intron 3 SNP (G/C) | Tian et al. 2010 |
|  | CTAACATGAATGTCTACGGC |  |  |  |  |  |
|  | GGTTGCAGGTACATAAATTG |  |  |  |  |  |
|  | TTAAACTGTCTCGTGATGGTA |  |  |  |  |  |
| SSIIIa-F | GAACTTGTGCCTTAAGCTGACTG | STS | 8 | 5.35 | Intron 2 20bp Del | Tian et al. 2010 |
|  | GGAATAGTAAGCCGAAGGACTT |  |  |  |  |  |
| SSIIIa-IF | GGCTGTAACTGGAGTTTTCTAG | TSP | 8 | 5.35 | Intron 3 SNP(A/G) | Tian et al. 2010 |
|  | TCTAATACGGTGCTGAATGGAAA |  |  |  |  |  |
|  | TGGAGAAGGAACATAACAGGGAC |  |  |  |  |  |
| SSIIIb-F | AAGAAGGGAAGGGAGTCAGC | STS | 4 | 31.54 | 5'UTR 9bp Ins/3bp Ins/ 2bp Del | Tian et al. 2010 |
|  | GCCATCTCCATTGCCAGC |  |  |  |  |  |
| SSIIIb-IF | CCGTTTCTAGTTTATCTTCTGAAT | TSP | 4 | 31.54 | Promoter SNP(A/T) | Tian et al. 2010 |
|  | TGCTCTTATGGTGGAAATCACG |  |  |  |  |  |
|  | TGCAACGGACGGCTGCTC |  |  |  |  |  |
| SSIVa-IF | GACGCCGTTAACTTTTTCTAAT | ARMS | 1 | 30.37 | 5’-UTR SNP(T/C) | Tian et al. 2010 |
|  | CGAATGGCCAAACCTG |  |  |  |  |  |
|  | ATAACCCGCTGCTATTCTCT |  |  |  |  |  |
|  | CCGTTGACTTTTTCTCACAT |  |  |  |  |  |
| SSIVb-1-IF | CTGCTACAGTCCCTTTCG | ARMS | 5 | 26.4 | Intron 5 SNP (A/G) | Tian et al. 2010 |
|  | TGGGCCATGCTGAAT |  |  |  |  |  |
|  | GAAAGATAGGGAAATTGTGG |  |  |  |  |  |
|  | ACTTCGGTGAGAAACAAACT |  |  |  |  |  |
| SSIVb-2-IF | CACCATCAATCTGTTGAAAT | ARMS | 5 | 26.4 | Intron 12 SNP(C/T) | Tian et al. 2010 |
|  | ATGTGAAATGGACCCG |  |  |  |  |  |
|  | CAGGTTGGGTGTATTACAAG |  |  |  |  |  |
|  | ACATGCATATATTTTTCCCA |  |  |  |  |  |
| SSIVb-F | TTCCCTTGGTGGTGCGTG | STS | 5 | 26.4 | Intron 1, 22bp Del | Tian et al. 2010 |
|  | TAAAGCGTTCCGACAGTA |  |  |  |  |  |

TSP: temperature-switch PCR

ARMS: amplification refractory mutation system

ASP: allele specific primer

Supplementary table 4 Mapped QTLs or cloned genes for grain shape and yield components, marker information and references

| QTL | Marker | Forward Primer (5'-3') | Reverse Primer (5'-3') | Chr | Position(Mb) | References | Journal |
| --- | --- | --- | --- | --- | --- | --- | --- |
| *Gn1a* | RM3360 | ACTTACACAAGGCCGGGAAAGG | TGGTAGTGGTAACTCTACTCCGATGG | 1 | 5.23 | Ashikari et al. | Science 2005, 309: 741-745. |
|  | Gn1a-M1 | CTCTTGCTTCATTATCAATC | AAACTACACAAGAATCTGCT | 1 | 5.27 | Yan et al. | Euphytica 2009, 169: 215-226 |
|  | Gn1a-M2 | TGAGGATGCCGTGGAAGACG | TTCGTGTTCGCGCAGGACGT | 1 | 5.27 |  |  |
|  | RM10316 | AAGATCGCTGGGAGATCTGTAGG | GCATGCTAATTAGTCAGCCTTGG | 1 | 5.29 |  |  |
|  |  |  |  |  |  |  |  |
| *qSPP1* | RMG2746 | AAATGGGCTTCCTCCTCTTC | CAGCCTTGATCGGAAGTAGC | 1 | 6.15 | Zhang et al. | Theor. Appl. Genet. 2009, 118: 1035-1044 |
| *Gw1-1* | RM10376 | TTAGTTTAACCGCACCGTACACC | GGTCGTTGAATTGGTGTCAAGC | 1 | 6.28 | Yu et al. | Chinese J Rice Sci. 2008, 22: 465-471 |
| *SPP1* | YN27 | TACCACTGAACCCACGTGTC | GCTGCCTTTGTTCTCACGTT | 1 | 6.52 | Liu et al. | Theor. Appl. Genet. 2009, 118:1509-1517 |
| *qGL-1* | RM10390 | GCAACGTTACGTCTTGGCATGG | CCTCTCGCGTCTCTCTCAACG | 1 | 6.58 | Yu et al. | Chinese Sci. Bull. 2008, 53: 2326-2332 |
| *qGW-1* | YN34 | TGTGCCATATCTGTGCCTGT | TCGGTTAGGTCGGTTAATTCC | 1 | 6.63 |  |  |
|  | RM10398 | TCTCCTTGCTCTACTGCCTTTGG | TTCTGCAACTTGCCAAGAAGACC | 1 | 6.67 |  |  |
|  | RM10404 | CTGGAGTGGTTTCTCTCCTCTGC | TACTCTGCTCGCGTAACTTCTCC | 1 | 6.71 |  |  |
|  | RM1344 | CTGCAATCCGAGTAGGAAGC | TGAGCATTCACTCCGATCTG | 1 | 7.02 |  |  |
|  |  |  |  |  |  |  |  |
| *qGY2-1* | RM279 | CCTCTCACTCACGTGGACTCTCC | CCTCACCCTAGGCTTTGATATGC | 2 | 2.88 | He et al. | Genome Res. 2006, 16: 618-626 |
|  | SBG1-S | GGCGAGACTAATTTTAATCATGG | GAATACATGGGCCGTGTTTAGA | 2 | 2.98 |  |  |
|  |  |  |  |  |  |  |  |
| *tgw2* | RM12813 | TATTATGCAAGGGAGGGAGTACATGC | GTTGCTTGACTCGTTGACACTCC | 2 | 7.84 | Oh et al. | J. Crop Sci. Biotech. 13: 7-12 |
| *GW2* | RM12827 | GCTCTGGCACCGAGATTATTATAGC | GAGAGACTGCGACCTCTGTAGCC | 2 | 8.11 | Song et al. | Nature Genet. 2007, 39: 623-630 |
|  | W004 | ACCAGCATTCAGCCATTC | TTGATAGAGCAACCCAGT | 2 | 8.12 | Yoon et al. | Theor. Appl. Genet. 2006, 112: 1052-1062 |
|  | RM12833 | TCTGAGGCGAGGTAGGTTGTCG | GACATGAGCTCCTCCAGCTTCC | 2 | 8.16 |  |  |
|  | RM12836 | ATCGCTATTGCTAACCAACTCG | GGAAATTCTCTCATCCACACTGC | 2 | 8.22 |  |  |
|  | RM7288 | TTTCTCAACTGAAACAACAT | AGTTTAAGAGCGTTTCTAGG | 2 | 9.03 |  |  |
|  | STS5803.7 | GAATGGATGGATGGATCGAG | GTAGGGTCCGGCGAGATATG | 2 | 9.04 |  |  |
|  | RM13104 | AGACCACATAAAGCCCAAAGAGC | AATTTACTCGGACTATGGGAGTGG | 2 | 13.51 |  |  |
|  |  |  |  |  |  |  |  |
| *qSPP2* | RMG2762.HAU2762 | GACGGGTCAGCTACCGTAAG | TCCCCCCTCTAAAACCCTAG | 2 | 22.46 | Zhang et al. | Theor. Appl. Genet. 2009, 118: 1035-1044 |
|  |  |  |  |  |  |  |  |
| *qPDS3* | RM14820 | AGGTCGTCGATGTCCCTCTCC | AAACCATCGTGGCATCATCTCC | 3 | 10.79 | Tan et al. | Plant breeding 2010, 130: 177-184 |
|  | RM14823 | CCTGAATGGTTGAATCCTCTCTCC | ATTGTACTGGCATTGGTGGAACG | 3 | 10.82 |  |  |
|  |  |  |  |  |  |  |  |
| *gw3.1* | JL123 | GGGTACAACGAGAGGGATGA | CAGCCCAGCATAATTTAGCC | 3 | 16.61 | Li et al. | Genetics 2004, 168: 2187-2195 |
| *GS3* | RGS1 | TCCACCTGCAGATTTCTTCC | GCTGGTCTTGCACATCTCTCT | 3 | 16.69 | Wang et al. | Theor. Appl. Genet. 2011, 122: 905-913 |
| *GW3* | JL109 | TGGAGCTGTGGACTACTGGA | TCCCTGAGCCTACCTGTCAT | 3 | 16.70 | Guo et al. | J Integr. Plant Biol. 2009, 51: 45-51 |
|  | RM15206 | CATTTCTTTGCCCTCGATCTTTCC | AAGCGCCATAATCCAGGAACC | 3 | 16.77 |  |  |
|  | WGW16 | ACTTGACGAATCTTTATTTGCT | TGTGTTTCTTGGAATTTAGACTTA | 3 | 16.88 |  |  |
|  | WGW19 | ACCAAAATGGAATACCGAACG | TAACAACACGCAATAGAAGG | 3 | 16.93 |  |  |
|  | RM16 | CGCTAGGGCAGCATCTAAA | AACACAGCAGGTACGCGC | 3 | 23.08 |  |  |
|  |  |  |  |  |  |  |  |
| *TGW3b* | W3D16 | CCCGCTTGAAAACTAACAC | ACACGCCTAAGCAAATGTA | 3 | 30.26 | Liu et al. | Theor. Appl. Genet. 2010, 120: 933-942 |
|  | RM15885 | AAGAACGCCCTCGACATCAACG | ACTTGGACTCTCCCGTCGTCAGC | 3 | 30.27 |  |  |
|  | RM15948 | GAGAGTGGGTGAGAAGGATCAGC | ACAGCAGATTTGTTGGCATCTGG | 3 | 31.31 |  |  |
|  |  |  |  |  |  |  |  |
| *DWL1* | HL921 | ATGGCTTCAGACTTCAGAGT | CAAATTAACCTTCAGGCAAG | 3 | 35.12 | Jiang et al. | J. Genet. Genomics 2008, 35: 715-721 |
|  | HL944 | TGCCAGCCTAGCGAGCCTAA | ATTGCAGCGAGCTACACG | 3 | 35.16 |  |  |
|  |  |  |  |  |  |  |  |
| *Gnp4* | Y42 | AGTGTAGAACAGCACTGGAATC | AAGCTTAGCTACGCTAATGACC | 4 | 19.37 | Zhang et al. | Agr. Sci. China 2011, 10: 1825-1833 |
| *GIF1* | Y48 | TCACCATATGGAAGCATCAAG | TATGTGTTTGTTCATGTGCAC | 4 | 19.38 | Wang et al. | Nature genet. 2008, *40:* 1370-1374 |
|  | RM16942 | CCAGTACTCTCGCTCCACTCTCC | ATCGCTTTCACGTCACCAAGG | 4 | 20.37 |  |  |
|  |  |  |  |  |  |  |  |
| *htd1* | RM17307 | AGAGCTTGGAGGCACCAATACCG | AGAAAGAACTCCGGCCACCTTCG | 4 | 27.33 | Zou et al. | Planta 2006, 222: 604-612 |
|  |  |  |  |  |  |  |  |
| *qGN-1* | nkssr04-19 | CTGGAATCACAAACCACGAC | GCTACCTCAAGCTCCACGAC | 4 | 30.72 | Fan et al. | Afr. J Biotech. 2008, 7: 1707-1711 |
|  | RM3276 | TCCGTCTCGACTCTTCCATC | GATGAGACACCACGGACATG | 4 | 30.72 | Deshmukh et al. | Funct Integr Genomics 2010, 10: 339-347 |
|  |  |  |  |  |  |  |  |
| *GS5* | C62 | GATTGACTGATAAATTGACAGC | CTAACTCCCATGGAATTAC | 5 | 3.38 | Li et al. | Nature genet. 2011, 43: 1266-1269 |
| *qSW5* | RM3328 | TCTACTCGTGCAGCTTGTGC | GAAGCAGCAAGAAAGGGATG | 5 | 5.24 | Shomura et al. | Nature genet. 2008, 40: 1023-1028 |
| *gw5* | RM18033 | AGAGAGACGAGAGCTTGCTGTGC | TACTTCCTCCATTCCTGGTCTCC | 5 | 5.31 | Wan et al. | Genetics 2008, 179: 2239-2252 |
|  | MS40671 | TTTGATTGCCATTATCGAGTTAG | GTGTGCGTGAAGAGAACAGT | 5 | 5.34 |  |  |
|  | RM18035 | CCGTGGTGATAAACTGTTAACTGAGG | CGCATATGCATCAGCATCACG | 5 | 5.34 |  |  |
|  | RMw530 | GTTGGAGTGGAGACCAGGAA | CACCTTTCTACTTCCTCCA | 5 | 5.35 |  |  |
|  | RMw513 | GTATTTGTTTGTCGCATTC | TAGGACCATAGATGTGAGTTA | 5 | 5.36 |  |  |
|  | RMw534 | GACCGTGTCGCTGTTCAA | CTACCCTATCATCCAAGCAGTT | 5 | 5.36 |  |  |
|  |  |  |  |  |  |  |  |
| *qTGW7* | RM22034 | CCAGTTTATCTTCTGCACCTTCTCG | CCAGTTTATCTTCTGCACCTTCTCG | 5 | 26.35 | Bian et al. | Breeding Sci. 2010, *60:* 305-313 |
|  |  |  |  |  |  |  |  |
| *qPH6-1* | RM3414 | TAGGGCAATTGTGCAAGTGG | TTGGGAATTGGGTAGGACAG | 6 | 2.88 | Bao et al. | Chinese J Rice Sci. 2009, 23: 470-474 |
|  | RM19417 | CGGCTCCCTTGAATCTTCTGC | GGTGACGCCTTATGAATGTACGC | 6 | 2.98 |  |  |
|  |  |  |  |  |  |  |  |
| *NSP* | RM111 | CACAACCTTTGAGCACCGGGTC | ACGCCTGCAGCTTGATCACCGG | 6 | 5.10 | Gong et al. | Agr. Sci. China 2010, 9: 1085-1092 |
|  |  |  |  |  |  |  |  |
| *NGP* | RM19784 | GAGTTCTCTCCCTGGCTTCTGC | TCTTCAAACAGCCATCTGACACG | 6 | 9.44 | Gong et al. | Agr. Sci. China 2010, 9: 1085-1092 |
|  |  |  |  |  |  |  |  |
| *GW6* | RM7179 | CACGTGTCAGCTTAAGAGCG | TTACATCATAAGCCCGCAGG | 6 | 19.38 | Guo et al. | J Integr. Plant Biol. 2009, 51: 45-51 |
|  | RM20201 | TTAGAGGTAACGGAGGCACAACC | GATGGCTTGAGAGCGTTTGTAGG | 6 | 20.16 |  |  |
|  | RM3187 | TCCCCACATCGTGTCGTC | TTTTTCCCCTTCTACCCTCG | 6 | 20.58 |  |  |
|  |  |  |  |  |  |  |  |
| *Nop(t)* | M10 | GGATTTTTCTTTCCACCTTTG | AGGCATGAACTTGAAGTGAGTC | 6 | 24.28 | Wu et al. | Rice Sci. 2009, 16: 165-172 |
|  | M9 | AATGAATAGATTACCACATGCTA | TGCCTCTTATTTTACTTTTCTTT | 6 | 24.38 |  |  |
|  |  |  |  |  |  |  |  |
| *dep3* | RM30 | GGTTAGGCATCGTCACGG | TCACCTCACCACACGACACG | 6 | 27.25 | Qiao et al. | Theor. Appl. Genet. 2011, 122: 1439-1449 |
|  |  |  |  |  |  |  |  |
| *gpa7* | RM481 | TAGCTAGCCGATTGAATGGC | CTCCACCTCCTATGTTGTTG | 7 | 2.87 | Tian et al. | Theor. Appl. Genet. 2006, 113: 619-629 |
|  | ID52 | GTTTGGTGGTGTTCATGGTCT | GATCAGCTCTCACCAATCCAG | 7 | 2.88 |  |  |
|  |  |  |  |  |  |  |  |
| *Ghd7* | RM5436.2 | CAAAGGGGGTGTCCTCTATG | GTTGCTCGTCCTACATGTGC | 7 | 9.07 | Xing et al. | Theor. Appl. Genet. 2008, 116: 789-796 |
|  | RM5499 | GGACGAAAGGGTATTTGATTGG | CCTCAAGGTGGTCTCCTTCTCC | 7 | 9.99 |  |  |
|  |  |  |  |  |  |  |  |
| *qGL7-2* | InDel1 | CCATAGTAAGACGACCTT | GATATTCTGTCAGCAGTT | 7 | 24.66 | Shao et al. | J. Genet. Genomics. 2010, 37: 523-531 |
|  | RM21945 | CTACACAAGTGAACGCCATCAGG | GTTCTAGGGTGTCCTTTCATGAGC | 7 | 24.93 |  |  |
|  |  |  |  |  |  |  |  |
| *qGL7* | RM6389 | GACGAGGAGTTCGTCGCTAC | CCTTCTCCTTCGTCTCCTCC | 7 | 28.28 | Bai et al. | BMC Genet. 2010, 11:16 |
|  | RID711 | GCACATGCATGCTAGGACAT | AGCCGGTAAATTTCTTGCAC | 7 | 28.48 |  |  |
|  |  |  |  |  |  |  |  |
| *QSpp8* | RM310 | CCAAAACATTTAAAATATCATG | GCTTGTTGGTCATTACCATTC | 8 | 5.11 | Zhang et al. | Theor. Appl. Genet. 2006, 113: 361-368 |
|  | RM126 | CGCGTCCGCGATAAACACAGGG | TCGCACAGGTGAGGCCATGTCG | 8 | 5.22 |  |  |
|  |  |  |  |  |  |  |  |
| *gw8.1* | RM23201.CNR151 | GTTCTTTCCGGTGACGAGAC | CGCTGCAGATGAGCAGATAC | 8 | 21.52 | Xie et al. | Theor. Appl. Genet. 2006, 113: 885-894 |
|  | RM30000.CNR99 | AGCTCACCTCGTTTTGCGTA | CGGACAAATTCGTTCACCTC | 8 | 21.70 |  |  |
|  |  |  |  |  |  |  |  |
| *OsSPL16* | RM502 | GCGATCGATGGCTACGAC | ACAACCCAACAAGAAGGACG | 8 | 26.36 | Wang et al. | Nature genet. 2012, 44: 950-954 |
|  | PSM711 | ATGACCGTCTGCTTCCTCTAA | AACATCGACAGGGAGAAGTGC | 8 | 26.37 |  |  |
|  |  |  |  |  |  |  |  |
| *Ghd8* | RM15937 | GGAAGAACCTGCGTATCAAGACC | CCACACGGAAGCAGAATTAGCC | 8 | 30.94 | Yan et al. | Mol. Plant 2011, 4: 319-330 |
|  |  |  |  |  |  |  |  |
| *gw1* | RM24718.CNR111 | GACCAACGTGCATGTGACTT | GCTTGCACTAGGGCTCCTT | 9 | 20.90 | Xie et al. | Theor. Appl. Genet. 2008, 116: 613-622 |
| *gw1* | RM24718.CNR113 | GACCAACGTGCATGTGACTT | GCTTGCACTAGGGCTCCTT | 9 | 21.20 |  |  |
| *gw1* | RM30005.CNR142 | CGACTCCCACTCTCAGATCC | AGATTTTCACCATCGGTTGC | 9 | 21.22 |  |  |

Supplementary table 5 Mapped QTLs or cloned genes for chalkiness or related components, marker information and references

| QTL | Marker | Forward Primer (5'-3') | Reverse Primer (5'-3') | Chr | Position(Mb) | References | Journal |
| --- | --- | --- | --- | --- | --- | --- | --- |
| *qCH1 (t)* | RI02451 | CCCTCTACCTCCAGTTCTTCG | CATGCTGGTAGGCGTTGTACT | 1 | 0.6 | Tan et al. | Theor. Appl. Genet. 2000, 101: 823-829 |
| *qWK1-1* | RM10074 | GTCGCGGGTACATGACACAAGC | CGAGCTGTTACGGCAATCTCTATGG | 1 | 1.34 | Tabata et al. | Breeding Sci. 2007, 57: 47-52 |
| *qPGWC-1* | RM10092 | TTGACTCAGCTGAAACGATGACC | CAGGGTTAGACGGGAGCTTCG | 1 | 1.66 | Chen et al. | Afr. J. Biotech. 2011, 10: 6891-6903 |
|  | RM10129 | ATGCAATGTACGTAGCTACAGCATCC | TCTCAAACGTTCCCATGAACTCG | 1 | 2.52 |  |  |
|  | RI02341 | CGCATGGTTCAGTAGGTGTG | GTGTCTGTCCTGCACCTGAA | 1 | 2.77 |  |  |
|  | RM3604 | CAGGAACCAACCTTCTTCTTGACC | GTCAGACTCCGATCTGGGATGG | 1 | 5.14 |  |  |
|  |  |  |  |  |  |  |  |
| *qWK1-2* | RM3403 | CTGCCTCCTCCATTTCCCACTCC | CGAACGACTGCTCCCTCTTCAGC | 1 | 35.32 | Tabata et al. | Breeding Sci. 2007, 57: 47-52 |
| *qWBHT1 (t)* | RM8278 | ACTGCGAACTACACTTTCACTGTACC | GACTGACTCGCATGCTTTCC | 1 | 36.95 | Shirasawa et al. | Breeding Res. 2006, 8: 155 |
| *qWBe1 (t)* |  |  |  |  |  | Ebitani et al. | Jpn. J. Crop Sci. 2005, 74: 290-291 |
|  |  |  |  |  |  |  |  |
| *qBW2 (t)* | RM12740 | CATAAATGAGGGAGCGATGTGC | TGTCAGGGATGAGAAATGAGTCC | 2 | 6.86 | Ebitani et al. | Jpn. J. Crop Sci. 2005, 74: 290-291 |
| *qPGWC-2* | RM12878 | AAATGGACCAAGACGAACAGAGG | GTAGCAACGCACTGAATGTCAATAGG | 2 | 8.82 | Chen et al. | Afr. J. Biotech. 2011, 10: 6891-6903 |
|  |  |  |  |  |  |  |  |
| *qIK2 (t)* | RM13308 | CAAAGGCGGATTCTCATTAGACG | CAATCGACAGACACAGTTGTTCG | 2 | 18.9 | Terao et al. | Jpn. J. Crop Sci. 2004, 73: 96-97 |
|  | RI05333 | TGTTTATTTTTATTACAGTGCTTTGR | GCCAAGTGAGAAAGGGAAGA | 2 | 21.59 |  |  |
|  | RM13574 | TGCATGTTTGCCACAGTTAGGG | CGAGCCAAATTTGTGCACTCC | 2 | 23.64 |  |  |
|  | RM13589 | ACTCCCGATAGTAGCAAATAGG | ATGGACTTTAGTCTCCTCTTCC | 2 | 23.95 |  |  |
|  | RI04841 | ACGCATGCTGAGAAAATTCG | TCTCACGGAGACATTGCTTG | 2 | 24.19 |  |  |
|  |  |  |  |  |  |  |  |
| *qWK2* | RM14096 | GTGCATGAACGACATAGCAAAGG | TTCCGATCTGAATGCACACTGC | 2 | 33.87 | Tabata et al. | Breeding Sci. 2007, 57: 47-52 |
|  |  |  |  |  |  |  |  |
| *qPGWC-3* | RM3872 | GGCTCACCAAATTAAGAGCTTGC | TGATGATGCTTGCCTTAGTGTCC | 3 | 6.83 | Zhou et al. b | Acta Agronomica Sinica 2009, 35: 255-261 |
| *qIPGC-3* | RM1338 | TGCAAGTTGGACTTCAAAGAGG | TGGATTCCTTCTTCCTTTCTCTCC | 3 | 8.37 | Liu et al. | Afr. J. Biotech. 2013, 10: 2399-2405 |
|  |  |  |  |  |  |  |  |
| *qWB3 (t)* | RM15175 | CTCCCTCCAAGAGGGCAAACC | CTTTGACCGAGGTGGGAGAGG | 3 | 16.3 | Kobayashi et al. | Breeding Sci. 2007, 57: 107-116 |
|  |  |  |  |  |  |  |  |
| *qPGWC-3* | RM16171 | TTGAATCTTGGAGCCCATACTCC | TGGGCGAATTTGTGTATTGC | 3 | 35.24 | Chen et al. | Afr. J. Biotech. 2011, 10: 6891-6903 |
| *qSWC-3* | RM16232 | GACTCATCCGCTGAGAGTTTCG | GAGGTATCCGGAGAGGAAGAGG | 3 | 36.17 | He et al. | Theor. Appl. Genet. 1999, 98: 502–508 |
| *wca3.1* |  |  |  |  |  | Li et al. | Genome 2004, 47: 697–704 |
|  |  |  |  |  |  |  |  |
| ***GIF1**** | RM16868 | ATATTAGAGCATCTCCGACAGC | AATCTTGGAGCCCATACTCC | 4 | 19.28 | Wang et al. | Nature genet. 2008, *40:* 1370-1374 |
|  | RM5586 | AGATGGCTGGCCAACAGACTGG | ACAATGCCCATCCACTGCTTCC | 4 | 19.71 |  |  |
|  | INV1 | TGGATTTAGGCTTGTTTGAGC | CGTGACCTGCATGTTTCTCT | 4 | 20.23 |  |  |
|  | SSR9 | CTCGTCACTCTCACCACCAC | CCCACTGACAAGCACACGTA | 4 | 21.58 |  |  |
|  |  |  |  |  |  |  |  |
|  |  |  |  |  |  |  |  |
| *qWB4 (t)* | RM17363 | GGAGCAGGCATCTGATCTAGGG | CAGTTGGGCAACAGATCCATCC | 4 | 28.54 | Kobayashi et al. | Breeding Sci. 2007, 57: 107-116 |
| *qPGWC-4* | RM5478 | GGATCCAATGCGATGCTACTCC | CGAAACAGTCGCGATTAACTGG | 4 | 33.26 | Chen et al. | Afr. J. Biotech. 2011, 10: 6891-6903 |
| *qPGWC-4* | RM3466 | CAGGACGAGATCCAAGCCAAGG | TCGTCCTCGCCACCACTACTCC | 4 | 33.83 | Zhou et al. b | Acta Agronomica Sinica 2009, 35: 255-261 |
|  |  |  |  |  |  |  |  |
| *qCH5-1 (t)* | RM589 | GTGGCTTAACCACATGAGAAACTACC | TCACATCATTAGGTGGCAATCG | 5 | 1.38 | Tan et al. | Theor. Appl. Genet. 2000, 101: 823-829 |
| *qPGWC-5* | RM17867 | CATCGATAGAGCCAGATAGATGC | TTTCTACAGGAAGTCAGGAACG | 5 | 2.19 | Chen et al. | Afr. J. Biotech. 2011, 10: 6891-6903 |
|  | RI0399 | CCTATGTCGCCACTCATCG | CACAACAATGCAGTCCCATC | 5 | 3.29 | Li et al. | Nature Genet. 2014, 46: 398-404 |
|  | RM574 | GGCGAATTCTTTGCACTTGG | ACGGTTTGGTAGGGTGTCAC | 5 | 3.39 |  |  |
|  | RM17947 | GTTCTTGCCTTCTCGGCATGTGG | GGTCACTGATTCCACCATTCACACC | 5 | 3.48 |  |  |
|  | RM22658 | CTGCTTGTCTACCATATTGCCTATCC | CGGTGTGCAACCACATCTCC | 5 | 4.39 |  |  |
|  | RM18068 | CAGCACTGACCACACTGTAATGG | GAGTTTGGCTAGAACACATCATGG | 5 | 6.11 |  |  |
|  | RM18153 | CGTACGACCTATCCCTATGAGACG | TCCGGATCACATTTCTGGTTAGC | 5 | 7.92 |  |  |
|  |  |  |  |  |  |  |  |
| *qCH5-2 (t)* | RM18314 | TTGAATCTTGGAGCCCATACTCC | CCTATCGGATGATCCACTATCAGG | 5 | 13.15 | Tan et al. | Theor. Appl. Genet. 2000, 101: 823-829 |
| *qAPG5-1* | RM18360 | TCGAGACTGATCGGAGTTTAGGC | CGCTCCTCCCTAACACCTCTACG | 5 | 14.09 | Ebitani et al. | Jpn. J. Crop Sci. 2005, 74: 290-291 |
| *qWBe5 (t)* | RM6742 | CCAAGTTATCCAAGCTTCGTTTCG | AGAACGACCTTTCGAGGGAGAGC | 5 | 14.74 |  |  |
|  |  |  |  |  |  |  |  |
| *qAPG5-2* | RM18596 | CTTCTGGGTTCCTTCTTCTGG | GCATATAGCACAGGAGAATAGGC | 5 | 18.92 | Ebitani et al. | Jpn. J. Crop Sci. 2005, 74: 290-291 |
|  | RM163 | CGCCTTTATGAGGAGGAGATGG | AAACTCTTCGACACGCCTTGC | 5 | 19.17 |  |  |
|  | RM18751 | CCGTGTGTTGGCTTAGAATCAAGG | GCCACTTTCCAAACATCAGAAAGC | 5 | 21.11 |  |  |
|  | RM3575 | ACAGCCTCAAATTGTGAGCAAGG | GCTGTATGATCTGTATCCATCCATCC | 5 | 21.31 |  |  |
|  | RM18789 | GAGGGAGGGAGTACATATCTGATCG | ATCTAATACGTGGCGCCTGTAGC | 5 | 21.9 |  |  |
|  |  |  |  |  |  |  |  |
| ***PPDKB**** | PPDKBSSR1 | GCTTGAAACAAGCTAAAATACCG | CAGATTATTTGCTCACGCTATAAAA | 5 | 19.65 | Kang et al. | Plant J. 2005, 42: 901–911 |
|  |  |  |  |  |  |  |  |
| *qWC5 (t)* | RM31 | CGCTCCTCCACTCTTCTCCTACC | CGTGCAGAAAGTCCATTACTCTCC | 5 | 28.59 | Tan et al. | Theor. Appl. Genet. 2000, 101: 823-829 |
|  |  |  |  |  |  |  |  |
| *qWBHT6 (t)* | RM204 | CTAGCTAGCCATGCTCTCGTACC | CTGTGACTGACTTGGTCATAGGG | 6 | 3.17 | Zhou et al. b | Acta Agronomica Sinica 2009, 35: 255-261 |
| *qCH6 (t)* | RM253 | CCATCTCTGCCTCTGACTCACC | TCCTTCAATGGTCGTATCTTCTCC | 6 | 5.43 | Shirasawa et al. | Breeding Res. 2006, 8: 155 |
| *qPGWC-6* |  |  |  |  |  | Tan et al. | Theor. Appl. Genet. 2000, 101: 823-829 |
| *qPGWC-6b* |  |  |  |  |  | Zhou et al. a | Theor. Appl. Genet. 2009, 118: 581-590 |
| *qWC6 (t)* |  |  |  |  |  | Kobayashi et al. | Breeding Sci. 2007, 57: 107-116 |
| *qWB6 (t)* |  |  |  |  |  |  |  |
| *qGH6 (t)* |  |  |  |  |  |  |  |
|  |  |  |  |  |  |  |  |
| *qIPGC-6* | RM20372 | GGCTTGTTAGCTAGGGTTATTGG | GAACATTTGCTGATGTCTCACC | 6 | 23.93 | Liu et al. | Afr. J. Biotech. 2013, 10: 2399-2405 |
|  | RM162 | TTGTTCCAGTTCAGGTCTTGTGC | CCCTACAAACACCATAAGAAGCAACC | 6 | 24.04 |  |  |
|  |  |  |  |  |  |  |  |
| *qPGWC-6e* | RM20547 | CTCTTCTTCTTCTGTCCGTCTTGG | CCATCTTCATTACCGACCTCTGC | 6 | 27.03 | Zhou et al. b | Acta Agronomica Sinica 2009, 35: 255-261 |
|  | RM20564 | ATGGATGGATGGATCGATGG | CACTCTGCACTGAGAGCAACAGG | 6 | 27.57 |  |  |
|  | RM6926 | CGATCGGCCTATCTTTCTGTGC | GCTAGCAGTGGGATCATGTTTGG | 6 | 28.59 |  |  |
|  | RM3765 | ACACCATGACACCAAAGGAAGG | GGATGCTTCCAATCCTCTCACC | 6 | 29.21 |  |  |
|  | MID06029 | TGAAAGATAATAATCTGTAAGGCAGTT | GTGATTAGCGTTGCCACTGA | 6 | 30.28 |  |  |
|  |  |  |  |  |  |  |  |
| *qWBe7 (t)* | RM21950 | AACCTTGCACCATTCTCTTCTGG | GGAATGGTTTACATCTCCGATCC | 7 | 24.99 | Tan et al. | Theor. Appl. Genet. 2000, 101: 823-829 |
| *qPGWC-7* | RM21964 | AAGACAGCCTTCAAGGGATTTGG | GTACGTGCACCGAGCAGAGC | 7 | 25.29 | Zhou et al. a | Theor. Appl. Genet. 2009, 118: 581-590 |
|  | RM22020 | CGTTGAGACGGTCACCTAATGC | GCTCAAATGTTTGACACGAAGC | 7 | 26.28 |  |  |
|  |  |  |  |  |  |  |  |
| *qWK8* | RM408 | AATTGCCCAACGAGCTAACTTCC | TGAGCTGTTTGTGCTCTTCTACTTCG | 8 | 0.12 | Tabata et al. | Breeding Sci. 2007, 57: 47-52 |
|  | RM22215 | CGACACTCATCTTGGACCATCC | GAAGCAGCATGCATGGAATTAGC | 8 | 0.19 |  |  |
|  | RM22483 | GACCATGGTGTGAGTGTGACAGG | CAAGTCCTACCTCAACCGCTACC | 8 | 4.33 |  |  |
|  |  |  |  |  |  |  |  |
| *qIPGC-8* | RM22598 | GCCTAAAGGTCATTAATGCGATGG | AGGGCCTGTGTGCATAGGATACG | 8 | 6.32 | Liu et al. | Afr. J. Biotech. 2013, 10: 2399-2405 |
| *qPGWC-8* | RM22634 | TTTCACCACTGTAGTCTCTCTCC | CTCGACAGTTTCTTAGCTAGTCC | 8 | 7.32 | Chen et al. | Afr. J. Biotech. 2011, 10: 6891-6903 |
|  |  |  |  |  |  |  |  |
| *qPGWC-8* | RM23270 | TCCACGATCCATCTTGTATAGAGC | GACGAATACCTCACCCATTGC | 8 | 22.81 | Wan et al. | Theor. Appl. Genet. 2005, 110: 1334–1346 |
|  | RM23427 | AGGGAGTCGGAGACCATGACG | TACCGCGTATCATGTCCTTGACG | 8 | 25.21 | He et al. | Theor. Appl. Genet. 1999, 98: 502–508 |
|  | RM23422 | GTCGGTCACGAAGTTCAGATCC | TCAGGCAAAGTTGAAGATGGTAGC | 8 | 25.25 | Guo et al. | J. Integr. Plant Bio. 2011, 53: 598-607 |
|  |  |  |  |  |  |  |  |
|  |  |  |  |  |  |  |  |
| *qPGWC-9* | RM23748 | CTCTGGGAACAAACTTGACAATGC | CAATTTGCAGGCTATGCTACTGC | 9 | 3.09 | Chen et al. | Afr. J. Biotech. 2011, 10: 6891-6903 |
|  | RM23924 | TAATACGTGGCGCCTGTAATACC | GATCAGTGAACATGATGGAACACG | 9 | 7.43 | Wan et al. | Theor. Appl. Genet. 2005, 110: 1334–1346 |
|  |  |  |  |  |  |  |  |
| *qPGWC-10* | RM25557 | GAACGAACGTGAACGAGCTAGG | ACGTGGGTGATCGTGTCTGC | 10 | 17.3 | Zhou et al. b | Acta Agronomica Sinica 2009, 35: 255-261 |
| *qPGWC-10* | RM171 | AACGCGAGGACACGTACTTAC | ACGAGATACGTACGCCTTTG | 10 | 18.79 | Chen et al. | Afr. J. Biotech. 2011, 10: 6891-6903 |
| *qCH10 (t)* | RM6745 | TGTTCTCAACACAAAATTCTCT | ATCGTAAGCAAAGTGCATAA | 10 | 18.9 | Tan et al. | Theor. Appl. Genet. 2000, 101: 823-829 |
|  |  |  |  |  |  |  |  |
| *qPGWC-11b* | RM27007 | ATATGACATGTGCAGTGCTGTCC | CGCTTCACATTGTAACACACAGG | 11 | 22.22 | Chen et al. | Afr. J. Biotech. 2011, 10: 6891-6903 |
| *qPGWC-11a* | RM27073 | CCACGTGTCAGTCATCCATCTAGG | GGTCTGCTCGATTACCATCAAACTCC | 11 | 23.47 |  |  |
|  |  |  |  |  |  |  |  |
| *qBW12 (t)* | RM5927 | TGTATAGCCCGGAAGTATGATCC | TCTGGTCTCGTCTCTCATGTGC | 12 | 2.2 | Ebitani et al. | Jpn. J. Crop Sci. 2005, 74: 290-291 |
| *qPGWC-12* | RM27792 | GAAGAAGAGAGACTAGGGAGAAGACG | CTTGTACCAGCAATTCTCTGTCC | 12 | 7.1 | He et al. | Theor. Appl. Genet. 1999, 98: 502–508 |
| *wc12.1* |  |  |  |  |  | Li et al. | Genome 2004, 47: 697–704 |
|  |  |  |  |  |  |  |  |
| *qIK12 (t)* | RM28502 | CGAGCAGATCTGATGTCGTCTTCC | CTTTGCTTTGCATGCCTCACG | 12 | 23.41 | Terao et al. | Jpn. J. Crop Sci. 2004, 73: 96-97 |
| *: Coloned gene | |  |  |  |  |  |  |

Supplementary table 6 Random selected markers for big gaps on chromosomes

| Marker | Forward Primer (5'-3') | Reverse Primer (5'-3') | Chr | Position(Mb) |
| --- | --- | --- | --- | --- |
| RM228 | TCTAACTCTGGCCATTAGTCCTTGG | AAGTAGACGAGGACGACGACAGG | 1 | 21.98 |
| RM1095 | CCCATTCAGTTGATCCTGTC | GCAAAAGCAAGGATGGAGAC | 1 | 31.25 |
| RM3411 | CGTCCTCCAGATGGTCCAC | ATGGGACTCCCGTACTCCTC | 1 | 31.64 |
| RM414 | CAAGGAAGATCTTGTGGACCATGC | CTGCAGATGCAGAGGCAGAGG | 1 | 41.08 |
| RM12160 | ACGACGACCAATCCCAAGACG | AGCAAATCGGAGTACAGGATCAGC | 1 | 41.2 |
| RM14549 | CAGAACCCTAGCCTCCTCCTTCG | ACCGAAACGAAGCTTGGATAACTTGG | 3 | 5.38 |
| RM252 | TTCGCTGACGTGATAGGTTG | ATGACTTGATCCCGAGAACG | 4 | 25.34 |
| RM24422 | GTGGAGTCGTGGACTCACTGG | GCTTCCTCGCACCACTAATTATCC | 9 | 16.29 |
| RM24427 | ATCTCCCGCCACTTCTTCTCC | TTAGTCCCGGTTGGTGTATCTGC | 9 | 16.4 |
| RM311 | TGGTAGTATAGGTACTAAACAT | TCCTATACACATACAAACATAC | 10 | 9.49 |
| RM3152 | GGAAGAGGACAATCGACAG | GACTATCTTGAAAATTCCCATC | 10 | 9.77 |
| RM26307 | GCAAAGCCAATGTCCTTATCTCG | ATAATGGTTCCCTGACAGCAAGC | 11 | 7.17 |
| M8 | AGAGGAAACAAGTGTGCCCA | GTTGATGCGGTGGACAGG | 11 | 7.19 |
| RM26985 | CACAAGACAACCTTCAATGG | GGCTTAGGAGCGTTTATAGG | 11 | 21.38 |
| RM26987 | TGCCTGTATGAGCATCTTCTTCG | GCTCTTGTTCTTGCTGCTCTTGG | 11 | 21.81 |

Supplementary table 7 Number of marker-trait associations detected across 6 environments

| Marker | AC | GC | GL | GW | LWR | DEC | PGWC | HRY |
| --- | --- | --- | --- | --- | --- | --- | --- | --- |
| SSIIa-IF | 5 | 6 |  |  |  | 6 | 6 | 2 |
| RGS1 |  | 1 | 6 | 6 | 6 | 2 | 3 |  |
| RMw513 |  |  | 3 | 6 | 6 | 6 | 1 |  |
| SSIIaSNP2 |  | 6 |  |  |  | 6 | 6 |  |
| GBSSI-1-IF | 6 | 1 |  |  |  | 5 | 5 |  |
| SSIIa-F |  | 5 |  |  |  | 6 | 6 |  |
| Indel1 |  |  | 1 | 5 | 6 |  |  | 2 |
| RM15206 |  |  | 6 | 2 | 6 |  |  |  |
| RM18068 |  |  | 2 |  | 2 | 6 | 4 |  |
| GBSSI-4-IF | 6 | 6 |  |  |  |  |  |  |
| SSI-2-IF |  |  | 4 | 4 | 4 |  |  |  |
| RM21945 | 2 |  |  | 4 | 4 | 1 |  |  |
| RM5436.2 |  |  | 6 | 1 | 4 |  |  |  |
| GBSSI-3-IF |  | 1 |  |  |  | 4 | 4 | 1 |
| GBSSII-F |  | 2 | 5 |  | 3 |  |  |  |
| RI02451 |  | 1 |  |  |  | 4 | 3 | 1 |
| RM21950 |  |  |  | 4 | 4 |  |  | 1 |
| GBSSII-IF |  | 2 | 5 |  | 1 |  |  |  |
| RM111 | 6 | 1 |  |  |  | 1 |  |  |
| RM5499 |  |  | 5 |  | 3 |  |  |  |
| RM16 |  |  | 4 |  | 3 |  |  |  |
| RM18360 |  |  |  | 3 | 2 | 1 |  |  |
| RM21964 |  |  |  | 3 | 2 |  |  | 1 |
| BeIIb-1-F |  | 1 |  |  | 1 | 2 | 1 |  |
| BEIIb-IF |  | 1 |  |  | 2 | 2 |  |  |
| RM18751 |  |  | 4 |  | 1 |  |  |  |
| RM204 | 1 | 3 |  |  |  |  |  |  |
| SSI-1-F |  | 2 |  |  |  | 1 |  | 1 |
| PPDKBSSR1 |  |  | 1 | 1 | 1 |  |  |  |
| PUL-2-F |  |  |  |  |  | 2 | 1 |  |
| RM16942 |  | 1 | 1 |  |  |  |  | 1 |
| RM17307 |  |  |  |  |  | 1 | 1 | 1 |
| RM6742 |  | 1 |  |  |  | 1 | 1 |  |
| Y48 |  |  |  | 1 | 2 |  |  |  |
| ISA2-2-IF | 1 |  |  |  |  |  |  | 1 |
| PUL-6-F |  |  |  |  |  | 1 | 1 |  |
| RM13574 |  |  |  |  |  | 2 |  |  |
| RM15948 |  |  |  |  |  | 1 |  | 1 |
| RM20201 |  |  | 1 |  |  |  |  | 1 |
| RM3414 | 1 | 1 |  |  |  |  |  |  |
| AGPL1-IF |  | 1 |  |  |  |  |  |  |
| BeIIb-3-F |  |  |  |  |  | 1 |  |  |
| INVSSR1 |  | 1 |  |  |  |  |  |  |
| PUL-3-F |  | 1 |  |  |  |  |  |  |
| PUL-4-F |  | 1 |  |  |  |  |  |  |
| RI05333 |  | 1 |  |  |  |  |  |  |
| RM10074 |  |  |  |  |  |  |  | 1 |
| RM14820 |  |  |  |  |  |  |  | 1 |
| RM153 |  |  |  |  |  | 1 |  |  |
| RM16171 |  |  |  |  |  | 1 |  |  |
| RM16868 |  | 1 |  |  |  |  |  |  |
| RM19417 |  | 1 |  |  |  |  |  |  |
| RM22483 |  | 1 |  |  |  |  |  |  |
| RM228 |  |  |  |  |  | 1 |  |  |
| RM24718 |  | 1 |  |  |  |  |  |  |
| RM28502 |  |  |  |  |  | 1 |  |  |
| RM3575 |  |  | 1 |  |  |  |  |  |
| RM3765 |  |  |  |  |  | 1 |  |  |
| SSI-1-IF | 1 |  |  |  |  |  |  |  |
| SSI-2-F |  |  |  |  |  |  |  | 1 |
| SSIIIb-IF | 1 |  |  |  |  |  |  |  |
| SSIVb-1-IF |  |  |  |  |  | 1 |  |  |
| SSIVb-2-IF |  |  |  |  |  | 1 |  |  |
| SSR9 |  |  |  |  |  |  |  | 1 |
| STS5803.7 |  | 1 |  |  |  |  |  |  |

Markers with a significant marker-trait association are reported at *q^FDR^*<0.05.

Trait abbreviations are as in Table 1

Supplementary table 8 Effects of alleles of major marker associated with each trait across 6 environments

| Marker | Trait | Pop | Number | Allele | DS_N0_ | DS_N90_ | DS_N180_ | WS_N0_ | WS_N45_ | WS_N90_ |
| --- | --- | --- | --- | --- | --- | --- | --- | --- | --- | --- |
| GBSSI-1-IF | AC | Pop1 | 72 | 1 | 23.04±2.33 | 23.14±1.91 | 86.6±12.68 | 23.12±2 | 22.42±2.29 | 23.17±2.65 |
|  |  | Pop1 | 5 | 2 | 19.10±2.77 | 18.2±2.84 | 75.1±8.88 | 18.08±3.07 | 17.26±3.71 | 18.82±3.55 |
|  |  | Pop2 | 102 | 1 | 24.02±4.07 | 24.19±3.54 | 81.45±18.24 | 23.77±3.76 | 23.44±3.7 | 23.47±3.21 |
|  |  | Pop2 | 9 | 2 | 18.21±1.79 | 17.68±2.79 | 80.56±7.58 | 16.93±2.51 | 17.11±4.64 | 18.64±3.8 |
|  |  | Pop3 | 69 | 1 | 23.62±1.76 | 23.7±1.92 | 85.65±14.11 | 23.65±2.09 | 22.72±2.58 | 23.03±2.1 |
|  |  | Pop3 | 2 | 2 | 16.85±3.46 | 17.55±2.76 | 73±9.9 | 16.85±2.62 | 16.45±0.35 | 15.1±1.27 |
|  |  | Pop4 | 73 | 1 | 25.40±2.50 | 25.42±2.36 | 72.64±18.4 | 25.38±2.98 | 24.58±3.29 | 24.35±3.22 |
|  |  | Pop4 | 40 | 2 | 15.76±4.20 | 16.04±4.08 | 79.94±11.6 | 15.77±3.72 | 15.57±4.94 | 16.4±4.11 |
| RGS1 | GL | Pop1 | 73 | 2 | 6.71±0.16 | 6.71±0.15 | 6.79±0.18 | 6.75±0.17 | 6.79±0.16 | 6.81±0.17 |
|  |  | Pop2 | 6 | 1 | 5.46±0.20 | 5.54±0.42 | 5.77±0.45 | 5.89±0.51 | 5.93±0.54 | 5.83±0.57 |
|  |  | Pop2 | 102 | 2 | 6.62±0.24 | 6.64±0.24 | 6.72±0.2 | 6.66±0.2 | 6.72±0.25 | 6.72±0.27 |
|  |  | Pop3 | 66 | 2 | 6.66±0.16 | 6.67±0.15 | 6.75±0.18 | 6.70±0.17 | 6.72±0.22 | 6.74±0.24 |
|  |  | Pop4 | 16 | 1 | 5.68±0.45 | 5.75±0.54 | 5.77±0.5 | 5.66±0.32 | 6.03±0.61 | 5.92±0.55 |
|  |  | Pop4 | 93 | 2 | 6.66±0.23 | 6.66±0.21 | 6.71±0.21 | 6.72±0.21 | 6.72±0.30 | 6.75±0.21 |
|  | GW | Pop1 | 73 | 2 | 2.04±0.11 | 2.03±0.11 | 2.04±0.12 | 2.01±0.11 | 2.03±0.11 | 2.04±0.10 |
|  |  | Pop2 | 6 | 1 | 2.30±0.29 | 2.29±0.30 | 2.28±0.32 | 2.23±0.31 | 2.27±0.27 | 2.28±0.33 |
|  |  | Pop2 | 102 | 2 | 2.09±0.10 | 2.08±0.12 | 2.07±0.11 | 2.05±0.09 | 2.06±0.11 | 2.07±0.11 |
|  |  | Pop3 | 66 | 2 | 2.07±0.09 | 2.06±0.10 | 2.07±0.09 | 2.04±0.09 | 2.08±0.10 | 2.08±0.11 |
|  |  | Pop4 | 16 | 1 | 2.38±0.27 | 2.34±0.27 | 2.37±0.27 | 2.36±0.22 | 2.29±0.24 | 2.33±0.19 |
|  |  | Pop4 | 93 | 2 | 2.10±0.12 | 2.09±0.14 | 2.10±0.14 | 2.04±0.13 | 2.08±0.15 | 2.08±0.14 |
|  | LWR | Pop1 | 73 | 2 | 3.31±0.21 | 3.32±0.22 | 3.34±0.25 | 3.36±0.23 | 3.35±0.22 | 3.34±0.22 |
|  |  | Pop2 | 6 | 1 | 2.40±0.33 | 2.46±0.40 | 2.57±0.41 | 2.68±0.42 | 2.65±0.41 | 2.61±0.50 |
|  |  | Pop2 | 102 | 2 | 3.18±0.20 | 3.19±0.21 | 3.25±0.2 | 3.26±0.17 | 3.28±0.21 | 3.26±0.22 |
|  |  | Pop3 | 66 | 2 | 3.23±0.18 | 3.24±0.16 | 3.27±0.17 | 3.28±0.17 | 3.25±0.20 | 3.25±0.23 |
|  |  | Pop4 | 16 | 1 | 2.44±0.50 | 2.51±0.52 | 2.48±0.50 | 2.42±0.35 | 2.69±0.52 | 2.57±0.42 |
|  |  | Pop4 | 93 | 2 | 3.18±0.23 | 3.20±0.25 | 3.21±0.25 | 3.30±0.24 | 3.26±0.30 | 3.25±0.25 |
| GBSSI-4-IF | GC | Pop1 | 8 | 1 | 72.81±15.62 | 83.06±12.41 | 79.94±9.66 | 82.44±15.35 | 77.13±22.14 | 80.5±15.75 |
|  |  | Pop1 | 66 | 2 | 85.23±15.27 | 86.13±12.87 | 87.98±13.16 | 89.58±10.82 | 80.67±14.99 | 81.93±15.59 |
|  |  | Pop2 | 27 | 1 | 69.44±19.82 | 67.76±20.84 | 68.24±18.33 | 73.93±17.68 | 66.52±20.73 | 65.5±20.05 |
|  |  | Pop2 | 84 | 2 | 81.80±14.57 | 86.09±13.36 | 86.73±41.4 | 85.81±13.16 | 81.77±16.38 | 77.82±17.91 |
|  |  | Pop3 | 65 | 2 | 87.14±12.71 | 85.25±13.89 | 88.02±13.91 | 88.75±12.57 | 82.87±17.26 | 82.08±17.89 |
|  |  | Pop4 | 33 | 1 | 59.91±20.01 | 64.45±18.63 | 68.39±20.96 | 67.68±16.87 | 62.67±20.92 | 65.88±19.85 |
|  |  | Pop4 | 74 | 2 | 78.07±16.94 | 79.47±13.73 | 77.27±15.05 | 80.75±14.02 | 79.11±14.74 | 77.71±17.43 |
| SSIIa-IF | DEC | Pop1 | 8 | 1 | 14.68±6.52 | 10.33±5.14 | 7.83±6.23 | 20.86±11.5 | 18.74±8.04 | 15.46±11.13 |
|  |  | Pop1 | 68 | 2 | 28.32±12.09 | 18.8±11.89 | 11.34±8.48 | 31.53±13.31 | 26.25±12.45 | 20.68±12.33 |
|  |  | Pop2 | 38 | 1 | 15.32±14.95 | 13.37±14.88 | 6.72±7.20 | 16.76±16.29 | 14.19±12.76 | 10.81±8.92 |
|  |  | Pop2 | 72 | 2 | 27.15±13.77 | 20.14±12.53 | 13.31±11.08 | 31.12±14.11 | 24.72±12.74 | 21.92±12.01 |
|  |  | Pop3 | 9 | 1 | 11.03±7.27 | 8.26±3.72 | 6.08±3.72 | 16.66±7.83 | 16.10±12.83 | 16.03±9.69 |
|  |  | Pop3 | 61 | 2 | 25.32±14.47 | 18.62±11.46 | 12.36±11.5 | 27.94±13.18 | 22.51±13.11 | 22.32±12.59 |
|  |  | Pop4 | 63 | 1 | 11.76±12.37 | 9.97±10.87 | 6.69±9.11 | 12.81±11.63 | 14.07±12.49 | 10.29±11.26 |
|  |  | Pop4 | 49 | 2 | 25.39±14.79 | 17.82±11.74 | 12.97±10.76 | 28.63±12.43 | 26.25±14.69 | 23.11±11.53 |
| SSIIa-IF | PGWC | Pop1 | 8 | 1 | 68.43±29.47 | 50.16±29.43 | 35.04±29.18 | 76.89±28.86 | 76.18±25.92 | 60.38±32.96 |
|  |  | Pop1 | 68 | 2 | 91.48±16.0 | 73.1±29.94 | 53.05±32.94 | 91.02±19.79 | 86.19±23.0 | 76.06±29.46 |
|  |  | Pop2 | 38 | 1 | 53.79±37.21 | 47.03±34.58 | 32.13±29.71 | 55.22±36.9 | 52.38±32.81 | 44.63±30.43 |
|  |  | Pop2 | 72 | 2 | 86.77±22.72 | 75.42±28.99 | 53.75±36.62 | 89.76±21.15 | 81.8±25.72 | 79.16±27.53 |
|  |  | Pop3 | 9 | 1 | 58.21±35.27 | 48.03±28.74 | 31.64±20.91 | 73.09±29.31 | 65.21±41.82 | 70.27±30.20 |
|  |  | Pop3 | 61 | 2 | 84.30±23.84 | 74.16±28.87 | 53.57±33.43 | 87.75±22.66 | 79.16±26.71 | 79.6±28.89 |
|  |  | Pop4 | 63 | 1 | 45.18±35.34 | 41.78±34.89 | 28.6±30.86 | 50.86±36.18 | 52.09±36.29 | 40.17±34.51 |
|  |  | Pop4 | 49 | 2 | 83.44±25.96 | 69.63±32.58 | 56.01±34.96 | 89.69±21.70 | 84.06±25.12 | 82.49±26.95 |

Trait abbreviations are as in Table 1
