# Supplementary material for: The Usefulness of Known Genes/Qtls for Grain Quality Traits in an Indica Population of Diverse Breeding Lines Tested using Association Analysis
Source: Rice (N Y). 2015 Sep 21;8:29. doi: 10.1186/s12284-015-0064-3 (PMC4577492; doi:10.1186/s12284-015-0064-3)
Supplement: Additional file 2: — Figure S1. Distribution of markers used in this study and identified significant marker-trait associations on chromosomes. Figure S2. Effects of allele combinations between SSIIa-IF and PUL-4-F on PGWC. (DOCX 122 kb) [file 12284_2015_64_MOESM2_ESM.docx]

Chr01 Chr02 Chr03

Chr04 Chr05 Chr06

Chr07 Chr08 Chr09

Chr10 Chr11 Chr12

Supplementary Figure 1

Distribution of markers used in this study and identified significant marker-trait associations on chromosomes.

Markers with red, blue and black colour represent markers for grain shape, starch biosynthesis and chalkiness related QTLs/genes. Markers with pink colour are randomly selected. Traits in brackets after marker represent significant association were detected at *q^FDR^*<0.05.


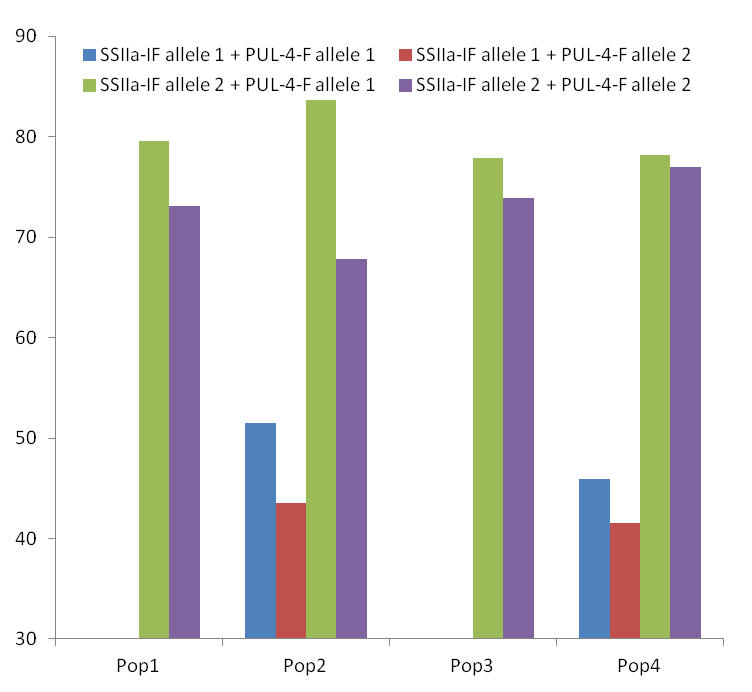


PGWC (%)

Supplementary Figure 2

Effects of allele combinations between SSIIa-IF and PUL-4-F on PGWC
